# The causal relationship between human brain morphometry and knee osteoarthritis: a two-sample Mendelian randomization study

**DOI:** 10.3389/fgene.2024.1420134

**Published:** 2024-07-08

**Authors:** Yongming Liu, Chao Huang, Yizhe Xiong, Xiang Wang, Zhibi Shen, Mingcai Zhang, Ningyang Gao, Nan Wang, Guoqing Du, Hongsheng Zhan

**Affiliations:** ^1^ Shi’s Center of Orthopedics and Traumatology, Shuguang Hospital Affiliated to Shanghai University of Traditional Chinese Medicine, Shanghai, China; ^2^ Institute of Traumatology and Orthopedics, Shanghai Academy of Traditional Chinese Medicine, Shanghai, China; ^3^ Yunyang County People’s Hospital Rehabilitation Medicine Department, Chongqing, China; ^4^ Department of Traditional Chinese Medicine, Shanghai Yangzhi Rehabilitation Hospital (Yangzhi Affiliated Rehabilitation Hospital), School of Medicine, Tongji University, Shanghai, China

**Keywords:** knee osteoarthritis, Mendelian randomization, brain-wide morphometric variations, brain-wide volumes, brain morphometries, two-sample Mendelian randomization

## Abstract

**Background:**

Knee Osteoarthritis (KOA) is a prevalent and debilitating condition affecting millions worldwide, yet its underlying etiology remains poorly understood. Recent advances in neuroimaging and genetic methodologies offer new avenues to explore the potential neuropsychological contributions to KOA. This study aims to investigate the causal relationships between brain-wide morphometric variations and KOA using a genetic epidemiology approach.

**Method:**

Leveraging data from 36,778 UK Biobank participants for human brain morphometry and 487,411 UK Biobank participants for KOA, this research employed a two-sample Mendelian Randomization (TSMR) approach to explore the causal effects of 83 brain-wide volumes on KOA. The primary method of analysis was the Inverse Variance Weighted (IVW) and Wald Ratio (WR) method, complemented by MR Egger and IVW methods for heterogeneity and pleiotropy assessments. A significance threshold of *p* < 0.05 was set to determine causality. The analysis results were assessed for heterogeneity using the MR Egger and IVW methods. Brain-wide volumes with Q_pval < 0.05 were considered indicative of heterogeneity. The MR Egger method was employed to evaluate the pleiotropy of the analysis results, with brain-wide volumes having a *p*-value < 0.05 considered suggestive of pleiotropy.

**Results:**

Our findings revealed significant causal associations between KOA and eight brain-wide volumes: Left parahippocampal volume, Right posterior cingulate volume, Left transverse temporal volume, Left caudal anterior cingulate volume, Right paracentral volume, Left paracentral volume, Right lateral orbitofrontal volume, and Left superior temporal volume. These associations remained robust after tests for heterogeneity and pleiotropy, underscoring their potential role in the pathogenesis of KOA.

**Conclusion:**

This study provides novel evidence of the causal relationships between specific brain morphometries and KOA, suggesting that neuroanatomical variations might contribute to the risk and development of KOA. These findings pave the way for further research into the neurobiological mechanisms underlying KOA and may eventually lead to the development of new intervention strategies targeting these neuropsychological pathways.

## Introduction

Knee Osteoarthritis (KOA) is a chronic degenerative disease that predominantly affects the articular cartilage of the knee joint, leading to significant pain, disability, and diminished quality of life among affected individuals (Wu et al., 2022; Scaturro et al., 2023). As one of the most common forms of arthritis, KOA imposes a substantial burden on individuals and healthcare systems worldwide (Guo et al., 2022). The global prevalence of KOA is increasing, paralleling the aging population and the rising rates of obesity, two of the principal risk factors for the development of this condition (Du et al., 2023; Long et al., 2023). According to recent epidemiological studies, KOA has a global prevalence of more than 15% and a prevalence of more than 40% among people over 40 years of age, making it a leading cause of disability among older adults (Zhang et al., 2023). The impact of KOA extends beyond the physical domain, encompassing psychological, social, and economic dimensions. Individuals with KOA often experience limitations in mobility and daily activities, leading to a loss of independence and increased reliance on healthcare resources (Almhdie-Imjabbar et al., 2023). The economic burden of KOA is equally significant, encompassing direct costs related to medical care and indirect costs due to lost workdays and decreased productivity (Wang H-N. et al., 2022). Despite its widespread prevalence and the multifaceted challenges it presents, the etiology of KOA remains incompletely understood, and current treatment options are primarily focused on symptom management rather than disease modification. The complexity of KOA, influenced by a combination of genetic, biomechanical, and environmental factors, underscores the need for innovative approaches to unravel the underlying mechanisms of the disease (Amirkhizi et al., 2022; Song et al., 2022). The exploration of the neuropsychological contributions to KOA, particularly the role of brain morphometry, represents a promising new direction in understanding the etiology and progression of this debilitating condition.

Human brain morphometry, the study of the shape and size of the structures within the human brain, plays a crucial role in understanding the complex relationship between brain anatomy and function (Fujita et al., 2021). Brain-wide volumes, which encompass the quantification of the overall size and specific regions within the brain, offer invaluable insights into the neural underpinnings of a wide range of cognitive functions, behaviors, and disease processes (Provenzi et al., 2022). The significance of brain-wide volumes extends beyond basic neuroscience, as variations in these measures have been associated with numerous neuropsychiatric disorders, cognitive abilities, and even predispositions to certain physical health conditions (Jansen et al., 2020; Migliore et al., 2022). The human brain, with its intricate architecture and vast network of neurons, is the central organ of the human nervous system, responsible for processing, integrating, and coordinating the information it receives from the sensory organs, and making decisions as to the instructions sent to the rest of the body (Sundqvist et al., 2022; Kurowska et al., 2023). The structural variations within the brain—encompassing areas such as the hippocampus, cingulate cortex, temporal lobes, and frontal lobes—are critically involved in functions ranging from memory and emotion regulation to decision-making and motor control (Fermin et al., 2022; Romaus-Sanjurjo et al., 2022). Brain-wide volumes can thus serve as biomarkers for cognitive and emotional health, as well as predictors of neurodevelopmental trajectories and outcomes in neurodegenerative diseases.

In the context of KOA, exploring the potential links between brain morphometry and KOA is grounded in the understanding that the brain plays a pivotal role in pain perception, the regulation of inflammatory responses, and motor function. The experience of pain, a primary symptom of KOA, is not merely a direct transmission of sensory information but involves complex processing by the brain, which can modulate the perception of pain through a variety of pathways (Chan and Chan, 2022; Stausholm et al., 2022). Pain-related disorders are related to brain structure and function (Cha et al., 2020; Yin et al., 2020). In patients with trigeminal neuralgia, cortical features such as reductions in cortical thickness, gyratory index, and sulcus depth were mainly located in the frontal regions, as opposed to controls (Li et al., 2021). Furthermore, patients with chronic back pain have been reported to exhibit a reduction in prefrontal cortical gray matter volume, which is negatively correlated with pain intensity (Fritz et al., 2016). This suggests that variations in brain-wide volumes may influence the susceptibility to or severity of KOA through mechanisms related to pain processing, inflammation, or even the regulation of physical movements that could exacerbate joint wear and tear. Furthermore, the brain’s role in coordinating motor functions implies that structural variations could impact the biomechanics of knee joint loading, potentially influencing the development or progression of KOA. For instance, alterations in the motor cortex or cerebellum might affect gait and balance, leading to abnormal stress distribution on the knee joint and contributing to cartilage degeneration over time (Simis et al., 2021; Sanchis-Alfonso et al., 2023). Therefore, the investigation into the relationship between brain morphometry and KOA represents an interdisciplinary convergence of neurology, psychology, and rheumatology, aiming to unravel the complex interactions between brain structure and function in the context of physical health. By identifying specific brain-wide volumes associated with KOA, researchers open new pathways for understanding the disease’s etiology and progression.

The rationale for selecting specific brain regions in our study stems from the understanding that certain neuroanatomical variations are implicated in pain processing, inflammatory regulation, and motor function—all critical aspects in the pathogenesis of KOA. For instance, the parahippocampal gyrus, cingulate cortex, and orbitofrontal cortex are regions known to be involved in the modulation of pain and the emotional response to chronic pain conditions (Toledo et al., 2020; Zhang et al., 2022). The transverse temporal gyrus is associated with sensory processing, which could influence pain perception (Findlater et al., 2018; Jacob and Nienborg, 2018). Additionally, the paracentral lobule plays a role in motor function and sensory integration, potentially affecting knee joint biomechanics and gait (Sie et al., 2019; Kornelsen et al., 2023). Variations in these brain regions may thus contribute to the differential experiences of pain and functional impairment seen in KOA patients. Understanding these relationships can provide insights into how neuroanatomical factors might predispose individuals to KOA or exacerbate its severity through mechanisms related to pain perception and motor control.

Mendelian randomization (MR) is a powerful epidemiological method that utilizes genetic variants as instrumental variables to infer causal relationships between potentially modifiable risk factors and health outcomes (Xiong et al., 2022; Zhou et al., 2023). The primary hypothesis of this study is that variations in specific brain-wide volumes causally influence the risk and development of KOA. By leveraging Mendelian Randomization (MR) methods, we aim to elucidate whether these neuroanatomical differences are not merely associated with, but indeed causative of, KOA. Specifically, our objectives are to identify brain regions with significant causal associations with KOA, assess the robustness of these associations through tests of heterogeneity and pleiotropy, and explore the potential mechanisms by which brain morphometry impacts KOA pathogenesis. By achieving these aims, we hope to advance the understanding of KOA’s neurobiological underpinnings and lay the groundwork for novel intervention strategies that target these brain-behavior relationships, potentially leading to more effective treatments for KOA. Therefore, we applied a two-sample Mendelian Randomization (TSMR) approach to explore the causal effects of 83 brain-wide volumes on the risk and development of KOA. The choice of brain-wide volumes as our focus of study is predicated on the hypothesis that certain neuroanatomical variations may play a role in the pathogenesis of KOA, possibly through mechanisms related to pain perception, inflammatory regulation, or motor function. Our analysis employed the Inverse Variance Weighted (IVW) and Wald Ratio (WR) methods as primary tools for causal inference, with additional tests for heterogeneity and pleiotropy to ensure the robustness of our findings (Wan et al., 2023). This study not only advances our understanding of KOA’s etiology but also exemplifies the utility of Mendelian Randomization in dissecting the complex interplay between the brain and body, offering insights that could have significant implications for the prevention and management of KOA.

## Materials and methods

### Data source and instrumental variables selection

The exposure data set comprised genome‐wides association study (GWAS) data for 83 cortical and subcortical gray‐matter volumes of 36,778 European from UK Biobank (33 cortical Desikan‐Killiany regions in each hemisphere, 8 subcortical regions in each hemisphere and a brain stem). GWAS effects were fitted in a linear mixed model using REGENIE. These single nucleotide polymorphisms (SNPs) and their corresponding effect sizes on inflammatory factor levels were obtained from genome-wide association studies conducted by the European Bioinformatics Institute (EBI, https://www.ebi.ac.uk/). The details of exposure data set, including brain GWAS phenotypes and UKB field IDs, are listed in Supplementary Table S1. The outcome data set was sourced from a genome-wide association study for KOA (ebi-a-GCST007090, 77,052 cases and 378,169 controls from UK Biobank). This study adhered to the following criteria for instrumental variable (IV) selection (Wu et al., 2022), Each SNP was required to exhibit a significant correlation with 83 cortical and subcortical gray‐matter volumes, surpassing the genome-wide significance threshold (P < 5E-8) (Scaturro et al., 2023). Evaluation of linkage disequilibrium (LD) among SNPs was conducted utilizing European sample data sourced from the 1,000 Genomes Project reference panel, wherein only the most statistically significant SNPs were retained, excluding those exhibiting an R2 value <0.001 within a clumping window of 10,000 kb (Guo et al., 2022). SNPs with a minor allele frequency (MAF) of ≤0.01 were excluded to mitigate potential bias. Subsequently, the definitive set of SNPs conforming to these selection criteria was identified and employed as instrumental variables within the framework of the TSMR analysis.

### Mendelian randomization

Subsequently, this study extracted relevant data concerning instrumental variables (IVs) from the exposure and outcome datasets, including *p*-values, standard errors (SE), and β coefficients. For exposures with only one IV, the Wald ratio (WR) method was employed (Moncla et al., 2022). The WR is calculated as the ratio of the effect estimate of the exposure on the outcome to its standard error (Marouli et al., 2019; Wang et al., 2022). This method provides a measure of the precision of the estimate and allows for hypothesis testing regarding the causal relationship between exposure and outcome. For exposures with two or more IVs, the study utilized the Inverse Variance Weighted (IVW) method. The IVW method operates by utilizing the reciprocal of the variance of each instrumental variable’s effect estimate as a weight, thereby assigning more weight to more precisely estimated effects (Li et al., 2022). This method aggregates the weighted effect sizes of genetic variants to provide an overall estimate of the causal impact of the exposure on the outcome (Chen et al., 2022). The MR Egger method, weighted median method, simple mode method, and weighted mode method are Supplementary Methods. The MR Egger method is a statistical approach used in MR to assess and correct for pleiotropy by allowing for an intercept term in the regression, providing an unbiased estimate even with invalid instruments (Li et al., 2022; Yang et al., 2023). The weighted median method offers a consistent causal estimate even if up to 50% of the genetic instruments are invalid by calculating the median of the individual ratio estimates, weighted by their inverse variance (Peters et al., 2021). The simple mode method groups genetic instruments into clusters and estimates the causal effect as the mode of these cluster-specific estimates, useful for mitigating pleiotropy. The weighted mode method refines this by assigning weights based on the inverse variance of each instrument and determining the mode of the weighted estimates to enhance robustness and reliability in the presence of heterogeneous instruments (Verkouter et al., 2020). Brain-wide volumes with *p*-values < 0.05 were deemed to exhibit significant causal associations with KOA.

### Sensitivity analysis and visualization

Heterogeneity refers to the variability in causal estimates across different genetic variants used as IVs, indicating potential inconsistencies in causal effects (Huang et al., 2022). The analysis results were assessed for heterogeneity using the MR Egger and IVW methods. Brain-wide volumes with Q_pval <0.05 were considered indicative of heterogeneity. Pleiotropy refers to the phenomenon where a genetic variant affects multiple phenotypic traits, potentially confounding the causal estimates between the exposure and outcome of interest in MR analysis (Chen et al., 2022). The MR Egger method was employed to evaluate the pleiotropy of the analysis results, with brain-wide volumes having a *p*-value < 0.05 considered suggestive of pleiotropy. Furthermore, the leave-one-out method was utilized to evaluate the robustness and potential impact of individual variants on the analysis results, by iteratively removing one genetic variant at a time to assess its influence on the overall causal estimate (Akbari et al., 2020). The scatter plots, forest plots and leave-one-out plots were employed to visualize the TSMR analysis results.

## Results

Instrumental variables were extracted from GWAS data sets of 83 cortical and subcortical gray‐matter volumes based on the principles established in this study. In our study, the selection of IVs required that each SNP exhibit a significant correlation with brain-wide volumes, surpassing the genome-wide significance threshold (*P* < 5E-8). However, two brain-wide volumes did not have any SNPs meeting this criterion, thus lacking enough instrumental variables. Information on the instrumental variables, along with their *p* values, standard errors (SE), and β coefficients in the exposure and outcome, is presented in Supplementary Table S2. Among them, Left caudal anterior cingulate volume and Right caudal anterior cingulate volume had only one available IV, thus being analyzed using the Wald ratio to assess their causal relationship with KOA. Additionally, 79 phenotypes had two or more IVs, hence five methods, including IVW, MR Egger method, Weighted median method, Simple mode method, and Weighted mode method, were employed to analyze their causal relationship with KOA.

**FIGURE 1:**
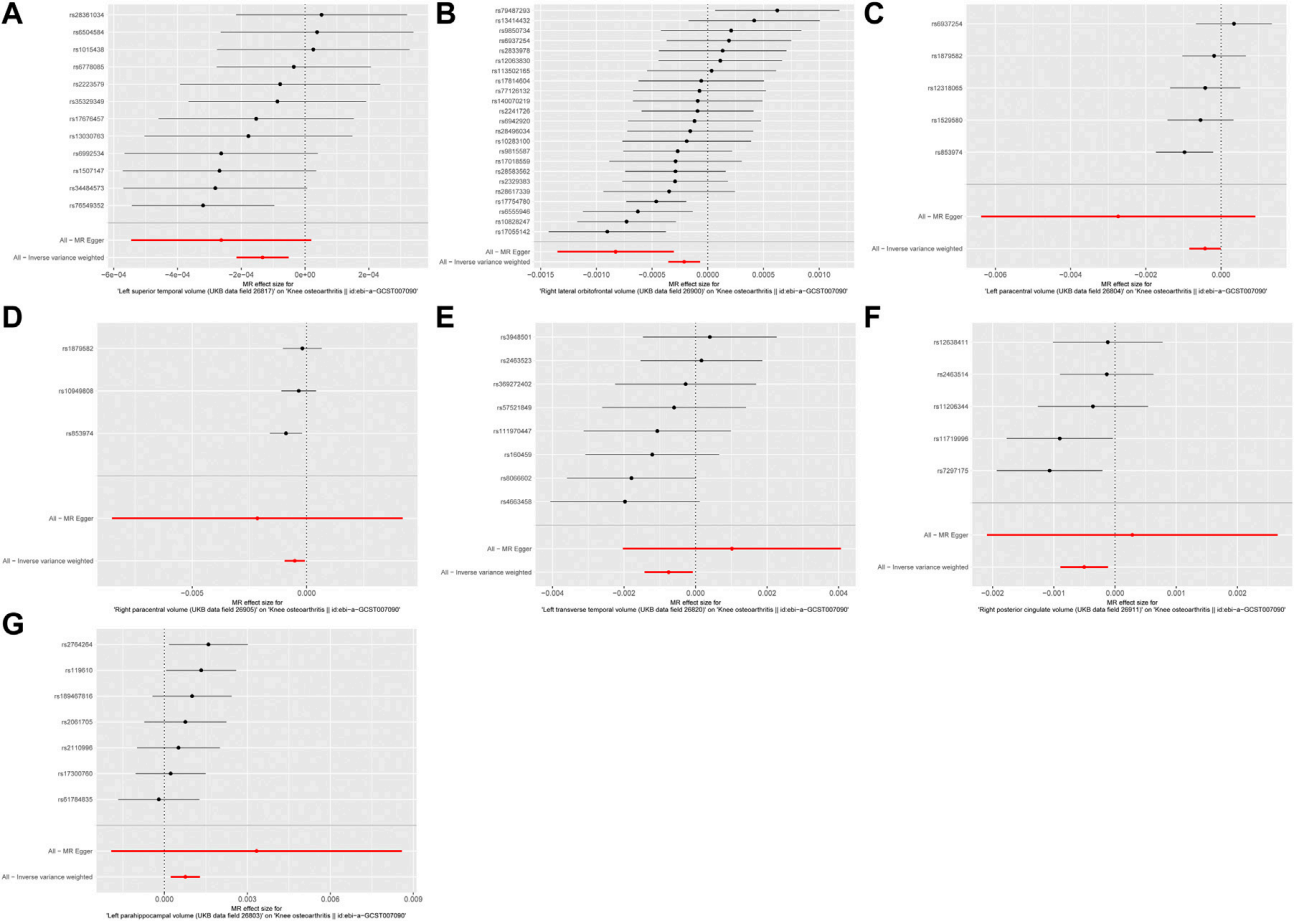
The forest plot of MR results. **(A)** Left superior temporal volume. **(B)** Right lateral orbitofrontal volume. **(C)** Left paracentral volume. **(D)** Right paracentral volume. **(E)** Left transverse temporal volume. **(F)** Right posterior cingulate volume. **(G)** Left parahippocampal volume.

**FIGURE 2:**
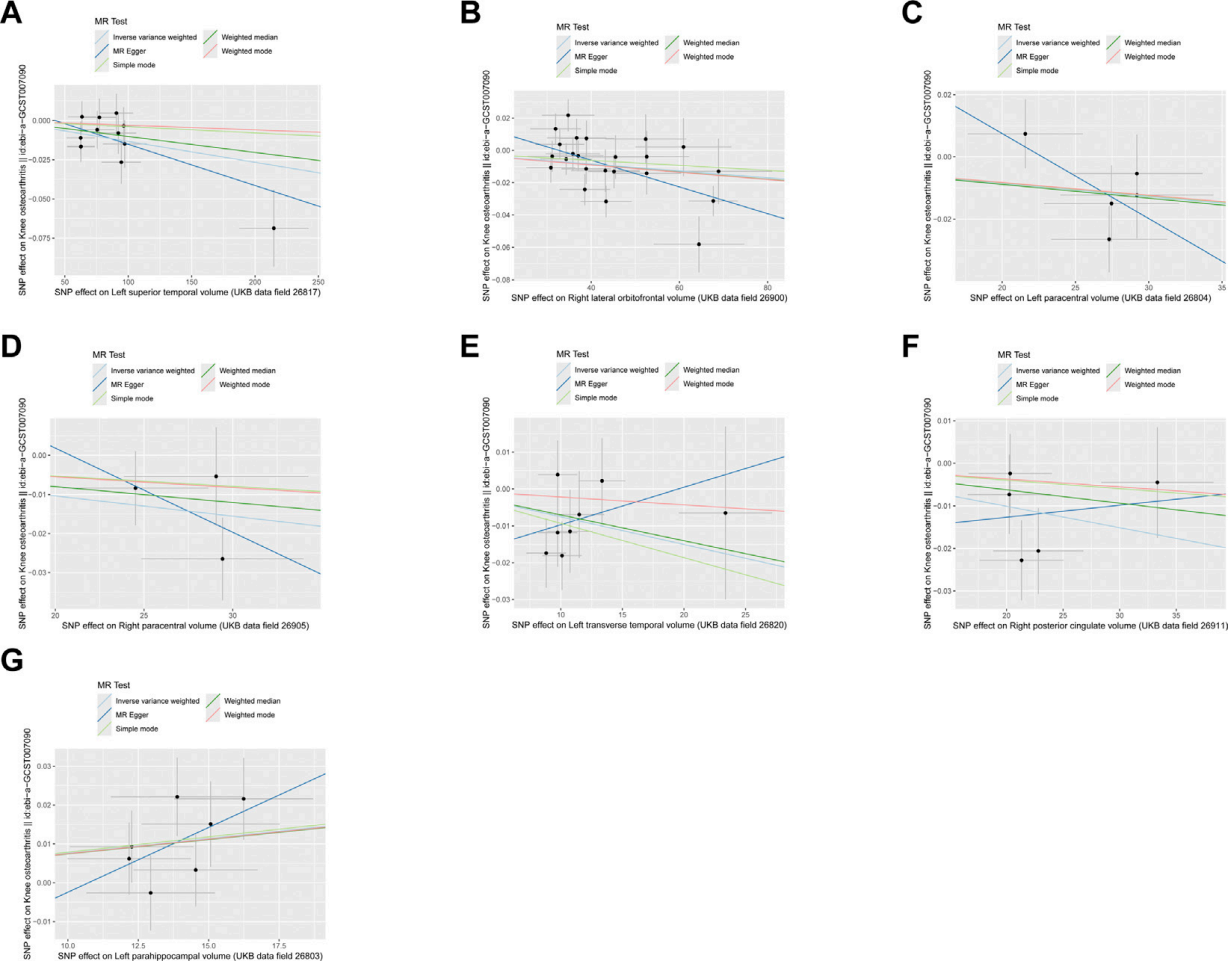
The scatter plot of MR results. **(A)** Left superior temporal volume. **(B)** Right lateral orbitofrontal volume. **(C)** Left paracentral volume. **(D)** Right paracentral volume. **(E)** Left transverse temporal volume. **(F)** Right posterior cingulate volume. **(G)** Left parahippocampal volume.

**FIGURE 3:**
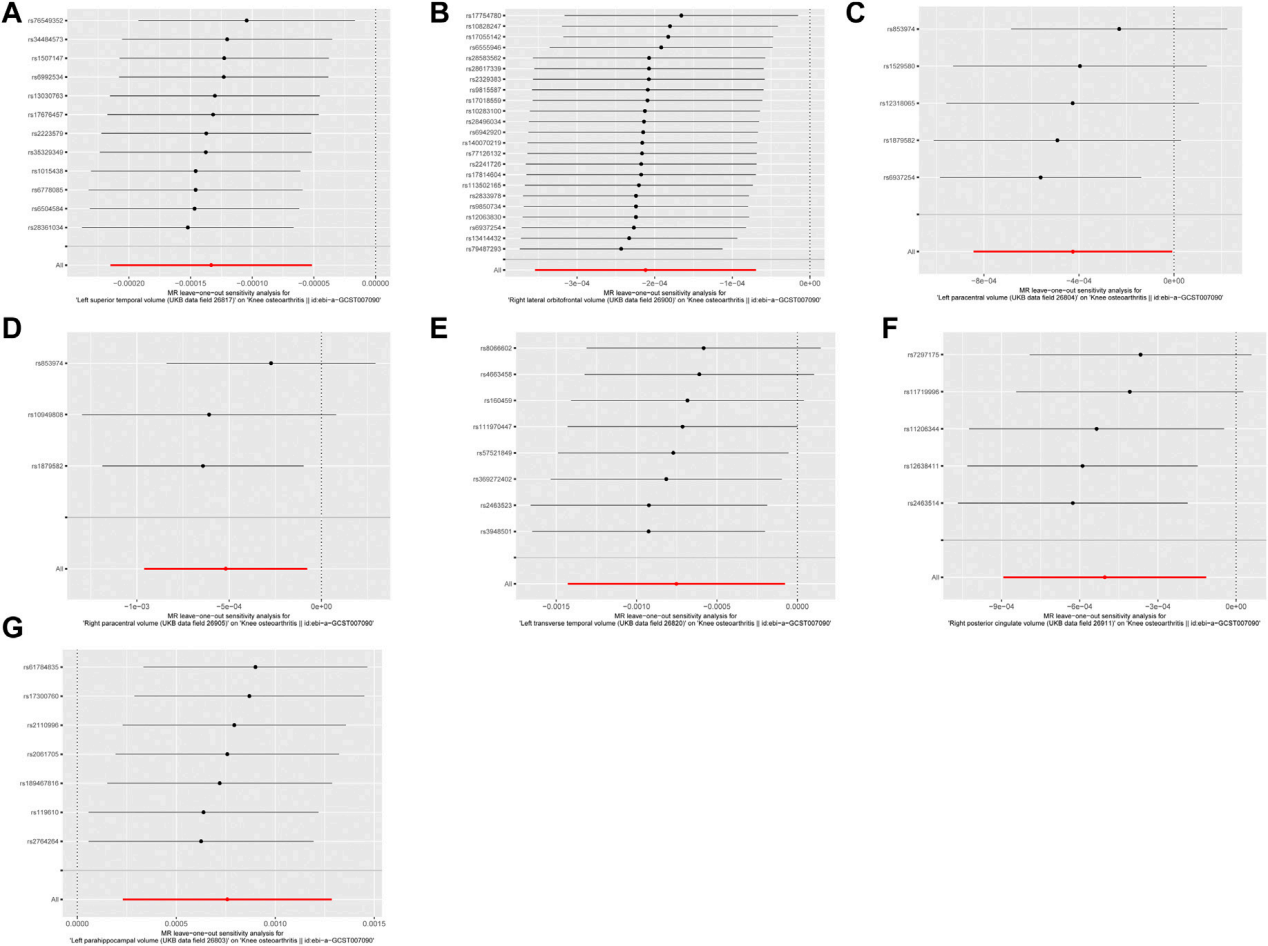
The leave-one-out analysis of MR results. **(A)** Left superior temporal volume. **(B)** Right lateral orbitofrontal volume. **(C)** Left paracentral volume. **(D)** Right paracentral volume. **(E)** Left transverse temporal volume. **(F)** Right posterior cingulate volume. **(G)** Left parahippocampal volume.

As depicted in Figures 1, 2, seven brain-wide volumes, including Left superior temporal volume (UKB data field 26,817) (beta = −1.33E-04, se = 4.17E-05, pval = 1.39E-03), Right lateral orbitofrontal volume (UKB data field 26,900) (beta = −2.12E-04, se = 7.26E-05, pval = 3.54E-03), Left parahippocampal volume (UKB data field 26,803) (beta = 7.58E-04, se = 2.69E-04, pval = 4.89E-03), Right posterior cingulate volume (UKB data field 26,911) (beta = −5.04E-04, se = 1.99E-04, pval = 1.12E-02), Right paracentral volume (UKB data field 26,905) (beta = −5.19E-04, se = 2.25E-04, pval = 2.14E-02), Left transverse temporal volume (UKB data field 26,820) (beta = −7.52E-04, se = 3.45E-04, pval = 2.91E-02), Left paracentral volume (UKB data field 26,804) (beta = −4.26E-04, se = 2.13E-04, pval = 4.61E-02). Increasing Left parahippocampal volume and Left caudal anterior cingulate volume is associated with an elevated risk of KOA, while an increase in Right posterior cingulate volume, Left transverse temporal volume, Right paracentral volume, Left paracentral volume, Right lateral orbitofrontal volume, and Left superior temporal volume is associated with a decreased risk of KOA. The analysis results for all phenotypes are listed in Supplementary Table S3. These findings remain consistent across heterogeneity and pleiotropy tests (as indicated in Supplementary Tables S4, S5). The leave-one-out analysis illustrating the association is depicted in Figure 3. The leave-one-out analysis reveals that, within these seven analyses involving two or more IVs, excluding any SNP doesn't affect the results, indicating the robustness of the findings.

The observed associations between specific brain volumes and KOA risk can be understood through the roles these brain regions play in pain perception, emotional processing, and motor control. For example, the parahippocampal gyrus is involved in memory and emotional responses, potentially influencing how individuals perceive and react to chronic pain, which could increase KOA risk. Conversely, regions such as the posterior cingulate and orbitofrontal cortex are implicated in pain modulation and emotional regulation, where increased volumes might enhance pain tolerance and coping mechanisms, thereby decreasing KOA risk. Variations in motor-related regions like the paracentral lobule could impact gait and balance, affecting joint stress and potentially modifying KOA progression. Understanding these neuroanatomical functions helps elucidate why changes in these brain volumes might correlate with differing KOA risks.

## Discussion

In this study, we systematically explored the causal relationships between brain-wide morphometric phenotypes and KOA using a two-sample Mendelian Randomization (TSMR) approach with GWAS data from the UK Biobank. By leveraging genetic epidemiology methods, we identified significant causal associations between KOA and specific brain morphometries, including volumes of the Left parahippocampal, Right posterior cingulate, Left transverse temporal, Left caudal anterior cingulate, Right paracentral, Left paracentral, and Right lateral orbitofrontal regions. These findings not only elucidate the neuropsychological contributions to KOA but also underscore the potential of neuroanatomical variations in influencing the risk and development of this debilitating condition. Our results remain robust across various analyses for heterogeneity and pleiotropy.

Brain-wide volumes, or the comprehensive volumetric measures of different regions within the human brain, play pivotal roles in various physiological processes and are crucial for understanding the complex interplay between brain structure and function (Hasan et al., 2011; Immonen et al., 2020). These volumetric attributes of the brain are not only indicative of individual neuroanatomical variability but also provide invaluable insights into the underlying mechanisms of numerous diseases and disorders, particularly those with neuropsychological components (Varghese et al., 2017; He et al., 2022; Statsenko et al., 2022; Díez-Cirarda et al., 2023). The significance of brain-wide volumes extends across multiple dimensions of human health and disease. For instance, variations in specific brain regions have been linked to a range of neuropsychiatric conditions, including schizophrenia, bipolar disorder, and major depressive disorder, where alterations in volumes of regions such as the hippocampus, amygdala, and prefrontal cortex are associated with the pathophysiology of these conditions (Ahmed et al., 2021; Grehl et al., 2023; Zovetti et al., 2023). Similarly, neurodegenerative diseases like Alzheimer’s and Parkinson’s disease show characteristic patterns of brain atrophy, making volumetric analysis a key tool in their diagnosis and progression monitoring (Horsager et al., 2020; Mathew et al., 2023). Beyond the realm of neuropsychiatric and neurodegenerative disorders, the significance of brain-wide volumes is increasingly recognized in the study of diseases traditionally not associated with the central nervous system. For example, recent studies, including our own investigation into KOA, highlight how variations in brain morphology can influence or reflect the susceptibility and progression of systemic diseases (Hari et al., 2024; Kim et al., 2024; Li et al., 2024; Lomer et al., 2024). This suggests a far-reaching influence of brain structure on overall health, possibly mediated through neuroendocrine and neuroimmune pathways that link brain function with peripheral organ systems.

The findings from our study suggest a nuanced relationship where increases in certain brain volumes are linked to either elevated or reduced risks of KOA, reflecting the intricate interplay between neuroanatomical structures and peripheral disease manifestations. Here, we delve into potential mechanisms through which these eight brain regions might influence KOA risk. The parahippocampal region plays a critical role in memory encoding and retrieval, which is essential for spatial navigation and pain memory (Deng et al., 2021). An increase in left parahippocampal volume might influence the individual’s pain perception and memory related to joint pain, potentially exacerbating the perceived severity of KOA symptoms. Similarly, the caudal anterior cingulate cortex (ACC) is known for its involvement in emotional processing, pain perception, and regulation (Wen et al., 2022). An increase in left caudal anterior cingulate volume could lead to heightened pain sensitivity or altered emotional responses to pain, contributing to the increased risk of KOA by influencing pain perception pathways. Conversely, the volumes of the right posterior cingulate, left transverse temporal, right and left paracentral, right lateral orbitofrontal, and left superior temporal regions are associated with a decreased risk of KOA, suggesting protective or compensatory effects. The posterior cingulate cortex is involved in the default mode network, playing a role in internally directed thought and memory (Wang et al., 2020). An increase in its volume could enhance cognitive resilience against chronic pain, potentially mitigating pain perception or the psychological impact of KOA. This region, part of the primary auditory cortex, could influence the risk of KOA through indirect pathways, perhaps by affecting communication and social interaction abilities that might influence physical activity levels or pain reporting tendencies. The paracentral lobule, part of the primary motor and sensory cortices, plays a role in processing and integrating sensory information from the body (Kong et al., 2022; Zhu et al., 2022). Increased volume in these areas might enhance sensory discrimination, potentially contributing to more effective management or perception of osteoarthritic pain. The lateral orbitofrontal cortex is crucial for decision-making and reward evaluation (Paunova et al., 2022). A larger volume could be associated with better coping strategies, emotional regulation, and decision-making processes concerning health behaviors that mitigate KOA risk. The superior temporal gyrus is involved in auditory processing and language comprehension (Zhu et al., 2019). While its direct role in influencing KOA risk is less intuitive, it may be related to social cognition and communication abilities, indirectly affecting health behaviors and pain perception. The differential impact of these brain volumes on KOA risk highlights the importance of neurocognitive and neuropsychological factors in the development and experience of osteoarthritis. It suggests that the brain’s role extends beyond mere pain processing, potentially influencing behaviors, emotional responses, and coping mechanisms that collectively modulate disease risk and progression. Further research into these associations will be crucial for developing comprehensive models of KOA pathogenesis, incorporating both neuroanatomical and psychosocial factors.

The implications of our findings are significant for both future research and potential clinical applications. By identifying specific brain-wide volumes that are causally associated with KOA, this study opens new avenues for exploring the neurobiological mechanisms underlying the disease. Future research should focus on elucidating the precise pathways through which these neuroanatomical variations influence KOA, potentially incorporating advanced neuroimaging techniques and longitudinal studies to monitor changes over time. Additionally, these insights pave the way for the development of targeted interventions that address the neuropsychological aspects of KOA. Clinically, understanding the brain’s role in KOA pathogenesis could lead to innovative treatment strategies that go beyond symptom management to modify disease progression. For instance, interventions aimed at modulating brain structures or functions, such as cognitive-behavioral therapy, neuromodulation, or pharmacological approaches targeting specific brain regions, could be explored as complementary treatments for KOA.

Potential sources of bias in two-sample Mendelian Randomization studies include pleiotropy, population stratification, weak instrument bias, and measurement error. Pleiotropy occurs when genetic variants influence multiple traits, potentially confounding the causal estimates. To address this, we employed the MR Egger method, which allows for an intercept term to account for pleiotropy, and also used the weighted median and mode-based methods to provide robust causal estimates even if some instruments are invalid. Population stratification, which can arise from genetic differences across subpopulations, was minimized by restricting our analysis to unrelated White European participants from the UK Biobank. Weak instrument bias, where genetic variants do not strongly associate with the exposure, was mitigated by selecting SNPs with genome-wide significance (P < 5E-8) and using the IVW method to assign more weight to precisely estimated effects. Measurement error in exposure or outcome data can distort causal estimates; hence, we used high-quality GWAS data and conducted sensitivity analyses, including the leave-one-out method, to ensure the robustness of our findings. These steps collectively enhance the reliability and validity of our conclusions regarding the causal relationships between brain-wide morphometric variations and KOA.

It is important to acknowledge several limitations that highlight areas for future research. Firstly, the analysis was conducted within a predominantly White European population from the UK Biobank, which may limit the generalizability of our findings to other ethnicities and populations. The genetic, environmental, and lifestyle factors influencing brain morphometry and KOA risk can vary significantly across different populations, necessitating further studies in diverse cohorts to validate and extend our findings. Secondly, our study relied on MR, a method that assumes the selected IVs are strongly associated with the exposure (brain volumes) but not with confounders of the exposure-outcome relationship. While we rigorously selected IVs based on stringent criteria, the potential for residual confounding cannot be entirely excluded. Unmeasured confounders, such as socioeconomic status, lifestyle factors, and other health conditions, could influence both brain morphometry and KOA risk, potentially biasing our causal estimates. Furthermore, our analysis focused on 83 cortical and subcortical gray-matter volumes, leaving out other aspects of brain anatomy, such as white matter integrity, functional connectivity, and cortical thickness, which could also play roles in KOA. Future studies incorporating these additional brain metrics might uncover more comprehensive insights into the neuropsychological contributions to KOA.

In conclusion, while this study contributes some insights into the potential neuropsychological underpinnings of KOA, several limitations underscore the need for further research. Future studies should incorporate diverse populations to ensure the generalizability of our findings across different ethnicities and environments. Additionally, expanding the scope to include other brain metrics, such as white matter integrity, functional connectivity, and cortical thickness, will provide a more comprehensive understanding of the neurobiological mechanisms linking brain structure to KOA. Longitudinal designs are also essential to establish temporal relationships and causality. Addressing these areas in future research will enhance our knowledge and facilitate the translation of these findings into clinical practice, potentially leading to more effective interventions for KOA.

## Data Availability

The original contributions presented in the study are included in the article/Supplementary Material, further inquiries can be directed to the corresponding authors.
