# Supplementary material for: The causal relationship between human brain morphometry and knee osteoarthritis: a two-sample Mendelian randomization study
Source: Front Genet. 2024 Jul 8;15:1420134. doi: 10.3389/fgene.2024.1420134 (PMC11260717; doi:10.3389/fgene.2024.1420134)
Supplement: Supplementary file 1 [file DataSheet1.PDF]

Table S1

accessionId of ebi	reportedTrait	efoTraits	discoverySampleAncestry	pubmedId
GCST90085819	Brain stem volume (UKB data field 26526)	brain stem volume measurement	36778 European	36987996
GCST90085820	Left accumbens area volume (UKB data field 26564)	nucleus accumbens volume	36778 European	36987996
GCST90085821	Left amygdala volume (UKB data field 26563)	amygdala volume	36778 European	36987996
GCST90085822	Left banks of superior temporal sulcus volume (UKB data field 26789)	banks of the superior temporal sulcus volume measurement	36778 European	36987996
GCST90085823	Left caudal anterior cingulate volume (UKB data field 26790)	caudal anterior cingulate cortex volume measurement	36778 European	36987996
GCST90085824	Left caudal middle frontal volume (UKB data field 26791)	caudal middle frontal gyrus volume measurement	36778 European	36987996
GCST90085825	Left caudate volume (UKB data field 26559)	caudate nucleus volume	36778 European	36987996
GCST90085867	Right cuneus volume (UKB data field 26893)	cuneus cortex volume measurement	36778 European	36987996
GCST90085826	Left cuneus volume (UKB data field 26792)	cuneus cortex volume measurement	36778 European	36987996
GCST90085827	Left ventral diencephalon (DC) volume (UKB data field 26565)	ventral diencephalon volume measurement	36778 European	36987996
GCST90085828	Left entorhinal volume (UKB data field 26793)	entorhinal cortex volume measurement	36778 European	36987996
GCST90085829	Left frontal pole volume (UKB data field 26819)	frontal pole volume measurement	36778 European	36987996
GCST90085830	Left fusiform volume (UKB data field 26794)	fusiform gyrus volume measurement	36778 European	36987996
GCST90085831	Left hippocampus volume (UKB data field 26562)	hippocampal volume	36778 European	36987996
GCST90085832	Left inferior parietal volume (UKB data field 26795)	inferior parietal cortex volume measurement	36778 European	36987996
GCST90085833	Left inferior temporal volume (UKB data field 26796)	inferior temporal gyrus volume measurement	36778 European	36987996
GCST90085834	Left insula volume (UKB data field 26821)	insular cortex volume measurement	36778 European	36987996
GCST90085835	Left isthmus cingulate volume (UKB data field 26797)	isthmus cingulate cortex volume measurement	36778 European	36987996
GCST90085836	Left lateral occipital volume (UKB data field 26798)	lateral occipital cortex volume measurement	36778 European	36987996
GCST90085837	Left lateral orbitofrontal volume (UKB data field 26799)	lateral orbital frontal cortex volume measurement	36778 European	36987996
GCST90085838	Left lingual volume (UKB data field 26800)	lingual gyrus volume measurement	36778 European	36987996
GCST90085839	Left medial orbitofrontal volume (UKB data field 26801)	medial orbital frontal cortex volume measurement	36778 European	36987996
GCST90085840	Left middle temporal volume (UKB data field 26802)	middle temporal gyrus volume measurement	36778 European	36987996
GCST90085841	Left pallidum volume (UKB data field 26561)	pallidum volume	36778 European	36987996
GCST90085842	Left paracentral volume (UKB data field 26804)	paracentral lobule volume measurement	36778 European	36987996
GCST90085843	Left parahippocampal volume (UKB data field 26803)	parahippocampal gyrus volume measurement	36778 European	36987996
GCST90085844	Left pars opercularis volume (UKB data field 26805)	pars opercularis volume measurement	36778 European	36987996
GCST90085845	Left pars orbitalis volume (UKB data field 26806)	pars orbitalis volume measurement	36778 European	36987996
GCST90085846	Left pars triangularis volume (UKB data field 26807)	pars triangularis volume measurement	36778 European	36987996
GCST90085847	Left pericalcarine volume (UKB data field 26808)	pericalcarine cortex volume measurement	36778 European	36987996
GCST90085848	Left postcentral volume (UKB data field 26809)	postcentral gyrus volume measurement	36778 European	36987996
GCST90085849	Left posterior cingulate volume (UKB data field 26810)	posterior cingulate cortex volume measurement	36778 European	36987996
GCST90085850	Left precentral volume (UKB data field 26811)	precentral gyrus volume measurement	36778 European	36987996
GCST90085851	Left precuneus volume (UKB data field 26812)	precuneus cortex volume measurement	36778 European	36987996
GCST90085852	Left putamen volume (UKB data field 26560)	putamen volume	36778 European	36987996
GCST90085853	Left rostral anterior cingulate volume (UKB data field 26813)	rostral anterior cingulate cortex volume measurement	36778 European	36987996
GCST90085854	Left rostral middle frontal volume (UKB data field 26814)	rostral middle frontal gyrus volume measurement	36778 European	36987996
GCST90085855	Left superior frontal volume (UKB data field 26815)	superior frontal gyrus volume measurement	36778 European	36987996
GCST90085856	Left superior parietal volume (UKB data field 26816)	superior parietal cortex volume measurement	36778 European	36987996
GCST90085857	Left superior temporal volume (UKB data field 26817)	superior temporal gyrus volume measurement	36778 European	36987996
GCST90085858	Left supramarginal volume (UKB data field 26818)	supramarginal gyrus volume measurement	36778 European	36987996
GCST90085859	Left thalamus proper volume (UKB data field 26558)	thalamus volume	36778 European	36987996
GCST90085860	Left transverse temporal volume (UKB data field 26820)	transverse temporal cortex volume measurement	36778 European	36987996
GCST90085861	Right accumbens area volume (UKB data field 26595)	nucleus accumbens volume	36778 European	36987996
GCST90085862	Right amygdala volume (UKB data field 26594)	amygdala volume	36778 European	36987996
GCST90085863	Right banks of superior temporal sulcus volume (UKB data field 26890)	banks of the superior temporal sulcus volume measurement	36778 European	36987996
GCST90085864	Right caudal anterior cingulate volume (UKB data field 26891)	caudal anterior cingulate cortex volume measurement	36778 European	36987996
GCST90085865	Right caudal middle frontal volume (UKB data field 26892)	caudal middle frontal gyrus volume measurement	36778 European	36987996
GCST90085866	Right caudate volume (UKB data field 26590)	caudate nucleus volume	36778 European	36987996
GCST90085868	Right ventral diencephalon (DC) volume (UKB data field 26596)	ventral diencephalon volume measurement	36778 European	36987996
GCST90085869	Right entorhinal volume (UKB data field 26894)	entorhinal cortex volume measurement	36778 European	36987996
GCST90085870	Right frontal pole volume (UKB data field 26920)	frontal pole volume measurement	36778 European	36987996
GCST90085871	Right fusiform volume (UKB data field 26895)	fusiform gyrus volume measurement	36778 European	36987996
GCST90085872	Right hippocampus volume (UKB data field 26593)	hippocampal volume	36778 European	36987996
GCST90085873	Right inferior parietal volume (UKB data field 26896)	inferior parietal cortex volume measurement	36778 European	36987996
GCST90085874	Right inferior temporal volume (UKB data field 26897)	inferior temporal gyrus volume measurement	36778 European	36987996
GCST90085875	Right insula volume (UKB data field 26922)	insular cortex volume measurement	36778 European	36987996
GCST90085876	Right isthmus cingulate volume (UKB data field 26898)	isthmus cingulate cortex volume measurement	36778 European	36987996
GCST90085877	Right lateral occipital volume (UKB data field 26899)	lateral occipital cortex volume measurement	36778 European	36987996
GCST90085878	Right lateral orbitofrontal volume (UKB data field 26900)	lateral orbital frontal cortex volume measurement	36778 European	36987996
GCST90085879	Right lingual volume (UKB data field 26901)	lingual gyrus volume measurement	36778 European	36987996
GCST90085880	Right medial orbitofrontal volume (UKB data field 26902)	medial orbital frontal cortex volume measurement	36778 European	36987996
GCST90085881	Right middle temporal volume (UKB data field 26903)	middle temporal gyrus volume measurement	36778 European	36987996
GCST90085882	Right pallidum volume (UKB data field 26592)	pallidum volume	36778 European	36987996
GCST90085883	Right paracentral volume (UKB data field 26905)	paracentral lobule volume measurement	36778 European	36987996
GCST90085884	Right parahippocampal volume (UKB data field 26904)	parahippocampal gyrus volume measurement	36778 European	36987996
GCST90085885	Right pars opercularis volume (UKB data field 26906)	pars opercularis volume measurement	36778 European	36987996
GCST90085886	Right pars orbitalis volume (UKB data field 26907)	pars orbitalis volume measurement	36778 European	36987996
GCST90085887	Right pars triangularis volume (UKB data field 26908)	pars triangularis volume measurement	36778 European	36987996
GCST90085888	Right pericalcarine volume (UKB data field 26909)	pericalcarine cortex volume measurement	36778 European	36987996
GCST90085889	Right postcentral volume (UKB data field 26910)	postcentral gyrus volume measurement	36778 European	36987996
GCST90085890	Right posterior cingulate volume (UKB data field 26911)	posterior cingulate cortex volume measurement	36778 European	36987996
GCST90085891	Right precentral volume (UKB data field 26912)	precentral gyrus volume measurement	36778 European	36987996
GCST90085892	Right precuneus volume (UKB data field 26913)	precuneus cortex volume measurement	36778 European	36987996
GCST90085893	Right putamen volume (UKB data field 26591)	putamen volume	36778 European	36987996
GCST90085894	Right rostral anterior cingulate volume (UKB data field 26914)	rostral anterior cingulate cortex volume measurement	36778 European	36987996
GCST90085895	Right rostral middle frontal volume (UKB data field 26915)	rostral middle frontal gyrus volume measurement	36778 European	36987996
GCST90085896	Right superior frontal volume (UKB data field 26916)	superior frontal gyrus volume measurement	36778 European	36987996
GCST90085897	Right superior parietal volume (UKB data field 26917)	superior parietal cortex volume measurement	36778 European	36987996
GCST90085898	Right superior temporal volume (UKB data field 26918)	superior temporal gyrus volume measurement	36778 European	36987996
GCST90085899	Right supramarginal volume (UKB data field 26919)	supramarginal gyrus volume measurement	36778 European	36987996
GCST90085900	Right thalamus proper volume (UKB data field 26589)	thalamus volume	36778 European	36987996
GCST90085901	Right transverse temporal volume (UKB data field 26921)	transverse temporal cortex volume measurement	36778 European	36987996

Table S2

SNP	effect_allele.exposure	other_allele.exposure	beta.exposure	beta.outcome	eaf.exposure	eaf.outcome	chr	pos	se.outcome	samplesize.outcome	pval.outcome	outcome	pval.exposure	se.exposure	exposure
rs10029738	C	A	27.7851	-0.0093	0.287087	0.289	4	145297419	0.0102	403124	0.3628	Knee osteoarthritis || id:ebi-a-GCST007090	2.57E-11	4.16614	Left cuneus volume (UKB data field 26792)
rs10761135	T	C	-23.0261	0.0081	0.672644	0.6774	9	94647024	0.0099	403124	0.4118	Knee osteoarthritis || id:ebi-a-GCST007090	9.82E-09	4.01579	Left cuneus volume (UKB data field 26792)
rs11022131	C	G	33.6212	0.0029	0.750134	0.7513	11	12072213	0.0107	403124	0.7865	Knee osteoarthritis || id:ebi-a-GCST007090	1.00E-14	4.34459	Left cuneus volume (UKB data field 26792)
rs11918598	C	T	28.6197	0.0067	0.83139	0.8312	3	119024937	0.0124	403124	0.5905	Knee osteoarthritis || id:ebi-a-GCST007090	1.27E-08	5.02987	Left cuneus volume (UKB data field 26792)
rs12273384	G	A	35.5357	-0.0171	0.861048	0.8598	11	133936487	0.0135	403124	0.2048	Knee osteoarthritis || id:ebi-a-GCST007090	9.39E-11	5.48695	Left cuneus volume (UKB data field 26792)
rs12508031	A	T	-26.2369	-0.0184	0.656114	0.6546	4	19073685	0.0101	403124	0.0683597	Knee osteoarthritis || id:ebi-a-GCST007090	2.09E-10	4.12885	Left cuneus volume (UKB data field 26792)
rs12700002	T	C	-32.2188	-0.0057	0.605669	0.604	7	18905450	0.0095	403124	0.5465	Knee osteoarthritis || id:ebi-a-GCST007090	6.92E-17	3.85924	Left cuneus volume (UKB data field 26792)
rs13082262	G	A	23.2081	-0.0033	0.38293	0.3834	3	141652082	0.0096	403124	0.7326	Knee osteoarthritis || id:ebi-a-GCST007090	2.54E-09	3.89484	Left cuneus volume (UKB data field 26792)
rs1635535	A	G	-38.6994	0.0581	0.147107	0.146	12	48386241	0.0132	403124	1.00E-05	Knee osteoarthritis || id:ebi-a-GCST007090	3.79E-13	5.32847	Left cuneus volume (UKB data field 26792)
rs2164950	G	A	103.059	-0.0101	0.877264	0.8748	14	59627631	0.014	403124	0.4725	Knee osteoarthritis || id:ebi-a-GCST007090	5.14E-72	5.74269	Left cuneus volume (UKB data field 26792)
rs2279829	C	T	-26.4103	0.0167	0.78147	0.7854	3	147106319	0.0113	403124	0.1405	Knee osteoarthritis || id:ebi-a-GCST007090	7.18E-09	4.56399	Left cuneus volume (UKB data field 26792)
rs2836466	A	C	-21.7657	0.0074	0.583509	0.5828	21	39896856	0.0094	403124	0.4344	Knee osteoarthritis || id:ebi-a-GCST007090	1.22E-08	3.82091	Left cuneus volume (UKB data field 26792)
rs3012434	A	G	-28.0137	0.0088	0.190639	0.1893	6	170545279	0.012	403124	0.4665	Knee osteoarthritis || id:ebi-a-GCST007090	9.66E-09	4.88333	Left cuneus volume (UKB data field 26792)
rs4437022	C	A	-24.4968	-0.0205	0.554564	0.5513	20	52432451	0.0094	403124	0.0284597	Knee osteoarthritis || id:ebi-a-GCST007090	1.10E-10	3.79679	Left cuneus volume (UKB data field 26792)
rs4792858	A	C	23.2462	-0.0155	0.694722	0.6943	17	43244700	0.0101	403124	0.1259	Knee osteoarthritis || id:ebi-a-GCST007090	1.49E-08	4.10496	Left cuneus volume (UKB data field 26792)
rs4973930	A	C	-23.1038	-0.0235	0.475222	0.4741	3	41306063	0.0093	403124	0.0115401	Knee osteoarthritis || id:ebi-a-GCST007090	1.05E-09	3.78621	Left cuneus volume (UKB data field 26792)
rs61760711	T	A	24.4679	3.00E-04	0.626537	0.6301	1	18952904	0.0097	403124	0.9749	Knee osteoarthritis || id:ebi-a-GCST007090	4.58E-10	3.92555	Left cuneus volume (UKB data field 26792)
rs651858	C	T	-26.5761	-0.0059	0.352048	0.3524	8	108992158	0.0097	403124	0.543	Knee osteoarthritis || id:ebi-a-GCST007090	1.57E-11	3.94214	Left cuneus volume (UKB data field 26792)
rs6563121	T	C	24.4782	0.0178	0.480339	0.477	13	80175000	0.0093	403124	0.0549604	Knee osteoarthritis || id:ebi-a-GCST007090	7.47E-11	3.75961	Left cuneus volume (UKB data field 26792)
rs6701689	T	C	-26.32	-0.0146	0.722009	0.7251	1	2020343	0.0104	403124	0.159	Knee osteoarthritis || id:ebi-a-GCST007090	4.27E-10	4.21543	Left cuneus volume (UKB data field 26792)
rs7214750	C	T	45.3905	0.0058	0.929816	0.9309	17	63903119	0.0185	403124	0.752801	Knee osteoarthritis || id:ebi-a-GCST007090	1.10E-09	7.44828	Left cuneus volume (UKB data field 26792)
rs72685500	A	T	23.3417	0.0117	0.700669	0.7002	4	157090189	0.0102	403124	0.2525	Knee osteoarthritis || id:ebi-a-GCST007090	1.62E-08	4.13277	Left cuneus volume (UKB data field 26792)
rs7295050	A	G	-25.2916	0.005	0.719601	0.7212	12	130578130	0.0103	403124	0.6321	Knee osteoarthritis || id:ebi-a-GCST007090	1.82E-09	4.20635	Left cuneus volume (UKB data field 26792)
rs74283894	G	A	37.0203	-0.0022	0.701449	0.7043	3	104721473	0.0107	403124	0.8403	Knee osteoarthritis || id:ebi-a-GCST007090	2.20E-19	4.11215	Left cuneus volume (UKB data field 26792)
rs7442779	A	G	-52.0802	0.0014	0.949834	0.9518	5	92788278	0.0217	403124	0.9473	Knee osteoarthritis || id:ebi-a-GCST007090	1.36E-09	8.59314	Left cuneus volume (UKB data field 26792)
rs79636188	T	C	62.7772	-0.0111	0.959221	0.9579	6	127078181	0.0232	403124	0.632	Knee osteoarthritis || id:ebi-a-GCST007090	3.97E-11	9.50411	Left cuneus volume (UKB data field 26792)
rs910697	A	G	24.3727	-0.0028	0.541999	0.5379	1	113063125	0.0093	403124	0.766699	Knee osteoarthritis || id:ebi-a-GCST007090	1.15E-10	3.78143	Left cuneus volume (UKB data field 26792)
rs9323580	A	G	22.5652	9.00E-04	0.698825	0.6986	14	25443740	0.0101	403124	0.9263	Knee osteoarthritis || id:ebi-a-GCST007090	3.98E-08	4.10884	Left cuneus volume (UKB data field 26792)
rs9935548	G	A	24.4947	-0.0021	0.63568	0.6328	16	49540235	0.0096	403124	0.825	Knee osteoarthritis || id:ebi-a-GCST007090	3.30E-10	3.89796	Left cuneus volume (UKB data field 26792)
rs10034377	G	T	15.7897	-0.0121	0.345413	0.3439	4	4850071	0.0098	403124	0.2165	Knee osteoarthritis || id:ebi-a-GCST007090	4.03E-08	2.87637	Right isthmus cingulate volume (UKB data field 26898)
rs10217651	A	G	-15.6941	-0.0141	0.389165	0.3847	9	118923652	0.0096	403124	0.1398	Knee osteoarthritis || id:ebi-a-GCST007090	3.06E-08	2.83381	Right isthmus cingulate volume (UKB data field 26898)
rs1045279	C	T	22.5408	-0.0158	0.151029	0.15	3	141663683	0.013	403124	0.2259	Knee osteoarthritis || id:ebi-a-GCST007090	5.78E-09	3.87093	Right isthmus cingulate volume (UKB data field 26898)
rs2022464	A	C	-16.3403	-0.0223	0.299406	0.2945	6	108945370	0.0102	403124	0.0286702	Knee osteoarthritis || id:ebi-a-GCST007090	4.62E-08	2.98972	Right isthmus cingulate volume (UKB data field 26898)
rs2252032	T	C	15.6326	0.0136	0.466323	0.4678	2	37099339	0.0093	403124	0.1441	Knee osteoarthritis || id:ebi-a-GCST007090	1.59E-08	2.766	Right isthmus cingulate volume (UKB data field 26898)
rs2336713	G	T	16.0207	-0.0085	0.361271	0.3649	12	65797096	0.0096	403124	0.3762	Knee osteoarthritis || id:ebi-a-GCST007090	2.52E-08	2.8753	Right isthmus cingulate volume (UKB data field 26898)
rs2942003	T	G	26.1175	0.0107	0.363617	0.3635	17	44576704	0.0108	403124	0.3198	Knee osteoarthritis || id:ebi-a-GCST007090	6.39E-16	3.23173	Right isthmus cingulate volume (UKB data field 26898)
rs61878823	C	T	18.7668	0.0116	0.774691	0.773	11	12969166	0.0119	403124	0.3288	Knee osteoarthritis || id:ebi-a-GCST007090	2.24E-08	3.35578	Right isthmus cingulate volume (UKB data field 26898)
rs10035673	T	A	-27.588	0.0253	0.474008	0.4772	5	170776747	0.0094	403124	0.00682905	Knee osteoarthritis || id:ebi-a-GCST007090	3.17E-08	4.98729	Left insula volume (UKB data field 26821)
rs1015521	G	T	-30.2657	0.0025	0.667386	0.6708	4	106115450	0.0099	403124	0.7991	Knee osteoarthritis || id:ebi-a-GCST007090	6.98E-09	5.22596	Left insula volume (UKB data field 26821)
rs10283100	A	G	-86.7051	0.013	0.0552912	0.0554	8	120596023	0.0203	403124	0.5221	Knee osteoarthritis || id:ebi-a-GCST007090	1.13E-15	10.8214	Left insula volume (UKB data field 26821)
rs10817103	C	T	-45.6885	-0.0055	0.886191	0.8871	9	113649502	0.0148	403124	0.711001	Knee osteoarthritis || id:ebi-a-GCST007090	5.51E-09	7.83567	Left insula volume (UKB data field 26821)
rs11079849	C	T	-35.8523	0.0239	0.666227	0.672	17	47090785	0.0099	403124	0.0156099	Knee osteoarthritis || id:ebi-a-GCST007090	8.61E-12	5.25071	Left insula volume (UKB data field 26821)
rs1163564	C	T	34.6178	0.0068	0.209399	0.2104	10	104694036	0.0122	403124	0.5776	Knee osteoarthritis || id:ebi-a-GCST007090	2.02E-08	6.16997	Left insula volume (UKB data field 26821)
rs13041542	G	A	-31.5021	0.0066	0.656365	0.6537	20	3139383	0.0099	403124	0.5076	Knee osteoarthritis || id:ebi-a-GCST007090	2.24E-09	5.26817	Left insula volume (UKB data field 26821)
rs13069749	C	T	44.2871	-0.006	0.160071	0.1591	3	141737890	0.0134	403124	0.652501	Knee osteoarthritis || id:ebi-a-GCST007090	6.34E-11	6.7764	Left insula volume (UKB data field 26821)
rs146744443	A	G	-88.4918	-0.0174	0.967793	0.9667	7	156168749	0.0259	403124	0.5028	Knee osteoarthritis || id:ebi-a-GCST007090	2.55E-10	13.9931	Left insula volume (UKB data field 26821)
rs147505315	A	G	-34.5872	0.0105	0.723813	0.7261	8	120675373	0.0111	403124	0.3442	Knee osteoarthritis || id:ebi-a-GCST007090	6.10E-10	5.58963	Left insula volume (UKB data field 26821)
rs1855349	G	T	28.8252	-0.0137	0.597823	0.5942	20	31357097	0.0095	403124	0.1484	Knee osteoarthritis || id:ebi-a-GCST007090	9.88E-09	5.02811	Left insula volume (UKB data field 26821)
rs2045258	A	G	-46.229	0.0315	0.549344	0.5482	6	126674354	0.0093	403124	0.000720908	Knee osteoarthritis || id:ebi-a-GCST007090	9.94E-21	4.95132	Left insula volume (UKB data field 26821)
rs309592	G	A	43.3457	-0.0053	0.201019	0.2	5	82848630	0.0116	403124	0.6488	Knee osteoarthritis || id:ebi-a-GCST007090	1.61E-12	6.13569	Left insula volume (UKB data field 26821)
rs321404	T	C	30.299	-0.0105	0.687473	0.6855	11	110949618	0.01	403124	0.2963	Knee osteoarthritis || id:ebi-a-GCST007090	1.45E-08	5.34589	Left insula volume (UKB data field 26821)
rs426632	C	T	29.2449	0.0178	0.598674	0.6037	9	118996950	0.0095	403124	0.0616396	Knee osteoarthritis || id:ebi-a-GCST007090	8.14E-09	5.07244	Left insula volume (UKB data field 26821)
rs61886103	G	T	62.8961	-0.0091	0.94246	0.9432	11	63606309	0.0201	403124	0.650401	Knee osteoarthritis || id:ebi-a-GCST007090	3.52E-09	10.6505	Left insula volume (UKB data field 26821)
rs6802667	C	G	-30.9948	0.0149	0.482674	0.4837	3	142000210	0.0094	403124	0.1136	Knee osteoarthritis || id:ebi-a-GCST007090	8.10E-10	5.04559	Left insula volume (UKB data field 26821)
rs6960056	G	A	-29.8103	-2.00E-04	0.566689	0.5698	7	92673154	0.0094	403124	0.9862	Knee osteoarthritis || id:ebi-a-GCST007090	2.41E-09	4.99555	Left insula volume (UKB data field 26821)
rs7306710	T	C	38.1327	0.0179	0.482978	0.4818	12	66376091	0.0093	403124	0.0555098	Knee osteoarthritis || id:ebi-a-GCST007090	2.19E-14	4.99181	Left insula volume (UKB data field 26821)
rs8080078	C	T	35.892	0.0425	0.496174	0.0148	17	28195773	0.0627	403124	0.4975	Knee osteoarthritis || id:ebi-a-GCST007090	4.19E-13	4.9512	Left insula volume (UKB data field 26821)
rs8080078	C	T	35.892	-0.0219	0.496174	0.4925	17	28195773	0.0093	403124	0.0182398	Knee osteoarthritis || id:ebi-a-GCST007090	4.19E-13	4.9512	Left insula volume (UKB data field 26821)
rs9607693	G	A	34.4692	3.00E-04	0.427803	0.4241	22	40566829	0.0101	403124	0.978	Knee osteoarthritis || id:ebi-a-GCST007090	1.09E-11	5.07364	Left insula volume (UKB data field 26821)
rs10057395	A	G	26.0155	-0.0098	0.471092	0.4703	5	60088794	0.0093	403124	0.2941	Knee osteoarthritis || id:ebi-a-GCST007090	8.16E-16	3.23101	Right pericalcarine volume (UKB data field 26909)
rs10237366	G	C	-20.9595	-7.00E-04	0.628509	0.6268	7	18915559	0.0097	403124	0.9464	Knee osteoarthritis || id:ebi-a-GCST007090	4.17E-10	3.3548	Right pericalcarine volume (UKB data field 26909)
rs11022129	T	C	27.8047	0.0032	0.759949	0.7608	11	12071436	0.011	403124	0.7703	Knee osteoarthritis || id:ebi-a-GCST007090	2.52E-13	3.79947	Right pericalcarine volume (UKB data field 26909)
rs11946517	G	C	19.7282	-0.0064	0.418943	0.4184	4	145599534	0.0094	403124	0.4969	Knee osteoarthritis || id:ebi-a-GCST007090	1.26E-09	3.24909	Right pericalcarine volume (UKB data field 26909)
rs12200595	G	T	22.4858	-0.0122	0.724663	0.7254	6	138862574	0.0104	403124	0.2429	Knee osteoarthritis || id:ebi-a-GCST007090	5.29E-10	3.62069	Right pericalcarine volume (UKB data field 26909)
rs1232186	C	T	20.9449	-0.0042	0.547486	0.55	11	31622534	0.0093	403124	0.649	Knee osteoarthritis || id:ebi-a-GCST007090	7.20E-11	3.21419	Right pericalcarine volume (UKB data field 26909)
rs12508031	A	T	-23.5388	-0.0184	0.656114	0.6546	4	19073685	0.0101	403124	0.0683597	Knee osteoarthritis || id:ebi-a-GCST007090	2.35E-11	3.52257	Right pericalcarine volume (UKB data field 26909)
rs12550461	T	A	32.9341	-0.0141	0.877868	0.8758	8	78091799	0.0141	403124	0.3189	Knee osteoarthritis || id:ebi-a-GCST007090	2.25E-11	4.9236	Right pericalcarine volume (UKB data field 26909)
rs12889303	G	A	-19.0121	-0.0366	0.679002	0.6798	14	104161666	0.0105	403124	0.000517798	Knee osteoarthritis || id:ebi-a-GCST007090	4.09E-08	3.46502	Right pericalcarine volume (UKB data field 26909)
rs13079190	A	G	19.333	-0.0085	0.370558	0.3679	3	13822543	0.0097	403124	0.3825	Knee osteoarthritis || id:ebi-a-GCST007090	8.62E-09	3.35882	Right pericalcarine volume (UKB data field 26909)
rs17185460	T	C	20.8462	0.0028	0.701611	0.7012	14	25429598	0.0102	403124	0.7824	Knee osteoarthritis || id:ebi-a-GCST007090	3.35E-09	3.52522	Right pericalcarine volume (UKB data field 26909)
rs17481712	A	G	-77.0308	-0.0112	0.985088	0.9851	1	88254265	0.0385	403124	0.7704	Knee osteoarthritis || id:ebi-a-GCST007090	8.68E-09	13.3858	Right pericalcarine volume (UKB data field 26909)
rs17543864	G	A	24.9112	-0.002	0.677575	0.6764	11	92556100	0.01	403124	0.8407	Knee osteoarthritis || id:ebi-a-GCST007090	8.75E-13	3.4846	Right pericalcarine volume (UKB data field 26909)
rs17788691	C	T	-23.5185	-0.01	0.746346	0.7448	16	49488151	0.0107	403124	0.3497	Knee osteoarthritis || id:ebi-a-GCST007090	1.99E-10	3.69649	Right pericalcarine volume (UKB data field 26909)
rs1793913	T	A	-25.3565	0.0641	0.144905	0.1437	12	48387025	0.0139	403124	4.05E-06	Knee osteoarthritis || id:ebi-a-GCST007090	3.00E-08	4.57583	Right pericalcarine volume (UKB data field 26909)
rs1846974	G	A	32.9252	-0.0254	0.883539	0.8852	5	87969927	0.0159	403124	0.1103	Knee osteoarthritis || id:ebi-a-GCST007090	2.93E-10	5.22442	Right pericalcarine volume (UKB data field 26909)
rs2170719	G	A	-24.3216	-0.0191	0.425976	0.4239	11	104012767	0.0094	403124	0.0420301	Knee osteoarthritis || id:ebi-a-GCST007090	6.90E-14	3.24729	Right pericalcarine volume (UKB data field 26909)
rs2490561	T	C	-26.112	-0.0148	0.687575	0.6902	1	2023504	0.0101	403124	0.1405	Knee osteoarthritis || id:ebi-a-GCST007090	7.68E-14	3.49295	Right pericalcarine volume (UKB data field 26909)
rs2942003	T	G	34.875	0.0107	0.363617	0.3635	17	44576704	0.0108	403124	0.3198	Knee osteoarthritis || id:ebi-a-GCST007090	2.54E-20	3.77565	Right pericalcarine volume (UKB data field 26909)
rs374654	G	A	-21.687	0.0151	0.757666	0.757	6	21009108	0.0109	403124	0.1654	Knee osteoarthritis || id:ebi-a-GCST007090	7.92E-09	3.75852	Right pericalcarine volume (UKB data field 26909)
rs4141825	A	G	-27.7823	-0.0175	0.519784	0.5228	13	80190848	0.0093	403124	0.0593895	Knee osteoarthritis || id:ebi-a-GCST007090	4.91E-18	3.20978	Right pericalcarine volume (UKB data field 26909)
rs4686671	C	T	-21.6995	-0.0018	0.734037	0.7364	3	193799549	0.0105	403124	0.8644	Knee osteoarthritis || id:ebi-a-GCST007090	2.48E-09	3.63904	Right pericalcarine volume (UKB data field 26909)
rs4953547	C	G	22.409	-0.0224	0.595362	0.5959	2	48322286	0.0095	403124	0.0181101	Knee osteoarthritis || id:ebi-a-GCST007090	2.37E-11	3.35398	Right pericalcarine volume (UKB data field 26909)
rs502610	A	T	-27.6502	-0.0065	0.348758	0.3494	8	108967800	0.0097	403124	0.506499	Knee osteoarthritis || id:ebi-a-GCST007090	2.33E-16	3.37036	Right pericalcarine volume (UKB data field 26909)
rs59585835	G	A	-23.5855	0.0101	0.736049	0.735	20	16979509	0.0105	403124	0.337	Knee osteoarthritis || id:ebi-a-GCST007090	9.30E-11	3.64093	Right pericalcarine volume (UKB data field 26909)
rs60900435	A	T	21.2898	-0.0058	0.65884	0.6578	2	60331136	0.0099	403124	0.554201	Knee osteoarthritis || id:ebi-a-GCST007090	5.09E-10	3.42481	Right pericalcarine volume (UKB data field 26909)
rs6097618	G	C	-23.4375	-0.0233	0.555347	0.5522	20	52448936	0.0094	403124	0.0127201	Knee osteoarthritis || id:ebi-a-GCST007090	4.68E-13	3.23978	Right pericalcarine volume (UKB data field 26909)
rs611384	G	A	25.4243	-0.027	0.845899	0.845	11	134025501	0.0128	403124	0.0352704	Knee osteoarthritis || id:ebi-a-GCST007090	1.20E-08	4.46029	Right pericalcarine volume (UKB data field 26909)
rs61142020	C	T	-26.3206	-0.0039	0.786243	0.7856	12	5310971	0.0113	403124	0.728799	Knee osteoarthritis || id:ebi-a-GCST007090	1.67E-11	3.9095	Right pericalcarine volume (UKB data field 26909)
rs61784835	C	T	-26.4903	0.0026	0.642529	0.6448	1	47974123	0.0097	403124	0.7863	Knee osteoarthritis || id:ebi-a-GCST007090	3.75E-15	3.36902	Right pericalcarine volume (UKB data field 26909)
rs61941460	A	C	-23.5045	-0.0151	0.81004	0.8102	12	130577055	0.0119	403124	0.2048	Knee osteoarthritis || id:ebi-a-GCST007090	1.27E-08	4.13038	Right pericalcarine volume (UKB data field 26909)
rs628380	C	G	-23.5352	0.0085	0.248071	0.2477	3	124880511	0.0108	403124	0.4331	Knee osteoarthritis || id:ebi-a-GCST007090	3.87E-10	3.76021	Right pericalcarine volume (UKB data field 26909)
rs6880658	C	T	-44.1835	0.0427	0.902778	0.9055	5	124238128	0.0159	403124	0.00737208	Knee osteoarthritis || id:ebi-a-GCST007090	6.10E-16	5.4633	Right pericalcarine volume (UKB data field 26909)
rs7214750	C	T	37.5636	0.0058	0.929816	0.9309	17	63903119	0.0185	403124	0.752801	Knee osteoarthritis || id:ebi-a-GCST007090	3.53E-09	6.36127	Right pericalcarine volume (UKB data field 26909)
rs72685500	A	T	24.2593	0.0117	0.700669	0.7002	4	157090189	0.0102	403124	0.2525	Knee osteoarthritis || id:ebi-a-GCST007090	5.97E-12	3.52591	Right pericalcarine volume (UKB data field 26909)
rs7296101	A	G	-23.4407	-0.0193	0.611316	0.6103	12	24234001	0.0096	403124	0.0436898	Knee osteoarthritis || id:ebi-a-GCST007090	1.54E-12	3.3153	Right pericalcarine volume (UKB data field 26909)
rs74580701	A	G	77.5227	0.0028	0.957597	0.9569	6	127000881	0.0228	403124	0.9014	Knee osteoarthritis || id:ebi-a-GCST007090	1.17E-22	7.91336	Right pericalcarine volume (UKB data field 26909)
rs74826997	T	C	65.1735	-0.0114	0.877327	0.875	14	59628609	0.0141	403124	0.4174	Knee osteoarthritis || id:ebi-a-GCST007090	2.89E-40	4.90636	Right pericalcarine volume (UKB data field 26909)
rs910697	A	G	24.8402	-0.0028	0.541999	0.5379	1	113063125	0.0093	403124	0.766699	Knee osteoarthritis || id:ebi-a-GCST007090	1.59E-14	3.23439	Right pericalcarine volume (UKB data field 26909)
rs9375411	A	G	-19.7555	-0.0012	0.48164	0.4811	6	126116953	0.0094	403124	0.9002	Knee osteoarthritis || id:ebi-a-GCST007090	1.15E-09	3.24566	Right pericalcarine volume (UKB data field 26909)
rs946711	A	C	20.8785	-0.0258	0.673059	0.6728	10	21806832	0.0099	403124	0.0092871	Knee osteoarthritis || id:ebi-a-GCST007090	1.21E-09	3.43431	Right pericalcarine volume (UKB data field 26909)
rs9632916	C	G	21.4833	-0.0053	0.764651	0.7687	9	98213728	0.0111	403124	0.633501	Knee osteoarthritis || id:ebi-a-GCST007090	1.88E-08	3.82103	Right pericalcarine volume (UKB data field 26909)
rs9830221	T	C	23.5471	-0.0046	0.551915	0.5531	3	104682417	0.0099	403124	0.6455	Knee osteoarthritis || id:ebi-a-GCST007090	5.06E-13	3.25975	Right pericalcarine volume (UKB data field 26909)
rs10057395	A	G	41.9708	-0.0098	0.471092	0.4703	5	60088794	0.0093	403124	0.2941	Knee osteoarthritis || id:ebi-a-GCST007090	4.19E-08	7.65518	Left lingual volume (UKB data field 26800)
rs10511145	C	A	-50.9295	-0.0078	0.603979	0.6012	3	89597679	0.0095	403124	0.4121	Knee osteoarthritis || id:ebi-a-GCST007090	7.05E-11	7.81175	Left lingual volume (UKB data field 26800)
rs10777730	G	C	-43.6707	0.0054	0.476076	0.4762	12	96161868	0.0093	403124	0.5613	Knee osteoarthritis || id:ebi-a-GCST007090	1.12E-08	7.64619	Left lingual volume (UKB data field 26800)
rs12490814	T	C	-50.3993	0.0069	0.518179	0.516	3	104654515	0.0093	403124	0.4561	Knee osteoarthritis || id:ebi-a-GCST007090	3.02E-11	7.58405	Left lingual volume (UKB data field 26800)
rs12550461	T	A	69.6409	-0.0141	0.877868	0.8758	8	78091799	0.0141	403124	0.3189	Knee osteoarthritis || id:ebi-a-GCST007090	2.24E-09	11.6473	Left lingual volume (UKB data field 26800)
rs13010134	T	G	49.4768	-0.0087	0.655949	0.6593	2	49368023	0.0098	403124	0.377	Knee osteoarthritis || id:ebi-a-GCST007090	8.59E-10	8.06646	Left lingual volume (UKB data field 26800)
rs2284871	C	T	47.2478	-0.0069	0.697185	0.6992	2	162888055	0.0101	403124	0.4942	Knee osteoarthritis || id:ebi-a-GCST007090	1.30E-08	8.30868	Left lingual volume (UKB data field 26800)
rs2863231	A	G	-49.0442	0.0062	0.357846	0.3568	11	31796560	0.0097	403124	0.5217	Knee osteoarthritis || id:ebi-a-GCST007090	8.08E-10	7.98323	Left lingual volume (UKB data field 26800)
rs2950706	C	G	87.7711	-8.00E-04	0.806617	0.8041	17	44343004	0.0129	403124	0.9523	Knee osteoarthritis || id:ebi-a-GCST007090	3.26E-18	10.0865	Left lingual volume (UKB data field 26800)
rs4718965	C	T	-65.224	0.0116	0.117792	0.1192	7	70040448	0.0144	403124	0.4204	Knee osteoarthritis || id:ebi-a-GCST007090	3.56E-08	11.8344	Left lingual volume (UKB data field 26800)
rs548953	C	T	-49.2512	-0.0097	0.344646	0.3456	8	108966869	0.0103	403124	0.3453	Knee osteoarthritis || id:ebi-a-GCST007090	7.85E-10	8.01102	Left lingual volume (UKB data field 26800)
rs61784835	C	T	-44.859	0.0026	0.642529	0.6448	1	47974123	0.0097	403124	0.7863	Knee osteoarthritis || id:ebi-a-GCST007090	1.81E-08	7.96855	Left lingual volume (UKB data field 26800)
rs61939338	C	T	-48.0846	0.0083	0.649373	0.6512	12	130570973	0.0098	403124	0.3957	Knee osteoarthritis || id:ebi-a-GCST007090	1.70E-09	7.98168	Left lingual volume (UKB data field 26800)
rs6450402	A	T	50.4154	-0.006	0.22964	0.2262	5	56040390	0.0111	403124	0.5915	Knee osteoarthritis || id:ebi-a-GCST007090	2.78E-08	9.07568	Left lingual volume (UKB data field 26800)
rs6695765	T	C	48.0721	8.00E-04	0.485427	0.4892	1	18979320	0.0093	403124	0.933	Knee osteoarthritis || id:ebi-a-GCST007090	2.89E-10	7.62502	Left lingual volume (UKB data field 26800)
rs6882046	A	G	54.0423	0.0034	0.733373	0.7348	5	87968864	0.0106	403124	0.7511	Knee osteoarthritis || id:ebi-a-GCST007090	6.25E-10	8.73908	Left lingual volume (UKB data field 26800)
rs729638	T	C	44.2685	-0.015	0.345567	0.3444	3	13827092	0.0098	403124	0.1268	Knee osteoarthritis || id:ebi-a-GCST007090	3.58E-08	8.0335	Left lingual volume (UKB data field 26800)
rs74580701	A	G	142.978	0.0028	0.957597	0.9569	6	127000881	0.0228	403124	0.9014	Knee osteoarthritis || id:ebi-a-GCST007090	2.29E-14	18.7316	Left lingual volume (UKB data field 26800)
rs74826997	T	C	114.466	-0.0114	0.877327	0.875	14	59628609	0.0141	403124	0.4174	Knee osteoarthritis || id:ebi-a-GCST007090	6.21E-23	11.6093	Left lingual volume (UKB data field 26800)
rs8043491	C	G	52.0338	-0.0157	0.34609	0.3465	15	98434914	0.0098	403124	0.1093	Knee osteoarthritis || id:ebi-a-GCST007090	7.11E-11	7.98272	Left lingual volume (UKB data field 26800)
rs910697	A	G	46.5096	-0.0028	0.541999	0.5379	1	113063125	0.0093	403124	0.766699	Knee osteoarthritis || id:ebi-a-GCST007090	1.18E-09	7.65013	Left lingual volume (UKB data field 26800)
rs9398171	C	T	-50.8171	-0.0244	0.2938	0.2888	6	108983527	0.0102	403124	0.01734	Knee osteoarthritis || id:ebi-a-GCST007090	1.01E-09	8.32043	Left lingual volume (UKB data field 26800)
rs9545155	T	C	-48.741	-0.0174	0.519769	0.5228	13	80191873	0.0093	403124	0.0604199	Knee osteoarthritis || id:ebi-a-GCST007090	1.44E-10	7.60247	Left lingual volume (UKB data field 26800)
rs10057395	A	G	27.9058	-0.0098	0.471092	0.4703	5	60088794	0.0093	403124	0.2941	Knee osteoarthritis || id:ebi-a-GCST007090	4.16E-20	3.03858	Left pericalcarine volume (UKB data field 26808)
rs1025876	C	A	18.5227	-0.0103	0.468944	0.4737	2	60012090	0.0094	403124	0.2732	Knee osteoarthritis || id:ebi-a-GCST007090	1.51E-09	3.06491	Left pericalcarine volume (UKB data field 26808)
rs10828247	A	G	21.7805	-0.0316	0.657232	0.6582	10	21822856	0.0098	403124	0.00124899	Knee osteoarthritis || id:ebi-a-GCST007090	6.78E-12	3.17394	Left pericalcarine volume (UKB data field 26808)
rs11020063	C	T	21.6683	3.00E-04	0.677785	0.6768	11	92542474	0.01	403124	0.975	Knee osteoarthritis || id:ebi-a-GCST007090	3.45E-11	3.27013	Left pericalcarine volume (UKB data field 26808)
rs11022131	C	G	28.3544	0.0029	0.750134	0.7513	11	12072213	0.0107	403124	0.7865	Knee osteoarthritis || id:ebi-a-GCST007090	4.28E-16	3.48747	Left pericalcarine volume (UKB data field 26808)
rs11047271	T	C	-19.8318	-0.0242	0.617411	0.6165	12	24267891	0.0096	403124	0.01148	Knee osteoarthritis || id:ebi-a-GCST007090	2.10E-10	3.12122	Left pericalcarine volume (UKB data field 26808)
rs11108237	A	G	-18.8085	-4.00E-04	0.667719	0.6662	12	96152816	0.0099	403124	0.9701	Knee osteoarthritis || id:ebi-a-GCST007090	5.35E-09	3.22295	Left pericalcarine volume (UKB data field 26808)
rs11918598	C	T	23.9469	0.0067	0.83139	0.8312	3	119024937	0.0124	403124	0.5905	Knee osteoarthritis || id:ebi-a-GCST007090	2.96E-09	4.03555	Left pericalcarine volume (UKB data field 26808)
rs12198009	G	T	-27.1412	0.0253	0.893874	0.8956	6	127175367	0.016	403124	0.114	Knee osteoarthritis || id:ebi-a-GCST007090	3.43E-08	4.91873	Left pericalcarine volume (UKB data field 26808)
rs12508031	A	T	-22.0276	-0.0184	0.656114	0.6546	4	19073685	0.0101	403124	0.0683597	Knee osteoarthritis || id:ebi-a-GCST007090	2.92E-11	3.31205	Left pericalcarine volume (UKB data field 26808)
rs12536836	C	T	-22.7793	0.0033	0.616811	0.6149	7	18881075	0.0096	403124	0.7339	Knee osteoarthritis || id:ebi-a-GCST007090	2.20E-13	3.105	Left pericalcarine volume (UKB data field 26808)
rs12550461	T	A	34.0127	-0.0141	0.877868	0.8758	8	78091799	0.0141	403124	0.3189	Knee osteoarthritis || id:ebi-a-GCST007090	1.94E-13	4.62583	Left pericalcarine volume (UKB data field 26808)
rs13082262	G	A	19.4652	-0.0033	0.38293	0.3834	3	141652082	0.0096	403124	0.7326	Knee osteoarthritis || id:ebi-a-GCST007090	4.69E-10	3.1249	Left pericalcarine volume (UKB data field 26808)
rs1635550	A	G	-26.5251	0.0477	0.165102	0.1637	12	48377970	0.0126	403124	0.0001532	Knee osteoarthritis || id:ebi-a-GCST007090	9.67E-11	4.09839	Left pericalcarine volume (UKB data field 26808)
rs17481712	A	G	-70.6702	-0.0112	0.985088	0.9851	1	88254265	0.0385	403124	0.7704	Knee osteoarthritis || id:ebi-a-GCST007090	1.83E-08	12.5592	Left pericalcarine volume (UKB data field 26808)
rs1846974	G	A	29.899	-0.0254	0.883539	0.8852	5	87969927	0.0159	403124	0.1103	Knee osteoarthritis || id:ebi-a-GCST007090	1.16E-09	4.91326	Left pericalcarine volume (UKB data field 26808)
rs1934057	C	T	20.3741	0.006	0.516785	0.5197	1	18962095	0.0094	403124	0.5251	Knee osteoarthritis || id:ebi-a-GCST007090	2.43E-11	3.05124	Left pericalcarine volume (UKB data field 26808)
rs2169404	G	A	-23.4587	0.0086	0.72407	0.7259	12	130580713	0.0111	403124	0.4376	Knee osteoarthritis || id:ebi-a-GCST007090	8.70E-12	3.4364	Left pericalcarine volume (UKB data field 26808)
rs2514845	T	G	-26.7194	-0.0061	0.351629	0.352	8	108993644	0.0097	403124	0.5303	Knee osteoarthritis || id:ebi-a-GCST007090	3.31E-17	3.16767	Left pericalcarine volume (UKB data field 26808)
rs372645904	A	G	28.6449	0.0046	0.325569	0.3269	17	44652561	0.0118	403124	0.6951	Knee osteoarthritis || id:ebi-a-GCST007090	4.48E-15	3.65339	Left pericalcarine volume (UKB data field 26808)
rs374567888	G	T	-20.5279	-0.0198	0.424223	0.4224	11	104010484	0.0099	403124	0.0461796	Knee osteoarthritis || id:ebi-a-GCST007090	1.92E-11	3.05846	Left pericalcarine volume (UKB data field 26808)
rs4141825	A	G	-20.5318	-0.0175	0.519784	0.5228	13	80190848	0.0093	403124	0.0593895	Knee osteoarthritis || id:ebi-a-GCST007090	1.00E-11	3.01665	Left pericalcarine volume (UKB data field 26808)
rs4953547	C	G	19.3792	-0.0224	0.595362	0.5959	2	48322286	0.0095	403124	0.0181101	Knee osteoarthritis || id:ebi-a-GCST007090	7.64E-10	3.14997	Left pericalcarine volume (UKB data field 26808)
rs59585835	G	A	-19.6225	0.0101	0.736049	0.735	20	16979509	0.0105	403124	0.337	Knee osteoarthritis || id:ebi-a-GCST007090	9.77E-09	3.42177	Left pericalcarine volume (UKB data field 26808)
rs6097618	G	C	-26.6457	-0.0233	0.555347	0.5522	20	52448936	0.0094	403124	0.0127201	Knee osteoarthritis || id:ebi-a-GCST007090	2.11E-18	3.04477	Left pericalcarine volume (UKB data field 26808)
rs61784835	C	T	-25.9907	0.0026	0.642529	0.6448	1	47974123	0.0097	403124	0.7863	Knee osteoarthritis || id:ebi-a-GCST007090	2.00E-16	3.16098	Left pericalcarine volume (UKB data field 26808)
rs6537298	C	T	18.4975	-0.0128	0.445659	0.4454	4	145506871	0.0093	403124	0.1688	Knee osteoarthritis || id:ebi-a-GCST007090	1.06E-09	3.03235	Left pericalcarine volume (UKB data field 26808)
[illegible]	[illegible]	[illegible]	[illegible]	[illegible]	[illegible]	[illegible]	[illegible]	[illegible]	[illegible]	[illegible]	[illegible]	[illegible]	[illegible]	[illegible]	[illegible]

Table S3

exposure	method	nsnp	b	se	pval	lo_ci	up_ci	or	or_lci95	or_uci95
Left pars opercularis volume (UKB data field 26805)	MR Egger	9	-0.000278349	0.00038471	0.492817661	-0.00103238	0.000475682	0.99972169	0.998968152	1.000475795
Left pars opercularis volume (UKB data field 26805)	Weighted median	9	-0.0001872	0.000123191	0.128613462	-0.000428654	5.42542E-05	0.999812818	0.999571438	1.000054256
Left pars opercularis volume (UKB data field 26805)	Inverse variance weighted	9	-0.00016916	0.000136515	0.215297072	-0.000436728	9.84092E-05	0.999830855	0.999563367	1.000098414
Left pars opercularis volume (UKB data field 26805)	Simple mode	9	-0.000171725	0.000161498	0.318673606	-0.00048826	0.00014481	0.99982829	0.999511859	1.000144821
Left pars opercularis volume (UKB data field 26805)	Weighted mode	9	-0.000183414	0.000151265	0.259897096	-0.000479895	0.000113066	0.999816602	0.999520221	1.000113072
Right pallidum volume (UKB data field 26592)	MR Egger	30	8.24405E-05	0.000940155	0.930748661	-0.001760263	0.001925144	1.000082444	0.998241285	1.001926999
Right pallidum volume (UKB data field 26592)	Weighted median	30	-0.000498289	0.000277326	0.072372935	-0.001041848	4.52693E-05	0.999501835	0.998958695	1.00004527
Right pallidum volume (UKB data field 26592)	Inverse variance weighted	30	-0.000350718	0.00026148	0.179828937	-0.000863218	0.000161782	0.999649344	0.999137155	1.000161795
Right pallidum volume (UKB data field 26592)	Simple mode	30	-0.000674586	0.000578279	0.252899838	-0.001808013	0.000458841	0.999325641	0.99819362	1.000458947
Right pallidum volume (UKB data field 26592)	Weighted mode	30	-0.000490515	0.000627392	0.440649782	-0.001720203	0.000739172	0.999509605	0.998281276	1.000739446
Left hippocampus volume (UKB data field 26562)	MR Egger	20	0.001111405	0.00035142	0.00538721	0.000422622	0.001800189	1.001112023	1.000422711	1.00180181
Left hippocampus volume (UKB data field 26562)	Weighted median	20	9.06093E-05	0.000164762	0.58236067	-0.000232325	0.000413543	1.000090613	0.999767702	1.000413629
Left hippocampus volume (UKB data field 26562)	Inverse variance weighted	20	-1.9067E-05	0.000170617	0.911019317	-0.000353477	0.000315343	0.999980933	0.999646586	1.000315393
Left hippocampus volume (UKB data field 26562)	Simple mode	20	-0.000109448	0.00028044	0.700675429	-0.000659111	0.000440214	0.999890558	0.999341106	1.000440311
Left hippocampus volume (UKB data field 26562)	Weighted mode	20	6.15654E-05	0.000269715	0.82188242	-0.000467077	0.000590208	1.000061567	0.999533032	1.000590382
Right pars opercularis volume (UKB data field 26906)	MR Egger	7	6.62564E-05	0.000586101	0.914392596	-0.001082502	0.001215015	1.000066259	0.998918084	1.001215753
Right pars opercularis volume (UKB data field 26906)	Weighted median	7	-0.000184308	0.000159231	0.247074085	-0.000496401	0.000127785	0.999815709	0.999503722	1.000127794
Right pars opercularis volume (UKB data field 26906)	Inverse variance weighted	7	-0.000334156	0.000241449	0.166369993	-0.000807395	0.000139083	0.9996659	0.999192931	1.000139093
Right pars opercularis volume (UKB data field 26906)	Simple mode	7	-6.98628E-05	0.00020306	0.742546125	-0.000467861	0.000328135	0.99993014	0.999532248	1.000328189
Right pars opercularis volume (UKB data field 26906)	Weighted mode	7	-0.000134612	0.00016553	0.447155351	-0.000459052	0.000189828	0.999865397	0.999541054	1.000189846
Right isthmus cingulate volume (UKB data field 26898)	MR Egger	8	-0.000193403	0.001510815	0.902322026	-0.003154601	0.002767795	0.999806616	0.99685037	1.002771629
Right isthmus cingulate volume (UKB data field 26898)	Weighted median	8	0.000453105	0.000288648	0.1164731	-0.000112644	0.001018854	1.000453207	0.999887362	1.001019373
Right isthmus cingulate volume (UKB data field 26898)	Inverse variance weighted	8	0.000271131	0.000273415	0.32137022	-0.000264762	0.000807024	1.000271167	0.999735273	1.00080735
Right isthmus cingulate volume (UKB data field 26898)	Simple mode	8	0.000780054	0.000535786	0.188756023	-0.000270086	0.001830195	1.000780359	0.999729951	1.001831871
Right isthmus cingulate volume (UKB data field 26898)	Weighted mode	8	0.000651166	0.000418149	0.163366159	-0.000168405	0.001470737	1.000651378	0.999831609	1.001471819
Left inferior parietal volume (UKB data field 26795)	MR Egger	9	-1.95015E-05	0.000340041	0.955868828	-0.000685982	0.000646979	0.999980499	0.999314253	1.000647188
Left inferior parietal volume (UKB data field 26795)	Weighted median	9	2.05107E-05	6.0338E-05	0.733909764	-9.77519E-05	0.000138773	1.000020511	0.999902253	1.000138783
Left inferior parietal volume (UKB data field 26795)	Inverse variance weighted	9	4.88891E-06	5.42603E-05	0.928206907	-0.000101461	0.000111239	1.000004889	0.999898544	1.000111245
Left inferior parietal volume (UKB data field 26795)	Simple mode	9	4.06317E-05	8.22536E-05	0.634596048	-0.000120585	0.000201849	1.000040633	0.999879422	1.000201869
Left inferior parietal volume (UKB data field 26795)	Weighted mode	9	4.24804E-05	8.2047E-05	0.618637053	-0.000118332	0.000203293	1.000042481	0.999881675	1.000203313
Right superior temporal volume (UKB data field 26918)	MR Egger	12	-0.00034697	0.00024124	0.180905884	-0.000819799	0.00012586	0.999653091	0.999180537	1.000125868
Right superior temporal volume (UKB data field 26918)	Weighted median	12	-5.14302E-05	6.42028E-05	0.423097863	-0.000177268	7.44073E-05	0.999948571	0.999822748	1.00007441
Right superior temporal volume (UKB data field 26918)	Inverse variance weighted	12	-6.4603E-05	6.20634E-05	0.297912627	-0.000186247	5.70412E-05	0.999935399	0.99981377	1.000057043
Right superior temporal volume (UKB data field 26918)	Simple mode	12	-8.15073E-05	0.000106848	0.461608137	-0.000290929	0.000127915	0.999918496	0.999709113	1.000127923
Right superior temporal volume (UKB data field 26918)	Weighted mode	12	-7.29077E-05	9.79291E-05	0.472183014	-0.000264849	0.000119033	0.999927095	0.999735186	1.00011904
Right pericalcarine volume (UKB data field 26909)	MR Egger	42	-0.00016347	0.000326189	0.619011072	-0.000802801	0.00047586	0.999836543	0.999197521	1.000475974
Right pericalcarine volume (UKB data field 26909)	Weighted median	42	-8.51221E-05	0.000101504	0.401688341	-0.00028407	0.000113825	0.999914881	0.999715971	1.000113832
Right pericalcarine volume (UKB data field 26909)	Inverse variance weighted	42	-6.04557E-05	9.48334E-05	0.52380335	-0.000246329	0.000125418	0.999939546	0.999753701	1.000125426
Right pericalcarine volume (UKB data field 26909)	Simple mode	42	-6.11781E-05	0.000204721	0.766573884	-0.000462431	0.000340074	0.999938824	0.999537676	1.000340132
Right pericalcarine volume (UKB data field 26909)	Weighted mode	42	-8.35272E-05	0.000159799	0.603994705	-0.000396733	0.000229678	0.999916476	0.999603346	1.000229705
Right rostral middle frontal volume (UKB data field 26915)	MR Egger	12	-0.000276366	0.000189288	0.17497224	-0.00064737	9.46378E-05	0.999723672	0.999352839	1.000094642
Right rostral middle frontal volume (UKB data field 26915)	Weighted median	12	-3.19579E-05	4.86965E-05	0.511652831	-0.000127403	6.34873E-05	0.999968043	0.999872605	1.000063489
Right rostral middle frontal volume (UKB data field 26915)	Inverse variance weighted	12	-6.00019E-05	5.09497E-05	0.23892872	-0.000159863	3.98596E-05	0.99994	0.999840149	1.00003986
Right rostral middle frontal volume (UKB data field 26915)	Simple mode	12	9.40252E-07	9.80246E-05	0.992518566	-0.000191188	0.000193069	1.00000094	0.99980883	1.000193087
Right rostral middle frontal volume (UKB data field 26915)	Weighted mode	12	-9.29908E-06	7.91652E-05	0.908609475	-0.000164463	0.000145865	0.999990701	0.999835551	1.000145875
Left parahippocampal volume (UKB data field 26803)	MR Egger	7	0.003333047	0.002681889	0.269044505	-0.001923456	0.008589549	1.003338607	0.998078393	1.008626545
Left parahippocampal volume (UKB data field 26803)	Weighted median	7	0.000741662	0.000359461	0.039088076	3.71174E-05	0.001446206	1.000741937	1.000037118	1.001447252
Left parahippocampal volume (UKB data field 26803)	Inverse variance weighted	7	0.000757923	0.000269344	0.004893548	0.000230009	0.001285838	1.000758211	1.000230035	1.001286665
Left parahippocampal volume (UKB data field 26803)	Simple mode	7	0.000787306	0.00062395	0.253849979	-0.000435636	0.002010247	1.000787616	0.999564459	1.002012269
Left parahippocampal volume (UKB data field 26803)	Weighted mode	7	0.000747384	0.000564372	0.233620569	-0.000358786	0.001853553	1.000747663	0.999641279	1.001855272
Right supramarginal volume (UKB data field 26919)	MR Egger	16	-2.34489E-06	0.000125159	0.98531663	-0.000247656	0.000242966	0.999997655	0.999752374	1.000242996
Right supramarginal volume (UKB data field 26919)	Weighted median	16	1.70208E-05	4.73534E-05	0.719264808	-7.57919E-05	0.000109833	1.000017021	0.999924211	1.000109839
Right supramarginal volume (UKB data field 26919)	Inverse variance weighted	16	7.07326E-06	4.04084E-05	0.861044998	-7.21273E-05	8.62738E-05	1.000007073	0.999927875	1.000086278
Right supramarginal volume (UKB data field 26919)	Simple mode	16	1.21491E-05	8.36092E-05	0.886402009	-0.000151725	0.000176023	1.000012149	0.999848287	1.000176039
Right supramarginal volume (UKB data field 26919)	Weighted mode	16	2.7472E-05	7.20642E-05	0.708388399	-0.000113774	0.000168718	1.000027472	0.999886233	1.000168732
Left middle temporal volume (UKB data field 26802)	MR Egger	14	-0.000216342	0.000303435	0.489493549	-0.000811076	0.000378391	0.999783681	0.999189253	1.000378463
Left middle temporal volume (UKB data field 26802)	Weighted median	14	-4.89351E-05	6.91197E-05	0.478960374	-0.00018441	8.65395E-05	0.999951066	0.999815607	1.000086543
Left middle temporal volume (UKB data field 26802)	Inverse variance weighted	14	-2.20723E-05	7.89904E-05	0.779914525	-0.000176893	0.000132749	0.999977928	0.999823122	1.000132758
Left middle temporal volume (UKB data field 26802)	Simple mode	14	-9.77779E-05	0.00017914	0.594433317	-0.000448892	0.000253336	0.999902227	0.999551209	1.000253368
Left middle temporal volume (UKB data field 26802)	Weighted mode	14	-0.000101419	0.000162701	0.543838	-0.000420313	0.000217475	0.999898586	0.999579775	1.000217498
Left fusiform volume (UKB data field 26794)	MR Egger	8	3.71639E-05	0.000191205	0.852302727	-0.000337598	0.000411926	1.000037165	0.999662459	1.000412011
Left fusiform volume (UKB data field 26794)	Weighted median	8	-1.37093E-05	7.20559E-05	0.84910629	-0.000154939	0.00012752	0.999986291	0.999845073	1.000127528
Left fusiform volume (UKB data field 26794)	Inverse variance weighted	8	9.15985E-06	6.11192E-05	0.88086825	-0.000110634	0.000128954	1.00000916	0.999889372	1.000128962
Left fusiform volume (UKB data field 26794)	Simple mode	8	-4.63979E-05	0.000116825	0.703082277	-0.000275375	0.000182579	0.999953603	0.999724663	1.000182596
Left fusiform volume (UKB data field 26794)	Weighted mode	8	4.72047E-06	8.04562E-05	0.954853651	-0.000152974	0.000162415	1.00000472	0.999847038	1.000162428
Left pericalcarine volume (UKB data field 26808)	MR Egger	37	-0.000325823	0.000301903	0.287866094	-0.000917554	0.000265907	0.99967423	0.999082867	1.000265942
Left pericalcarine volume (UKB data field 26808)	Weighted median	37	-0.000147258	0.000113713	0.195322275	-0.000370136	7.56199E-05	0.999852753	0.999629933	1.000075623
Left pericalcarine volume (UKB data field 26808)	Inverse variance weighted	37	-0.000143504	9.74728E-05	0.140953411	-0.000334551	4.75424E-05	0.999856506	0.999665505	1.000047543
Left pericalcarine volume (UKB data field 26808)	Simple mode	37	-5.38131E-05	0.000192326	0.781231109	-0.000430772	0.000323146	0.999946188	0.999569321	1.000323198
Left pericalcarine volume (UKB data field 26808)	Weighted mode	37	-0.000115943	0.000145923	0.432084604	-0.000401951	0.000170066	0.999884064	0.999598129	1.000170081
Right precentral volume (UKB data field 26912)	MR Egger	11	2.37895E-05	8.01964E-05	0.773472945	-0.000133395	0.000180974	1.00002379	0.999866613	1.000180991
Right precentral volume (UKB data field 26912)	Weighted median	11	4.8451E-05	3.76672E-05	0.198340557	-2.53766E-05	0.000122279	1.000048452	0.999974624	1.000122286
Right precentral volume (UKB data field 26912)	Inverse variance weighted	11	3.50297E-05	4.48943E-05	0.435232151	-5.29632E-05	0.000123022	1.00003503	0.999947038	1.00012303
Right precentral volume (UKB data field 26912)	Simple mode	11	9.39048E-05	9.39321E-05	0.34102697	-9.02021E-05	0.000278012	1.000093909	0.999909802	1.00027805
Right precentral volume (UKB data field 26912)	Weighted mode	11	5.12217E-05	4.1334E-05	0.243561383	-2.97929E-05	0.000132236	1.000051223	0.999970208	1.000132245
Right caudate volume (UKB data field 26590)	MR Egger	25	0.00058541	0.000666342	0.388735688	-0.000720621	0.00189144	1.000585581	0.999279639	1.00189323
Right caudate volume (UKB data field 26590)	Weighted median	25	2.22364E-05	0.000155404	0.886220593	-0.000282355	0.000326827	1.000022237	0.999717685	1.000326881
Right caudate volume (UKB data field 26590)	Inverse variance weighted	25	-2.31367E-05	0.000143963	0.872319683	-0.000305304	0.000259031	0.999976864	0.999694742	1.000259064
Right caudate volume (UKB data field 26590)	Simple mode	25	5.2434E-05	0.00030146	0.863376536	-0.000538428	0.000643296	1.000052435	0.999461717	1.000643503
Right caudate volume (UKB data field 26590)	Weighted mode	25	2.99071E-05	0.00025584	0.907913955	-0.000471539	0.000531353	1.000029908	0.999528572	1.000531494
Right entorhinal volume (UKB data field 26894)	MR Egger	4	0.00060899	0.003527898	0.878837623	-0.00630569	0.00752367	1.000609176	0.993714149	1.007552044
Right entorhinal volume (UKB data field 26894)	Weighted median	4	4.6035E-05	0.000332494	0.889881801	-0.000605653	0.000697723	1.000046036	0.999394531	1.000697966
Right entorhinal volume (UKB data field 26894)	Inverse variance weighted	4	0.000252426	0.000387028	0.514260652	-0.000506148	0.001011	1.000252458	0.99949398	1.001011512
Right entorhinal volume (UKB data field 26894)	Simple mode	4	-0.000152823	0.000492609	0.776687227	-0.001118337	0.00081269	0.999847188	0.998882288	1.000813021
Right entorhinal volume (UKB data field 26894)	Weighted mode	4	-0.000136129	0.000442026	0.778251383	-0.0010025	0.000730241	0.99986388	0.998998002	1.000730508
Left lateral occipital volume (UKB data field 26798)	MR Egger	9	-0.000131449	0.000141979	0.385338463	-0.000409727	0.000146829	0.99986856	0.999590357	1.00014684
Left lateral occipital volume (UKB data field 26798)	Weighted median	9	-2.26327E-05	5.52439E-05	0.682035518	-0.000130911	8.56453E-05	0.999977368	0.999869098	1.000085649
Left lateral occipital volume (UKB data field 26798)	Inverse variance weighted	9	9.49282E-06	4.3088E-05	0.825627859	-7.49597E-05	9.39453E-05	1.000009493	0.999925043	1.00009395
Left lateral occipital volume (UKB data field 26798)	Simple mode	9	-3.00981E-05	9.71128E-05	0.764534303	-0.000220439	0.000160243	0.999969902	0.999779585	1.000160256
Left lateral occipital volume (UKB data field 26798)	Weighted mode	9	-4.49177E-05	9.32806E-05	0.643033503	-0.000227748	0.000137912	0.999955083	0.999772278	1.000137922
Left caudal middle frontal volume (UKB data field 26791)	MR Egger	14	-0.000164861	0.00025685	0.533035146	-0.000668288	0.000338565	0.999835152	0.999331935	1.000338623
Left caudal middle frontal volume (UKB data field 26791)	Weighted median	14	-5.54155E-05	7.558E-05	0.463434905	-0.000203552	9.27214E-05	0.999944586	0.999796468	1.000092726
Left caudal middle frontal volume (UKB data field 26791)	Inverse variance weighted	14	-7.18243E-05	8.71324E-05	0.409761958	-0.000242604	9.89552E-05	0.999928178	0.999757426	1.00009896
Left caudal middle frontal volume (UKB data field 26791)	Simple mode	14	-1.3381E-05	0.000130429	0.919852079	-0.000269021	0.000242259	0.999986619	0.999731015	1.000242289
Left caudal middle frontal volume (UKB data field 26791)	Weighted mode	14	-0.000114996	8.35319E-05	0.191862938	-0.000278718	4.8727E-05	0.999885011	0.999721321	1.000048728
Left ventral diencephalon (DC) volume (UKB data field 26565)	MR Egger	35	0.000253885	0.000437623	0.56575655	-0.000603856	0.001111626	1.000253918	0.999396327	1.001112245
Left ventral diencephalon (DC) volume (UKB data field 26565)	Weighted median	35	-8.08157E-05	0.000151981	0.594900971	-0.000378699	0.000217068	0.999919188	0.999621373	1.000217091
Left ventral diencephalon (DC) volume (UKB data field 26565)	Inverse variance weighted	35	-0.00018139	0.000141977	0.201387837	-0.000459664	9.68837E-05	0.999818626	0.999540441	1.000096888
Left ventral diencephalon (DC) volume (UKB data field 26565)	Simple mode	35	6.87231E-05	0.000297096	0.818455843	-0.000513585	0.000651031	1.000068725	0.999486547	1.000651243
Left ventral diencephalon (DC) volume (UKB data field 26565)	Weighted mode	35	1.28101E-05	0.000237182	0.957243623	-0.000452066	0.000477686	1.00001281	0.999548036	1.0004778
Left pallidum volume (UKB data field 26561)	MR Egger	30	0.000361117	0.001148096	0.755446215	-0.001889151	0.002611384	1.000361182	0.998112633	1.002614797
Left pallidum volume (UKB data field 26561)	Weighted median	30	5.69803E-06	0.000282112	0.983885633	-0.000547242	0.000558638	1.000005698	0.999452907	1.000558794
Left pallidum volume (UKB data field 26561)	Inverse variance weighted	30	-0.000258655	0.000278113	0.35235289	-0.000803756	0.000286447	0.999741379	0.999196567	1.000286488
Left pallidum volume (UKB data field 26561)	Simple mode	30	-0.000275421	0.000607114	0.653453313	-0.001465365	0.000914523	0.999724617	0.998535708	1.000914942
Left pallidum volume (UKB data field 26561)	Weighted mode	30	0.000150205	0.000672339	0.824785754	-0.00116758	0.001467989	1.000150216	0.998833101	1.001469068
Right medial orbitofrontal volume (UKB data field 26902)	MR Egger	9	-0.002045841	0.001081874	0.100524983	-0.004166313	7.4631E-05	0.99795625	0.995842354	1.000074634
Right medial orbitofrontal volume (UKB data field 26902)	Weighted median	9	-0.000253763	0.000172897	0.142182248	-0.000592642	8.51151E-05	0.999746269	0.999407534	1.000085119
Right medial orbitofrontal volume (UKB data field 26902)	Inverse variance weighted	9	-0.000186561	0.000216843	0.389595615	-0.000611573	0.000238451	0.999813456	0.999388614	1.000238479
Right medial orbitofrontal volume (UKB data field 26902)	Simple mode	9	-0.000359562	0.000363849	0.351988682	-0.001072707	0.000353582	0.999640502	0.998927868	1.000353645
Right medial orbitofrontal volume (UKB data field 26902)	Weighted mode	9	-0.00050262	0.000294622	0.12640257	-0.001080079	7.48388E-05	0.999497506	0.998920504	1.000074842
Left lingual volume (UKB data field 26800)	MR Egger	22	-4.019E-05	0.000147912	0.78862592	-0.000330098	0.000249718	0.999959811	0.999669957	1.000249749
Left lingual volume (UKB data field 26800)	Weighted median	22	-8.73167E-05	5.47106E-05	0.110494307	-0.000194549	1.9916E-05	0.999912687	0.99980547	1.000019916
Left lingual volume (UKB data field 26800)	Inverse variance weighted	22	-4.31528E-05	4.0763E-05	0.289770236	-0.000123048	3.67427E-05	0.999956848	0.999876959	1.000036743
Left lingual volume (UKB data field 26800)	Simple mode	22	-0.000138587	9.52317E-05	0.160382914	-0.000325241	4.80669E-05	0.999861422	0.999674812	1.000048068
Left lingual volume (UKB data field 26800)	Weighted mode	22	-0.000116816	8.89953E-05	0.203479088	-0.000291247	5.76145E-05	0.999883191	0.999708795	1.000057616
Left rostral middle frontal volume (UKB data field 26814)	MR Egger	20	-0.000210756	0.00011513	0.083768667	-0.000436411	1.4898E-05	0.999789266	0.999563684	1.000014898
Left rostral middle frontal volume (UKB data field 26814)	Weighted median	20	-2.13377E-05	3.82396E-05	0.576843848	-9.62874E-05	5.36119E-05	0.999978662	0.999903717	1.000053613
Left rostral middle frontal volume (UKB data field 26814)	Inverse variance weighted	20	-1.00325E-05	3.55179E-05	0.777588999	-7.96476E-05	5.95827E-05	0.999989968	0.999920356	1.000059584
Left rostral middle frontal volume (UKB data field 26814)	Simple mode	20	-9.35387E-09	8.27847E-05	0.999911025	-0.000162267	0.000162249	0.999999991	0.999837746	1.000162262
Left rostral middle frontal volume (UKB data field 26814)	Weighted mode	20	-1.86668E-06	0.000100842	0.985424349	-0.000199518	0.000195784	0.999998133	0.999800502	1.000195804
Right posterior cingulate volume (UKB data field 26911)	MR Egger	5	0.000282443	0.001212078	0.830736869	-0.00209323	0.002658117	1.000282483	0.997908959	1.002661653
Right posterior cingulate volume (UKB data field 26911)	Weighted median	5	-0.000311695	0.000271716	0.251326434	-0.000844259	0.000220869	0.999688354	0.999156097	1.000220894
Right posterior cingulate volume (UKB data field 26911)	Inverse variance weighted	5	-0.000503855	0.000198684	0.011214008	-0.000893276	-0.000114434	0.999496272	0.999107123	0.999885573
Right posterior cingulate volume (UKB data field 26911)	Simple mode	5	-0.000198993	0.000399505	0.644556457	-0.000982022	0.000584037	0.999801027	0.99901846	1.000584208
Right posterior cingulate volume (UKB data field 26911)	Weighted mode	5	-0.000185151	0.000386933	0.657269074	-0.00094354	0.000573239	0.999814867	0.999056905	1.000573403
Right amygdala volume (UKB data field 26594)	MR Egger	14	0.002075171	0.00129868	0.136046423	-0.000470242	0.004620584	1.002077326	0.999529868	1.004631276
Right amygdala volume (UKB data field 26594)	Weighted median	14	0.000381746	0.000366796	0.297987255	-0.000337174	0.001100666	1.000381819	0.999662883	1.001101272
Right amygdala volume (UKB data field 26594)	Inverse variance weighted	14	0.000421575	0.000341213	0.216637469	-0.000247202	0.001090352	1.000421664	0.999752828	1.001090947
Right amygdala volume (UKB data field 26594)	Simple mode	14	0.00029568	0.000609807	0.635833019	-0.000899541	0.001490902	1.000295724	0.999100863	1.001492014
Right amygdala volume (UKB data field 26594)	Weighted mode	14	0.000226871	0.000510948	0.664329666	-0.000774587	0.001228328	1.000226896	0.999225713	1.001229083
Left thalamus proper volume (UKB data field 26558)	MR Egger	24	-0.000121375	0.000273617	0.66166721	-0.000657665	0.000414914	0.999878632	0.999342552	1.000415
Left thalamus proper volume (UKB data field 26558)	Weighted median	24	-2.46787E-05	9.70221E-05	0.79921613	-0.000214842	0.000165485	0.999975322	0.999785181	1.000165498
Left thalamus proper volume (UKB data field 26558)	Inverse variance weighted	24	-5.40158E-05	7.94624E-05	0.49665338	-0.000209762	0.000101731	0.999945986	0.99979026	1.000101736
Left thalamus proper volume (UKB data field 26558)	Simple mode	24	-0.000135447	0.000196226	0.496938437	-0.00052005	0.000249156	0.999864562	0.999480085	1.000249187
Left thalamus proper volume (UKB data field 26558)	Weighted mode	24	-4.23115E-05	0.000160866	0.794874648	-0.000357609	0.000272986	0.999957689	0.999642455	1.000273023
Right superior frontal volume (UKB data field 26916)	MR Egger	26	-0.000115291	7.82114E-05	0.153451675	-0.000268586	3.80029E-05	0.999884715	0.99973145	1.000038004
Right superior frontal volume (UKB data field 26916)	Weighted median	26	-2.97077E-05	2.65254E-05	0.262725665	-8.16974E-05	2.2282E-05	0.999970293	0.999918306	1.000022282
Right superior frontal volume (UKB data field 26916)	Inverse variance weighted	26	-3.24377E-05	2.44574E-05	0.184741925	-8.03742E-05	1.54988E-05	0.999967563	0.999919629	1.000015499
Right superior frontal volume (UKB data field 26916)	Simple mode	26	-2.43483E-05	6.11126E-05	0.693705388	-0.000144129	9.54324E-05	0.999975652	0.999855881	1.000095437
Right superior frontal volume (UKB data field 26916)	Weighted mode	26	-4.80811E-05	4.47478E-05	0.292865337	-0.000135787	3.96247E-05	0.99995192	0.999864222	1.000039626
Right ventral diencephalon (DC) volume (UKB data field 26596)	MR Egger	34	0.000179673	0.000420669	0.672157949	-0.000644839	0.001004185	1.000179689	0.999355369	1.00100469
Right ventral diencephalon (DC) volume (UKB data field 26596)	Weighted median	34	-0.000176707	0.000150438	0.240148934	-0.000471566	0.000118152	0.999823308	0.999528545	1.000118159
Right ventral diencephalon (DC) volume (UKB data field 26596)	Inverse variance weighted	34	-0.00013681	0.000137949	0.321322072	-0.000407189	0.000133569	0.9998632	0.999592894	1.000133578
Right ventral diencephalon (DC) volume (UKB data field 26596)	Simple mode	34	-0.000156127	0.000336869	0.646072909	-0.00081639	0.000504136	0.999843885	0.999183943	1.000504263
Right ventral diencephalon (DC) volume (UKB data field 26596)	Weighted mode	34	-7.14539E-05	0.000254062	0.780277432	-0.000569415	0.000426507	0.999928549	0.999430747	1.000426598
Left amygdala volume (UKB data field 26563)	MR Egger	16	0.000810421	0.001354322	0.559132949	-0.001844049	0.003464892	1.00081075	0.99815765	1.003470901
Left amygdala volume (UKB data field 26563)	Weighted median	16	0.00010014	0.00038184	0.793122902	-0.000648266	0.000848547	1.000100145	0.999351944	1.000848907
Left amygdala volume (UKB data field 26563)	Inverse variance weighted	16	0.000163041	0.000389206	0.675285072	-0.000599804	0.000925885	1.000163054	0.999400376	1.000926314
Left amygdala volume (UKB data field 26563)	Simple mode	16	0.000240777	0.000617803	0.702213345	-0.000970116	0.00145167	1.000240806	0.999030354	1.001452724
Left amygdala volume (UKB data field 26563)	Weighted mode	16	-3.84112E-05	0.000530653	0.94325203	-0.00107849	0.001001668	0.99996159	0.998922091	1.00100217
Right superior parietal volume (UKB data field 26917)	MR Egger	14	-6.35885E-05	0.000151987	0.683059317	-0.000361484	0.000234307	0.999936414	0.999638582	1.000234334
Right superior parietal volume (UKB data field 26917)	Weighted median	14	-4.24589E-05	4.59373E-05	0.355340514	-0.000132496	4.75781E-05	0.999957542	0.999867513	1.000047579
Right superior parietal volume (UKB data field 26917)	Inverse variance weighted	14	5.93224E-06	3.84637E-05	0.87742893	-6.94567E-05	8.13212E-05	1.000005932	0.999930546	1.000081324
Right superior parietal volume (UKB data field 26917)	Simple mode	14	-8.48685E-05	8.5807E-05	0.340685277	-0.00025305	8.33133E-05	0.999915135	0.999746982	1.000083317
Right superior parietal volume (UKB data field 26917)	Weighted mode	14	-8.48685E-05	8.57851E-05	0.340566217	-0.000253007	8.32703E-05	0.999915135	0.999747025	1.000083274
Right pars triangularis volume (UKB data field 26908)	MR Egger	7	-7.4274E-05	0.000301143	0.814992955	-0.000664515	0.000515967	0.999925729	0.999335706	1.0005161
Right pars triangularis volume (UKB data field 26908)	Weighted median	7	-0.000145138	0.000125188	0.246308263	-0.000390506	0.00010023	0.999854872	0.99960957	1.000100235
Right pars triangularis volume (UKB data field 26908)	Inverse variance weighted	7	-0.000167596	0.000110675	0.129949638	-0.000384519	4.93279E-05	0.999832418	0.999615555	1.000049329
Right pars triangularis volume (UKB data field 26908)	Simple mode	7	-5.82886E-05	0.000182696	0.760506445	-0.000416373	0.000299796	0.999941713	0.999583713	1.000299841
Right pars triangularis volume (UKB data field 26908)	Weighted mode	7	-0.000164691	0.00016949	0.368731662	-0.000496891	0.000167508	0.999835322	0.999503233	1.000167522
Right putamen volume (UKB data field 26591)	MR Egger	29	0.000269492	0.000440568	0.545858492	-0.000594022	0.001133005	1.000269528	0.999406155	1.001133647
Right putamen volume (UKB data field 26591)	Weighted median	29	5.38115E-05	0.000126159	0.669715991	-0.00019346	0.000301083	1.000053813	0.999806559	1.000301128
Right putamen volume (UKB data field 26591)	Inverse variance weighted	29	0.000113289	0.000120907	0.348761222	-0.000123688	0.000350266	1.000113295	0.99987632	1.000350327
Right putamen volume (UKB data field 26591)	Simple mode	29	-3.32561E-05	0.000302239	0.913168469	-0.000625645	0.000559133	0.999966744	0.999374551	1.000559289
Right putamen volume (UKB data field 26591)	Weighted mode	29	-0.000118097	0.000265256	0.659584966	-0.000637998	0.000401805	0.99988191	0.999362205	1.000401885
Right parahippocampal volume (UKB data field 26904)	MR Egger	6	0.002966054	0.007551632	0.714521885	-0.011835144	0.017767252	1.002970457	0.988234615	1.017926028
Right parahippocampal volume (UKB data field 26904)	Weighted median	6	-1.15642E-05	0.000459185	0.979908132	-0.000911568	0.000888439	0.999988436	0.999088848	1.000888834
Right parahippocampal volume (UKB data field 26904)	Inverse variance weighted	6	0.000166663	0.000549647	0.761722988	-0.000910644	0.001243971	1.000166677	0.99908977	1.001244745
Right parahippocampal volume (UKB data field 26904)	Simple mode	6	-0.000571238	0.000955714	0.576074065	-0.002444437	0.001301962	0.999428925	0.997558548	1.00130281
Right parahippocampal volume (UKB data field 26904)	Weighted mode	6	-0.000405656	0.000758994	0.615920392	-0.001893284	0.001081971	0.999594426	0.998108507	1.001082557
Left putamen volume (UKB data field 26560)	MR Egger	29	9.5957E-05	0.000579578	0.869733929	-0.001040016	0.00123193	1.000095962	0.998960525	1.001232689
Left putamen volume (UKB data field 26560)	Weighted median	29	-4.21027E-05	0.000133744	0.752913597	-0.000304242	0.000220036	0.999957898	0.999695805	1.00022006
Left putamen volume (UKB data field 26560)	Inverse variance weighted	29	5.57816E-05	0.000130169	0.66826238	-0.000199349	0.000310912	1.000055783	0.999800671	1.00031096
Left putamen volume (UKB data field 26560)	Simple mode	29	-0.000200144	0.000351289	0.573396361	-0.000888671	0.000488383	0.999799876	0.999111724	1.000488502
Left putamen volume (UKB data field 26560)	Weighted mode	29	-0.000218574	0.000336546	0.52133305	-0.000878205	0.000441057	0.99978145	0.999122181	1.000441154
Left precentral volume (UKB data field 26811)	MR Egger	10	1.8506E-05	0.000103142	0.862067361	-0.000183652	0.000220664	1.000018506	0.999816364	1.000220689
Left precentral volume (UKB data field 26811)	Weighted median	10	5.19272E-05	4.05507E-05	0.200351665	-2.75522E-05	0.000131407	1.000051929	0.999972448	1.000131415
Left precentral volume (UKB data field 26811)	Inverse variance weighted	10	2.23538E-05	5.41891E-05	0.679963017	-8.3857E-05	0.000128564	1.000022354	0.999916147	1.000128573
Left precentral volume (UKB data field 26811)	Simple mode	10	6.3932E-05	9.79043E-05	0.530078676	-0.00012796	0.000255824	1.000063934	0.999872048	1.000255857
Left precentral volume (UKB data field 26811)	Weighted mode	10	5.27834E-05	4.4933E-05	0.270253053	-3.52853E-05	0.000140852	1.000052785	0.999964715	1.000140862
Left precuneus volume (UKB data field 26812)	MR Egger	21	-3.83787E-05	0.000180473	0.833859804	-0.000392105	0.000315348	0.999961622	0.999607971	1.000315398
Left precuneus volume (UKB data field 26812)	Weighted median	21	8.58994E-05	5.5764E-05	0.123460608	-2.33981E-05	0.000195197	1.000085903	0.999976602	1.000195216
Left precuneus volume (UKB data field 26812)	Inverse variance weighted	21	4.16201E-05	4.74383E-05	0.380295865	-5.1359E-05	0.000134599	1.000041621	0.999948642	1.000134608
Left precuneus volume (UKB data field 26812)	Simple mode	21	0.000104501	9.85102E-05	0.301417783	-8.8579E-05	0.000297581	1.000104506	0.999911425	1.000297625
Left precuneus volume (UKB data field 26812)	Weighted mode	21	9.8046E-05	7.82004E-05	0.224380325	-5.52267E-05	0.000251319	1.000098051	0.999944775	1.00025135
Brain stem volume (UKB data field 26526)	MR Egger	64	7.54792E-05	6.55988E-05	0.254308122	-5.30946E-05	0.000204053	1.000075482	0.999946907	1.000204074
Brain stem volume (UKB data field 26526)	Weighted median	64	5.28226E-06	1.81406E-05	0.770909723	-3.02733E-05	4.08378E-05	1.000005282	0.999969727	1.000040839
Brain stem volume (UKB data field 26526)	Inverse variance weighted	64	-3.27521E-05	2.02317E-05	0.105479942	-7.24062E-05	6.90208E-06	0.999967248	0.999927596	1.000006902
Brain stem volume (UKB data field 26526)	Simple mode	64	-4.4789E-05	3.95387E-05	0.26159565	-0.000122285	3.27068E-05	0.999955212	0.999877723	1.000032707
Brain stem volume (UKB data field 26526)	Weighted mode	64	1.64985E-05	3.28702E-05	0.617467202	-4.79271E-05	8.09241E-05	1.000016499	0.999952074	1.000080927
Left medial orbitofrontal volume (UKB data field 26801)	MR Egger	6	-0.00245147	0.002557444	0.392061757	-0.00746406	0.002561121	0.997551533	0.992563727	1.002564404
Left medial orbitofrontal volume (UKB data field 26801)	Weighted median	6	-0.000253037	0.000201929	0.210170168	-0.000648817	0.000142744	0.999746995	0.999351393	1.000142754
Left medial orbitofrontal volume (UKB data field 26801)	Inverse variance weighted	6	-0.000191563	0.000258374	0.458438448	-0.000697976	0.000314849	0.999808455	0.999302268	1.000314898
Left medial orbitofrontal volume (UKB data field 26801)	Simple mode	6	-0.000491951	0.000498663	0.369170506	-0.00146933	0.000485427	0.99950817	0.998531749	1.000485545
Left medial orbitofrontal volume (UKB data field 26801)	Weighted mode	6	-0.000581894	0.000423263	0.227611189	-0.00141149	0.000247701	0.999418275	0.998589506	1.000247732
Left transverse temporal volume (UKB data field 26820)	MR Egger	8	0.001016841	0.001556346	0.537746502	-0.002033597	0.00406728	1.001017359	0.997968469	1.004075562
Left transverse temporal volume (UKB data field 26820)	Weighted median	8	-0.00070175	0.0004631	0.12968791	-0.001609425	0.000205925	0.999298496	0.998391869	1.000205946
Left transverse temporal volume (UKB data field 26820)	Inverse variance weighted	8	-0.000752074	0.000344577	0.029064994	-0.001427444	-7.67041E-05	0.999248209	0.998573574	0.999923299
Left transverse temporal volume (UKB data field 26820)	Simple mode	8	-0.00093135	0.000800348	0.282679415	-0.002500031	0.000637332	0.999069084	0.997503091	1.000637535
Left transverse temporal volume (UKB data field 26820)	Weighted mode	8	-0.000214115	0.000793372	0.795035602	-0.001769124	0.001340893	0.999785908	0.99823244	1.001341793
Right precuneus volume (UKB data field 26913)	MR Egger	20	0.000167171	0.000227	0.470956468	-0.00027775	0.000612091	1.000167185	0.999722289	1.000612279
Right precuneus volume (UKB data field 26913)	Weighted median	20	-3.10089E-05	5.98736E-05	0.604524057	-0.000148361	8.63434E-05	0.999968992	0.99985165	1.000086347
Right precuneus volume (UKB data field 26913)	Inverse variance weighted	20	1.06349E-05	5.14929E-05	0.836376205	-9.02913E-05	0.000111561	1.000010635	0.999909713	1.000111567
Right precuneus volume (UKB data field 26913)	Simple mode	20	-0.000103065	0.00013237	0.445794235	-0.00036251	0.000156379	0.99989694	0.999637556	1.000156391
Right precuneus volume (UKB data field 26913)	Weighted mode	20	2.32561E-05	0.000115931	0.843140896	-0.000203968	0.000250481	1.000023256	0.999796052	1.000250512
Right middle temporal volume (UKB data field 26903)	MR Egger	6	-0.001294394	0.000409373	0.034121998	-0.002096765	-0.000492023	0.998706444	0.997905432	0.999508098
Right middle temporal volume (UKB data field 26903)	Weighted median	6	5.74485E-05	8.40806E-05	0.494445429	-0.000107349	0.000222247	1.00005745	0.999892656	1.000222271
Right middle temporal volume (UKB data field 26903)	Inverse variance weighted	6	4.36582E-06	9.70798E-05	0.964130053	-0.000185911	0.000194642	1.000004366	0.999814107	1.000194661
Right middle temporal volume (UKB data field 26903)	Simple mode	6	0.000104896	0.000146617	0.506344945	-0.000182473	0.000392264	1.000104901	0.999817544	1.000392341
Right middle temporal volume (UKB data field 26903)	Weighted mode	6	0.000104896	0.000155757	0.530525825	-0.000200388	0.000410179	1.000104901	0.999799632	1.000410263
Left pars orbitalis volume (UKB data field 26806)	MR Egger	7	0.000344471	0.004771176	0.945243093	-0.009007033	0.009695976	1.000344531	0.991033408	1.009743134
Left pars orbitalis volume (UKB data field 26806)	Weighted median	7	-0.000220993	0.000426727	0.604542317	-0.001057377	0.000615391	0.999779031	0.998943181	1.000615581
Left pars orbitalis volume (UKB data field 26806)	Inverse variance weighted	7	-0.000352218	0.000543893	0.517251949	-0.001418249	0.000713812	0.999647844	0.998582757	1.000714067
Left pars orbitalis volume (UKB data field 26806)	Simple mode	7	-0.000378264	0.000847962	0.671181757	-0.002040271	0.001283742	0.999621807	0.997961809	1.001284566
Left pars orbitalis volume (UKB data field 26806)	Weighted mode	7	-0.000486363	0.000772397	0.552111953	-0.002000262	0.001027536	0.999513755	0.998001738	1.001028064
Left superior parietal volume (UKB data field 26816)	MR Egger	20	1.89946E-05	0.000125955	0.881807187	-0.000227877	0.000265867	1.000018995	0.999772149	1.000265902
Left superior parietal volume (UKB data field 26816)	Weighted median	20	-3.1128E-05	3.93185E-05	0.42854292	-0.000108192	4.59363E-05	0.999968873	0.999891814	1.000045937
Left superior parietal volume (UKB data field 26816)	Inverse variance weighted	20	-1.69317E-05	3.43446E-05	0.622015636	-8.42472E-05	5.03837E-05	0.999983068	0.999915756	1.000050385
Left superior parietal volume (UKB data field 26816)	Simple mode	20	-4.43361E-05	7.2913E-05	0.550342639	-0.000187246	9.85735E-05	0.999955665	0.999812772	1.000098578
Left superior parietal volume (UKB data field 26816)	Weighted mode	20	-5.08547E-05	6.71115E-05	0.457888597	-0.000182393	8.06839E-05	0.999949147	0.999817623	1.000080687
Left pars triangularis volume (UKB data field 26807)	MR Egger	12	-0.000271626	0.00030348	0.391801125	-0.000866447	0.000323195	0.999728411	0.999133928	1.000323247
Left pars triangularis volume (UKB data field 26807)	Weighted median	12	-0.000247782	0.000122387	0.042910498	-0.000487661	-7.90377E-06	0.999752248	0.999512458	0.999992096
Left pars triangularis volume (UKB data field 26807)	Inverse variance weighted	12	-0.00017846	9.67914E-05	0.065218975	-0.000368171	1.12516E-05	0.999821556	0.999631897	1.000011252
Left pars triangularis volume (UKB data field 26807)	Simple mode	12	-0.000212843	0.000195562	0.29970615	-0.000596145	0.00017046	0.99978718	0.999404033	1.000170474
Left pars triangularis volume (UKB data field 26807)	Weighted mode	12	-0.000237469	0.000174799	0.20150228	-0.000580075	0.000105136	0.999762559	0.999420093	1.000105142
Left superior frontal volume (UKB data field 26815)	MR Egger	22	-0.000227151	0.000104309	0.041573969	-0.000431596	-2.27053E-05	0.999772875	0.999568497	0.999977295
Left superior frontal volume (UKB data field 26815)	Weighted median	22	-4.28715E-05	2.6217E-05	0.101994939	-9.42568E-05	8.51375E-06	0.999957129	0.999905748	1.000008514
Left superior frontal volume (UKB data field 26815)	Inverse variance weighted	22	-4.29054E-05	2.51982E-05	0.088620897	-9.22939E-05	6.48305E-06	0.999957096	0.99990771	1.000006483
Left superior frontal volume (UKB data field 26815)	Simple mode	22	-1.02444E-05	5.52446E-05	0.854665741	-0.000118524	9.80351E-05	0.999989756	0.999881483	1.00009804
Left superior frontal volume (UKB data field 26815)	Weighted mode	22	-5.70626E-05	4.41977E-05	0.210709533	-0.00014369	2.95649E-05	0.999942939	0.99985632	1.000029565
Right lingual volume (UKB data field 26901)	MR Egger	18	-3.33573E-05	0.000173698	0.850125136	-0.000373805	0.00030709	0.999966643	0.999626265	1.000307138
Right lingual volume (UKB data field 26901)	Weighted median	18	-9.97026E-06	5.65242E-05	0.859988008	-0.000120758	0.000100817	0.99999003	0.99987925	1.000100822
Right lingual volume (UKB data field 26901)	Inverse variance weighted	18	9.27498E-06	5.32066E-05	0.861613781	-9.50099E-05	0.00011356	1.000009275	0.999904995	1.000113566
Right lingual volume (UKB data field 26901)	Simple mode	18	-4.13384E-05	8.8053E-05	0.644697524	-0.000213922	0.000131245	0.999958662	0.999786101	1.000131254
Right lingual volume (UKB data field 26901)	Weighted mode	18	-3.04282E-05	7.58593E-05	0.693332938	-0.000179112	0.000118256	0.999969572	0.999820904	1.000118263
Left caudate volume (UKB data field 26559)	MR Egger	27	0.000830102	0.000444813	0.073790299	-4.17323E-05	0.001701935	1.000830446	0.999958269	1.001703384
Left caudate volume (UKB data field 26559)	Weighted median	27	-0.000159356	0.00014104	0.258533546	-0.000435795	0.000117083	0.999840656	0.9995643	1.000117089
Left caudate volume (UKB data field 26559)	Inverse variance weighted	27	-0.00018799	0.000107837	0.081284916	-0.000399351	2.33706E-05	0.999812027	0.999600728	1.000023371
Left caudate volume (UKB data field 26559)	Simple mode	27	-0.000122187	0.000279204	0.665272381	-0.000669427	0.000425053	0.999877821	0.999330797	1.000425144
Left caudate volume (UKB data field 26559)	Weighted mode	27	-0.000145628	0.000283055	0.611258534	-0.000700415	0.00040916	0.999854383	0.99929983	1.000409244
Right cuneus volume (UKB data field 26893)	MR Egger	31	-0.000452921	0.00030427	0.147400975	-0.001049291	0.000143448	0.999547181	0.998951259	1.000143459
Right cuneus volume (UKB data field 26893)	Weighted median	31	3.53191E-05	9.74583E-05	0.717051257	-0.000155699	0.000226337	1.00003532	0.999844313	1.000226363
Right cuneus volume (UKB data field 26893)	Inverse variance weighted	31	3.98125E-05	0.000102577	0.697925203	-0.000161238	0.000240863	1.000039813	0.999838775	1.000240892
Right cuneus volume (UKB data field 26893)	Simple mode	31	5.41206E-05	0.000185609	0.772611511	-0.000309673	0.000417914	1.000054122	0.999690375	1.000418002
Right cuneus volume (UKB data field 26893)	Weighted mode	31	2.25645E-06	0.000139616	0.987212265	-0.000271391	0.000275903	1.000002256	0.999728646	1.000275942
Left caudal anterior cingulate volume (UKB data field 26790)	Wald ratio	1	0.001067259	0.000421633	0.011365681	0.000240858	0.001893661	1.001067829	1.000240887	1.001895455
Right postcentral volume (UKB data field 26910)	MR Egger	13	4.93101E-05	8.70589E-05	0.582493803	-0.000121325	0.000219945	1.000049311	0.999878682	1.00021997
Right postcentral volume (UKB data field 26910)	Weighted median	13	6.44799E-05	4.21761E-05	0.126307986	-1.81853E-05	0.000147145	1.000064482	0.999981815	1.000147156
Right postcentral volume (UKB data field 26910)	Inverse variance weighted	13	1.84441E-05	5.03394E-05	0.714069932	-8.02211E-05	0.000117109	1.000018444	0.999919782	1.000117116
Right postcentral volume (UKB data field 26910)	Simple mode	13	9.33172E-05	8.09834E-05	0.271636671	-6.54102E-05	0.000252045	1.000093322	0.999934592	1.000252076
Right postcentral volume (UKB data field 26910)	Weighted mode	13	6.88881E-05	4.71397E-05	0.169603153	-2.35057E-05	0.000161282	1.000068891	0.999976495	1.000161295
Left lateral orbitofrontal volume (UKB data field 26799)	MR Egger	25	-0.000376368	0.000314604	0.243757097	-0.000992991	0.000240255	0.999623703	0.999007502	1.000240284
Left lateral orbitofrontal volume (UKB data field 26799)	Weighted median	25	-8.16456E-05	8.27086E-05	0.323570144	-0.000243754	8.04632E-05	0.999918358	0.999756275	1.000080466
Left lateral orbitofrontal volume (UKB data field 26799)	Inverse variance weighted	25	-9.81156E-05	7.87189E-05	0.212615839	-0.000252405	5.61734E-05	0.999901889	0.999747627	1.000056175
Left lateral orbitofrontal volume (UKB data field 26799)	Simple mode	25	-3.89062E-06	0.000185358	0.983427387	-0.000367193	0.000359412	0.999996109	0.999632874	1.000359476
Left lateral orbitofrontal volume (UKB data field 26799)	Weighted mode	25	-0.000111292	0.000161358	0.496984948	-0.000427555	0.00020497	0.999888714	0.999572537	1.000204991
Right banks of superior temporal sulcus volume (UKB data field 26890)	MR Egger	3	-0.004388379	0.0033843	0.418214051	-0.011021607	0.002244849	0.995621236	0.989038909	1.002247371
Right banks of superior temporal sulcus volume (UKB data field 26890)	Weighted median	3	0.000121246	0.000522508	0.816501621	-0.000902869	0.001145362	1.000121254	0.999097538	1.001146018
Right banks of superior temporal sulcus volume (UKB data field 26890)	Inverse variance weighted	3	1.22422E-05	0.000431064	0.977343221	-0.000832643	0.000857128	1.000012242	0.999167703	1.000857495
Right banks of superior temporal sulcus volume (UKB data field 26890)	Simple mode	3	0.000227781	0.00068034	0.76962529	-0.001105686	0.001561248	1.000227807	0.998894925	1.001562468
Right banks of superior temporal sulcus volume (UKB data field 26890)	Weighted mode	3	0.000274502	0.000621427	0.701856637	-0.000943496	0.001492499	1.000274539	0.999056949	1.001493614
Left insula volume (UKB data field 26821)	MR Egger	19	-0.0001641	0.000326989	0.622208286	-0.000804999	0.000476798	0.999835913	0.999195325	1.000476912
Left insula volume (UKB data field 26821)	Weighted median	19	-0.000141521	0.000106495	0.183884581	-0.000350252	6.72105E-05	0.999858489	0.999649809	1.000067213
Left insula volume (UKB data field 26821)	Inverse variance weighted	19	-0.000158606	8.61325E-05	0.065559441	-0.000327426	1.02132E-05	0.999841406	0.999672628	1.000010213
Left insula volume (UKB data field 26821)	Simple mode	19	-0.000112663	0.000205575	0.590397479	-0.000515589	0.000290263	0.999887343	0.999484544	1.000290305
Left insula volume (UKB data field 26821)	Weighted mode	19	-0.000117023	0.000216412	0.595316273	-0.000541191	0.000307145	0.999882984	0.999458956	1.000307193
Right paracentral volume (UKB data field 26905)	MR Egger	3	-0.002152527	0.003242657	0.626924001	-0.008508134	0.00420308	0.997849788	0.991527958	1.004211925
Right paracentral volume (UKB data field 26905)	Weighted median	3	-0.000401815	0.000300352	0.180956872	-0.000990504	0.000186874	0.999598266	0.999009986	1.000186892
Right paracentral volume (UKB data field 26905)	Inverse variance weighted	3	-0.000518612	0.000225373	0.021384572	-0.000960344	-7.68805E-05	0.999481522	0.999040116	0.999923122
Right paracentral volume (UKB data field 26905)	Simple mode	3	-0.000265106	0.000385628	0.562806822	-0.001020937	0.000490725	0.999734929	0.998979584	1.000490845
Right paracentral volume (UKB data field 26905)	Weighted mode	3	-0.000275219	0.000364688	0.529206473	-0.000990007	0.000439569	0.999724819	0.999010482	1.000439666
Left supramarginal volume (UKB data field 26818)	MR Egger	12	-0.000155925	0.000152745	0.33139926	-0.000455305	0.000143454	0.999844087	0.999544799	1.000143465
Left supramarginal volume (UKB data field 26818)	Weighted median	12	-2.13392E-05	4.84954E-05	0.659919387	-0.00011639	7.37119E-05	0.999978661	0.999883617	1.000073715
Left supramarginal volume (UKB data field 26818)	Inverse variance weighted	12	-3.86296E-05	4.81442E-05	0.42233774	-0.000132992	5.57331E-05	0.999961371	0.999867017	1.000055735
Left supramarginal volume (UKB data field 26818)	Simple mode	12	-2.29472E-05	7.71034E-05	0.771542028	-0.00017407	0.000128176	0.999977053	0.999825945	1.000128184
Left supramarginal volume (UKB data field 26818)	Weighted mode	12	-3.30308E-05	9.0732E-05	0.722722931	-0.000210866	0.000144804	0.99996697	0.999789157	1.000144814
Right fusiform volume (UKB data field 26895)	MR Egger	5	-7.97701E-05	0.000324731	0.821799799	-0.000716242	0.000556702	0.999920233	0.999284014	1.000556857
Right fusiform volume (UKB data field 26895)	Weighted median	5	6.87992E-05	8.28839E-05	0.406501051	-9.36533E-05	0.000231252	1.000068802	0.999906351	1.000231278
Right fusiform volume (UKB data field 26895)	Inverse variance weighted	5	7.94993E-05	0.000100998	0.431201159	-0.000118457	0.000277455	1.000079502	0.99988155	1.000277494
Right fusiform volume (UKB data field 26895)	Simple mode	5	0.00014744	0.000132966	0.329670089	-0.000113173	0.000408053	1.000147451	0.999886833	1.000408136
Right fusiform volume (UKB data field 26895)	Weighted mode	5	7.11597E-05	9.13031E-05	0.479293573	-0.000107794	0.000250114	1.000071162	0.999892211	1.000250145
Right hippocampus volume (UKB data field 26593)	MR Egger	21	0.000772354	0.000368332	0.049618301	5.04236E-05	0.001494285	1.000772653	1.000050425	1.001495402
Right hippocampus volume (UKB data field 26593)	Weighted median	21	0.000115178	0.000150351	0.443640581	-0.00017951	0.000409865	1.000115184	0.999820506	1.000409949
Right hippocampus volume (UKB data field 26593)	Inverse variance weighted	21	-5.80425E-06	0.000152159	0.969571204	-0.000304035	0.000292426	0.999994196	0.999696011	1.000292469
Right hippocampus volume (UKB data field 26593)	Simple mode	21	-0.000311264	0.000249304	0.226253034	-0.000799899	0.00017737	0.999688784	0.999200421	1.000177386
Right hippocampus volume (UKB data field 26593)	Weighted mode	21	2.61851E-05	0.000252487	0.918433401	-0.00046869	0.00052106	1.000026185	0.99953142	1.000521196
Left paracentral volume (UKB data field 26804)	MR Egger	5	-0.002734497	0.001859655	0.237802253	-0.00637942	0.000910427	0.997269238	0.993640885	1.000910841
Left paracentral volume (UKB data field 26804)	Weighted median	5	-0.000443047	0.000250392	0.076825015	-0.000933815	4.77215E-05	0.999557052	0.999066621	1.000047723
Left paracentral volume (UKB data field 26804)	Inverse variance weighted	5	-0.000425694	0.000213423	0.046085632	-0.000844002	-7.38616E-06	0.999574396	0.999156354	0.999992614
Left paracentral volume (UKB data field 26804)	Simple mode	5	-0.000416371	0.000360247	0.312101977	-0.001122456	0.000289714	0.999583716	0.998878174	1.000289756
Left paracentral volume (UKB data field 26804)	Weighted mode	5	-0.000411116	0.00036554	0.323646611	-0.001127574	0.000305342	0.999588968	0.998873062	1.000305388
Left cuneus volume (UKB data field 26792)	MR Egger	29	-0.00026745	0.00021939	0.233364604	-0.000697454	0.000162554	0.999732586	0.999302789	1.000162568
Left cuneus volume (UKB data field 26792)	Weighted median	29	-9.02019E-05	0.000105783	0.393820213	-0.000297536	0.000117132	0.999909802	0.999702508	1.000117139
Left cuneus volume (UKB data field 26792)	Inverse variance weighted	29	-2.96079E-05	8.70759E-05	0.733838392	-0.000200277	0.000141061	0.999970393	0.999799743	1.000141071
Left cuneus volume (UKB data field 26792)	Simple mode	29	-8.1706E-05	0.000157921	0.608951688	-0.000391232	0.00022782	0.999918297	0.999608844	1.000227846
Left cuneus volume (UKB data field 26792)	Weighted mode	29	-7.45782E-05	0.000109223	0.500338992	-0.000288655	0.000139498	0.999925425	0.999711387	1.000139508
Left postcentral volume (UKB data field 26809)	MR Egger	11	9.33567E-05	0.000102321	0.385351067	-0.000107192	0.000293906	1.000093361	0.999892813	1.000293949
Left postcentral volume (UKB data field 26809)	Weighted median	11	6.50673E-05	4.42693E-05	0.141614512	-2.17006E-05	0.000151835	1.000065069	0.9999783	1.000151847
Left postcentral volume (UKB data field 26809)	Inverse variance weighted	11	4.35085E-06	6.55524E-05	0.947081526	-0.000124132	0.000132834	1.000004351	0.999875876	1.000132842
Left postcentral volume (UKB data field 26809)	Simple mode	11	-1.93973E-05	0.000108068	0.861136364	-0.000231211	0.000192416	0.999980603	0.999768816	1.000192435
Left postcentral volume (UKB data field 26809)	Weighted mode	11	5.40658E-05	4.58153E-05	0.265278136	-3.57323E-05	0.000143864	1.000054067	0.999964268	1.000143874
Left inferior temporal volume (UKB data field 26796)	MR Egger	11	-0.000150953	0.000421065	0.728232263	-0.00097624	0.000674334	0.999849058	0.999024237	1.000674561
Left inferior temporal volume (UKB data field 26796)	Weighted median	11	-1.46781E-05	7.32117E-05	0.841098723	-0.000158173	0.000128817	0.999985322	0.999841839	1.000128825
Left inferior temporal volume (UKB data field 26796)	Inverse variance weighted	11	-6.40593E-05	0.000105282	0.542885728	-0.000270412	0.000142293	0.999935943	0.999729625	1.000142303
Left inferior temporal volume (UKB data field 26796)	Simple mode	11	4.93118E-05	0.000137726	0.727752857	-0.00022063	0.000319254	1.000049313	0.999779394	1.000319305
Left inferior temporal volume (UKB data field 26796)	Weighted mode	11	3.51576E-05	0.000112397	0.760859405	-0.00018514	0.000255456	1.000035158	0.999814877	1.000255488
Right caudal anterior cingulate volume (UKB data field 26891)	Wald ratio	1	5.16588E-05	0.00043049	0.904483148	-0.000792101	0.000895419	1.00005166	0.999208213	1.00089582
Left posterior cingulate volume (UKB data field 26810)	MR Egger	3	0.001860025	0.001108281	0.342091728	-0.000312205	0.004032255	1.001861756	0.999687844	1.004040395
Left posterior cingulate volume (UKB data field 26810)	Weighted median	3	0.000128518	0.000365624	0.725209757	-0.000588104	0.000845141	1.000128527	0.999412069	1.000845498
Left posterior cingulate volume (UKB data field 26810)	Inverse variance weighted	3	-0.000201554	0.00038254	0.598274428	-0.000951333	0.000548224	0.999798466	0.99904912	1.000548375
Left posterior cingulate volume (UKB data field 26810)	Simple mode	3	0.000179532	0.000443568	0.724849464	-0.000689862	0.001048925	1.000179548	0.999310376	1.001049476
Left posterior cingulate volume (UKB data field 26810)	Weighted mode	3	0.000179532	0.000440773	0.723237957	-0.000684384	0.001043447	1.000179548	0.99931585	1.001043992
Right lateral orbitofrontal volume (UKB data field 26900)	MR Egger	23	-0.000827027	0.000266758	0.005419381	-0.001349873	-0.000304182	0.999173315	0.998651038	0.999695864
Right lateral orbitofrontal volume (UKB data field 26900)	Weighted median	23	-0.000223722	8.55943E-05	0.008955384	-0.000391487	-5.59575E-05	0.999776303	0.999608589	0.999944044
Right lateral orbitofrontal volume (UKB data field 26900)	Inverse variance weighted	23	-0.000211774	7.26159E-05	0.003541455	-0.000354101	-6.94467E-05	0.999788249	0.999645962	0.999930556
Right lateral orbitofrontal volume (UKB data field 26900)	Simple mode	23	-0.000154537	0.000177983	0.394619979	-0.000503383	0.000194309	0.999845475	0.999496743	1.000194328
Right lateral orbitofrontal volume (UKB data field 26900)	Weighted mode	23	-0.000225696	0.000139047	0.118798403	-0.000498228	4.68364E-05	0.999774329	0.999501896	1.000046837
Right inferior parietal volume (UKB data field 26896)	MR Egger	12	2.98062E-05	0.00021276	0.891368133	-0.000387203	0.000446816	1.000029807	0.999612872	1.000446916
Right inferior parietal volume (UKB data field 26896)	Weighted median	12	6.95446E-05	4.3003E-05	0.105833847	-1.47412E-05	0.00015383	1.000069547	0.999985259	1.000153842
Right inferior parietal volume (UKB data field 26896)	Inverse variance weighted	12	7.17104E-05	3.72814E-05	0.054418693	-1.36119E-06	0.000144782	1.000071713	0.999998639	1.000144792
Right inferior parietal volume (UKB data field 26896)	Simple mode	12	6.05729E-05	8.2398E-05	0.477643503	-0.000100927	0.000222073	1.000060575	0.999899078	1.000222098
Right inferior parietal volume (UKB data field 26896)	Weighted mode	12	5.71387E-05	7.79953E-05	0.479127251	-9.57321E-05	0.00021001	1.00005714	0.999904273	1.000210032
Right pars orbitalis volume (UKB data field 26907)	MR Egger	6	-0.002042929	0.004148491	0.648206217	-0.010173972	0.006088114	0.997959156	0.989877608	1.006106685
Right pars orbitalis volume (UKB data field 26907)	Weighted median	6	-0.000489529	0.000312696	0.117462815	-0.001102413	0.000123355	0.999510591	0.998898195	1.000123362
Right pars orbitalis volume (UKB data field 26907)	Inverse variance weighted	6	-0.000594483	0.00033579	0.076660426	-0.001252631	6.36656E-05	0.999405694	0.998748153	1.000063668
Right pars orbitalis volume (UKB data field 26907)	Simple mode	6	-3.34131E-05	0.000667259	0.962001269	-0.00134124	0.001274414	0.999966587	0.998659659	1.001275226
Right pars orbitalis volume (UKB data field 26907)	Weighted mode	6	-5.33212E-05	0.000628536	0.935685486	-0.001285253	0.00117861	0.99994668	0.998715573	1.001179305
Left isthmus cingulate volume (UKB data field 26797)	MR Egger	3	-0.016680798	0.024164368	0.615360716	-0.064042959	0.030681363	0.983457556	0.937964704	1.031156887
Left isthmus cingulate volume (UKB data field 26797)	Weighted median	3	0.001079727	0.000464922	0.020212412	0.000168479	0.001990974	1.00108031	1.000168493	1.001992958
Left isthmus cingulate volume (UKB data field 26797)	Inverse variance weighted	3	0.000762505	0.000581219	0.189552041	-0.000376685	0.001901694	1.000762795	0.999623386	1.001903504
Left isthmus cingulate volume (UKB data field 26797)	Simple mode	3	0.001334386	0.000627813	0.167451962	0.000103873	0.002564899	1.001335277	1.000103879	1.002568191
Left isthmus cingulate volume (UKB data field 26797)	Weighted mode	3	0.001327531	0.000617486	0.164548834	0.000117258	0.002537807	1.001328413	1.000117264	1.002541027
Left accumbens area volume (UKB data field 26564)	MR Egger	17	0.004734198	0.002454565	0.072907747	-7.67494E-05	0.009545146	1.004745422	0.999923254	1.009590846
Left accumbens area volume (UKB data field 26564)	Weighted median	17	0.000960657	0.001008115	0.340628415	-0.001015248	0.002936562	1.000961119	0.998985267	1.002940878
Left accumbens area volume (UKB data field 26564)	Inverse variance weighted	17	0.000818549	0.001016636	0.420730546	-0.001174057	0.002811155	1.000818884	0.998826632	1.00281511
Left accumbens area volume (UKB data field 26564)	Simple mode	17	0.001837711	0.002058033	0.385120218	-0.002196033	0.005871455	1.001839401	0.997806377	1.005888726
Left accumbens area volume (UKB data field 26564)	Weighted mode	17	0.003541984	0.001630344	0.045185202	0.000346511	0.006737458	1.003548265	1.000346571	1.006760206
Left entorhinal volume (UKB data field 26793)	MR Egger	4	-0.003569894	0.004562469	0.515883189	-0.012512333	0.005372545	0.99643647	0.987565621	1.005387003
Left entorhinal volume (UKB data field 26793)	Weighted median	4	-0.000141759	0.000365944	0.698475432	-0.00085901	0.000575491	0.999858251	0.999141359	1.000575657
Left entorhinal volume (UKB data field 26793)	Inverse variance weighted	4	-0.00020548	0.000308553	0.505445738	-0.000810243	0.000399284	0.999794542	0.999190085	1.000399364
Left entorhinal volume (UKB data field 26793)	Simple mode	4	-7.10348E-05	0.000507385	0.897529594	-0.001065509	0.000923439	0.999928968	0.998935059	1.000923866
Left entorhinal volume (UKB data field 26793)	Weighted mode	4	-8.17197E-05	0.000523211	0.885802719	-0.001107214	0.000943774	0.999918284	0.998893399	1.00094422
Left superior temporal volume (UKB data field 26817)	MR Egger	12	-0.000262943	0.000144028	0.097871879	-0.000545239	1.93525E-05	0.999737091	0.99945491	1.000019353
Left superior temporal volume (UKB data field 26817)	Weighted median	12	-0.000101838	5.8819E-05	0.083385516	-0.000217123	1.34477E-05	0.999898168	0.999782901	1.000013448
Left superior temporal volume (UKB data field 26817)	Inverse variance weighted	12	-0.000133262	4.16905E-05	0.00139119	-0.000214976	-5.15491E-05	0.999866746	0.999785047	0.999948452
Left superior temporal volume (UKB data field 26817)	Simple mode	12	-3.94402E-05	0.000112946	0.733535932	-0.000260815	0.000181934	0.999960561	0.999739219	1.000181951
Left superior temporal volume (UKB data field 26817)	Weighted mode	12	-2.99065E-05	0.000114519	0.798802018	-0.000254363	0.00019455	0.999970094	0.999745669	1.000194569
Left rostral anterior cingulate volume (UKB data field 26813)	MR Egger	10	0.000439763	0.001089665	0.697096428	-0.00169598	0.002575505	1.000439859	0.998305458	1.002578825
Left rostral anterior cingulate volume (UKB data field 26813)	Weighted median	10	6.94434E-05	0.000236477	0.769018661	-0.000394051	0.000532938	1.000069446	0.999606027	1.00053308
Left rostral anterior cingulate volume (UKB data field 26813)	Inverse variance weighted	10	-0.000194152	0.000259794	0.454865079	-0.000703348	0.000315045	0.999805867	0.999296899	1.000315094
Left rostral anterior cingulate volume (UKB data field 26813)	Simple mode	10	0.000466426	0.000405313	0.279479093	-0.000327988	0.00126084	1.000466535	0.999672066	1.001261635
Left rostral anterior cingulate volume (UKB data field 26813)	Weighted mode	10	0.000446096	0.000551228	0.43922616	-0.000634311	0.001526503	1.000446196	0.99936589	1.001527669
Right accumbens area volume (UKB data field 26595)	MR Egger	18	0.005145889	0.005352623	0.350667435	-0.005345252	0.01563703	1.005159152	0.994669009	1.015759928
Right accumbens area volume (UKB data field 26595)	Weighted median	18	0.001632381	0.001111666	0.141993076	-0.000546485	0.003811247	1.001633714	0.999453664	1.003818519
Right accumbens area volume (UKB data field 26595)	Inverse variance weighted	18	0.000587122	0.001157807	0.6120858	-0.00168218	0.002856424	1.000587295	0.998319234	1.002860508
Right accumbens area volume (UKB data field 26595)	Simple mode	18	0.002304087	0.002542896	0.377553151	-0.002679989	0.007288162	1.002306743	0.997323599	1.007314786
Right accumbens area volume (UKB data field 26595)	Weighted mode	18	0.002068578	0.002392009	0.399194576	-0.002619758	0.006756915	1.002070719	0.99738367	1.006779795
Left banks of superior temporal sulcus volume (UKB data field 26789)	MR Egger	8	-0.001038441	0.001616094	0.544264404	-0.004205985	0.002129103	0.998962098	0.995802847	1.002131371
Left banks of superior temporal sulcus volume (UKB data field 26789)	Weighted median	8	0.000192593	0.000230887	0.404199837	-0.000259946	0.000645133	1.000192612	0.999740088	1.000645341
Left banks of superior temporal sulcus volume (UKB data field 26789)	Inverse variance weighted	8	-0.000118281	0.000229254	0.605896527	-0.000567618	0.000331056	0.999881726	0.999432543	1.000331111
Left banks of superior temporal sulcus volume (UKB data field 26789)	Simple mode	8	0.000266613	0.000342003	0.46118726	-0.000403713	0.000936939	1.000266649	0.999596368	1.000937378
Left banks of superior temporal sulcus volume (UKB data field 26789)	Weighted mode	8	0.000286586	0.000316481	0.395278331	-0.000333717	0.000906888	1.000286627	0.999666339	1.0009073
Right lateral occipital volume (UKB data field 26899)	MR Egger	13	-5.89833E-05	0.000122717	0.640186867	-0.000299509	0.000181543	0.999941018	0.999700535	1.000181559
Right lateral occipital volume (UKB data field 26899)	Weighted median	13	-3.75677E-05	4.61467E-05	0.415590832	-0.000128015	5.28797E-05	0.999962433	0.999871993	1.000052881
Right lateral occipital volume (UKB data field 26899)	Inverse variance weighted	13	-1.68913E-05	3.51467E-05	0.630804101	-8.57789E-05	5.19962E-05	0.999983109	0.999914225	1.000051998
Right lateral occipital volume (UKB data field 26899)	Simple mode	13	-7.27461E-05	7.57666E-05	0.355934987	-0.000221249	7.57564E-05	0.999927257	0.999778776	1.000075759
Right lateral occipital volume (UKB data field 26899)	Weighted mode	13	-7.27461E-05	6.85031E-05	0.309168968	-0.000207012	6.15201E-05	0.999927257	0.999793009	1.000061522
Right transverse temporal volume (UKB data field 26921)	MR Egger	8	0.001005974	0.001885985	0.612930925	-0.002690556	0.004702504	1.00100648	0.997313061	1.004713578
Right transverse temporal volume (UKB data field 26921)	Weighted median	8	2.90619E-05	0.000593277	0.960930879	-0.00113376	0.001191884	1.000029062	0.998866882	1.001192595
Right transverse temporal volume (UKB data field 26921)	Inverse variance weighted	8	-0.000269639	0.000468229	0.56470383	-0.001187367	0.00064809	0.999730398	0.998813337	1.0006483
Right transverse temporal volume (UKB data field 26921)	Simple mode	8	0.000437206	0.000995516	0.67377198	-0.001514005	0.002388417	1.000437301	0.99848714	1.002391271
Right transverse temporal volume (UKB data field 26921)	Weighted mode	8	0.000519477	0.000985223	0.6142995	-0.001411561	0.002450515	1.000519612	0.998589435	1.00245352
Right rostral anterior cingulate volume (UKB data field 26914)	MR Egger	4	0.002731142	0.001196687	0.149966629	0.000385636	0.005076647	1.002734875	1.000385711	1.005089556
Right rostral anterior cingulate volume (UKB data field 26914)	Weighted median	4	-8.56622E-05	0.000304656	0.778574912	-0.000682788	0.000511464	0.999914342	0.999317445	1.000511594
Right rostral anterior cingulate volume (UKB data field 26914)	Inverse variance weighted	4	-0.000383685	0.000414165	0.354234873	-0.00119545	0.000428079	0.999616388	0.998805264	1.00042817
Right rostral anterior cingulate volume (UKB data field 26914)	Simple mode	4	0.000168181	0.000712342	0.828559187	-0.00122801	0.001564372	1.000168195	0.998772744	1.001565596
Right rostral anterior cingulate volume (UKB data field 26914)	Weighted mode	4	0.000197267	0.000420396	0.670894803	-0.000626709	0.001021242	1.000197286	0.999373487	1.001021764
Right caudal middle frontal volume (UKB data field 26892)	MR Egger	11	-1.64952E-05	0.000285683	0.95521772	-0.000576433	0.000543443	0.999983505	0.999423733	1.000543591
Right caudal middle frontal volume (UKB data field 26892)	Weighted median	11	-0.00012701	8.48511E-05	0.134429651	-0.000293318	3.92979E-05	0.999872998	0.999706725	1.000039299
Right caudal middle frontal volume (UKB data field 26892)	Inverse variance weighted	11	-0.000137053	0.000105552	0.194137246	-0.000343936	6.98296E-05	0.999862956	0.999656123	1.000069832
Right caudal middle frontal volume (UKB data field 26892)	Simple mode	11	1.14407E-05	0.000121733	0.926979858	-0.000227156	0.000250037	1.000011441	0.99977287	1.000250068
Right caudal middle frontal volume (UKB data field 26892)	Weighted mode	11	-0.000201561	8.69973E-05	0.04300184	-0.000372075	-3.10462E-05	0.99979846	0.999627994	0.999968954
Right inferior temporal volume (UKB data field 26897)	MR Egger	10	0.000158306	0.000565545	0.78664276	-0.000950163	0.001266774	1.000158318	0.999050288	1.001267577
Right inferior temporal volume (UKB data field 26897)	Weighted median	10	-0.000120884	8.01965E-05	0.131723112	-0.000278069	3.63016E-05	0.999879124	0.99972197	1.000036302
Right inferior temporal volume (UKB data field 26897)	Inverse variance weighted	10	-7.48943E-05	0.000100954	0.458167417	-0.000272764	0.000122975	0.999925109	0.999727274	1.000122983
Right inferior temporal volume (UKB data field 26897)	Simple mode	10	-0.000133392	0.000145831	0.384196991	-0.000419221	0.000152436	0.999866616	0.999580867	1.000152447
Right inferior temporal volume (UKB data field 26897)	Weighted mode	10	-0.000108754	0.000137416	0.449050614	-0.000378088	0.00016058	0.999891252	0.999621983	1.000160593
Right insula volume (UKB data field 26922)	MR Egger	19	-0.000463876	0.000340673	0.191083758	-0.001131594	0.000203843	0.999536232	0.99886904	1.000203864
Right insula volume (UKB data field 26922)	Weighted median	19	-0.000248648	9.57867E-05	0.009435715	-0.00043639	-6.0906E-05	0.999751383	0.999563705	0.999939096
Right insula volume (UKB data field 26922)	Inverse variance weighted	19	-0.000191224	9.76464E-05	0.05019169	-0.000382611	1.63392E-07	0.999808795	0.999617463	1.000000163
Right insula volume (UKB data field 26922)	Simple mode	19	-0.000137335	0.000204577	0.510546644	-0.000538306	0.000263636	0.999862675	0.999461839	1.000263671
Right insula volume (UKB data field 26922)	Weighted mode	19	-0.000460499	0.000177531	0.018332739	-0.000808461	-0.000112538	0.999539607	0.999191866	0.999887468
Right thalamus proper volume (UKB data field 26589)	MR Egger	21	-0.000290089	0.000323749	0.381448263	-0.000924636	0.000344458	0.999709953	0.999075791	1.000344518
Right thalamus proper volume (UKB data field 26589)	Weighted median	21	5.00927E-05	0.000111732	0.653915538	-0.000168902	0.000269087	1.000050094	0.999831113	1.000269123
Right thalamus proper volume (UKB data field 26589)	Inverse variance weighted	21	-3.85617E-07	9.62459E-05	0.996803221	-0.000189027	0.000188256	0.999999614	0.99981099	1.000188274
Right thalamus proper volume (UKB data field 26589)	Simple mode	21	8.99401E-05	0.000226067	0.694957843	-0.000353152	0.000533032	1.000089944	0.999646911	1.000533174
Right thalamus proper volume (UKB data field 26589)	Weighted mode	21	8.3888E-05	0.000188953	0.66183654	-0.000286461	0.000454237	1.000083892	0.999713581	1.00045434

Table S4

outcome	exposure	method	Q	Q_df	Q_pval
Knee osteoarthritis || id:ebi-a-GCST007090	Left pars opercularis volume (UKB data field 26805)	MR Egger	19.19523061	7	0.007597263
Knee osteoarthritis || id:ebi-a-GCST007090	Left pars opercularis volume (UKB data field 26805)	Inverse variance weighted	19.45268813	8	0.012617424
Knee osteoarthritis || id:ebi-a-GCST007090	Right pallidum volume (UKB data field 26592)	MR Egger	69.77122793	28	2.00E-05
Knee osteoarthritis || id:ebi-a-GCST007090	Right pallidum volume (UKB data field 26592)	Inverse variance weighted	70.34583429	29	2.72E-05
Knee osteoarthritis || id:ebi-a-GCST007090	Left hippocampus volume (UKB data field 26562)	MR Egger	29.04446929	18	0.047838285
Knee osteoarthritis || id:ebi-a-GCST007090	Left hippocampus volume (UKB data field 26562)	Inverse variance weighted	48.65491667	19	0.000206168
Knee osteoarthritis || id:ebi-a-GCST007090	Right pars opercularis volume (UKB data field 26906)	MR Egger	23.73737379	5	0.000243848
Knee osteoarthritis || id:ebi-a-GCST007090	Right pars opercularis volume (UKB data field 26906)	Inverse variance weighted	26.44874816	6	0.000183614
Knee osteoarthritis || id:ebi-a-GCST007090	Right isthmus cingulate volume (UKB data field 26898)	MR Egger	12.76183537	6	0.046977839
Knee osteoarthritis || id:ebi-a-GCST007090	Right isthmus cingulate volume (UKB data field 26898)	Inverse variance weighted	12.97077234	7	0.072823728
Knee osteoarthritis || id:ebi-a-GCST007090	Left inferior parietal volume (UKB data field 26795)	MR Egger	12.91831925	7	0.074123713
Knee osteoarthritis || id:ebi-a-GCST007090	Left inferior parietal volume (UKB data field 26795)	Inverse variance weighted	12.92809834	8	0.114347582
Knee osteoarthritis || id:ebi-a-GCST007090	Right superior temporal volume (UKB data field 26918)	MR Egger	17.28531992	10	0.068284211
Knee osteoarthritis || id:ebi-a-GCST007090	Right superior temporal volume (UKB data field 26918)	Inverse variance weighted	19.81406884	11	0.047956701
Knee osteoarthritis || id:ebi-a-GCST007090	Right pericalcarine volume (UKB data field 26909)	MR Egger	91.78260677	40	6.02E-06
Knee osteoarthritis || id:ebi-a-GCST007090	Right pericalcarine volume (UKB data field 26909)	Inverse variance weighted	92.0331048	41	8.67E-06
Knee osteoarthritis || id:ebi-a-GCST007090	Right rostral middle frontal volume (UKB data field 26915)	MR Egger	31.55310049	10	0.000475182
Knee osteoarthritis || id:ebi-a-GCST007090	Right rostral middle frontal volume (UKB data field 26915)	Inverse variance weighted	35.98539882	11	0.000170184
Knee osteoarthritis || id:ebi-a-GCST007090	Left parahippocampal volume (UKB data field 26803)	MR Egger	3.693893417	5	0.594272975
Knee osteoarthritis || id:ebi-a-GCST007090	Left parahippocampal volume (UKB data field 26803)	Inverse variance weighted	4.625252494	6	0.592693328
Knee osteoarthritis || id:ebi-a-GCST007090	Right supramarginal volume (UKB data field 26919)	MR Egger	24.17205137	14	0.043676846
Knee osteoarthritis || id:ebi-a-GCST007090	Right supramarginal volume (UKB data field 26919)	Inverse variance weighted	24.18305664	15	0.062049362
Knee osteoarthritis || id:ebi-a-GCST007090	Left middle temporal volume (UKB data field 26802)	MR Egger	49.16845871	12	1.95E-06
Knee osteoarthritis || id:ebi-a-GCST007090	Left middle temporal volume (UKB data field 26802)	Inverse variance weighted	50.97596531	13	2.03E-06
Knee osteoarthritis || id:ebi-a-GCST007090	Left fusiform volume (UKB data field 26794)	MR Egger	8.436754142	6	0.207819094
Knee osteoarthritis || id:ebi-a-GCST007090	Left fusiform volume (UKB data field 26794)	Inverse variance weighted	8.470980101	7	0.292898718
Knee osteoarthritis || id:ebi-a-GCST007090	Left pericalcarine volume (UKB data field 26808)	MR Egger	68.33217698	35	0.000632524
Knee osteoarthritis || id:ebi-a-GCST007090	Left pericalcarine volume (UKB data field 26808)	Inverse variance weighted	69.12859326	36	0.000739417
Knee osteoarthritis || id:ebi-a-GCST007090	Right precentral volume (UKB data field 26912)	MR Egger	23.23573993	9	0.005688035
Knee osteoarthritis || id:ebi-a-GCST007090	Right precentral volume (UKB data field 26912)	Inverse variance weighted	23.31341197	10	0.009647006
Knee osteoarthritis || id:ebi-a-GCST007090	Right caudate volume (UKB data field 26590)	MR Egger	52.85862722	23	0.000383018
Knee osteoarthritis || id:ebi-a-GCST007090	Right caudate volume (UKB data field 26590)	Inverse variance weighted	54.86981276	24	0.000325935
Knee osteoarthritis || id:ebi-a-GCST007090	Right entorhinal volume (UKB data field 26894)	MR Egger	6.039379565	2	0.04881636
Knee osteoarthritis || id:ebi-a-GCST007090	Right entorhinal volume (UKB data field 26894)	Inverse variance weighted	6.070790098	3	0.108216469
Knee osteoarthritis || id:ebi-a-GCST007090	Left lateral occipital volume (UKB data field 26798)	MR Egger	4.090374283	7	0.769311222
Knee osteoarthritis || id:ebi-a-GCST007090	Left lateral occipital volume (UKB data field 26798)	Inverse variance weighted	5.175789751	8	0.738632902
Knee osteoarthritis || id:ebi-a-GCST007090	Left caudal middle frontal volume (UKB data field 26791)	MR Egger	40.36701571	12	6.25E-05
Knee osteoarthritis || id:ebi-a-GCST007090	Left caudal middle frontal volume (UKB data field 26791)	Inverse variance weighted	40.87035873	13	0.000100011
Knee osteoarthritis || id:ebi-a-GCST007090	Left ventral diencephalon (DC) volume (UKB data field 26565)	MR Egger	84.56707902	33	2.07E-06
Knee osteoarthritis || id:ebi-a-GCST007090	Left ventral diencephalon (DC) volume (UKB data field 26565)	Inverse variance weighted	87.39949889	34	1.38E-06
Knee osteoarthritis || id:ebi-a-GCST007090	Left pallidum volume (UKB data field 26561)	MR Egger	70.69418325	28	1.49E-05
Knee osteoarthritis || id:ebi-a-GCST007090	Left pallidum volume (UKB data field 26561)	Inverse variance weighted	71.47699207	29	1.90E-05
Knee osteoarthritis || id:ebi-a-GCST007090	Right medial orbitofrontal volume (UKB data field 26902)	MR Egger	26.85667218	7	0.000353665
Knee osteoarthritis || id:ebi-a-GCST007090	Right medial orbitofrontal volume (UKB data field 26902)	Inverse variance weighted	38.56256277	8	5.92E-06
Knee osteoarthritis || id:ebi-a-GCST007090	Left lingual volume (UKB data field 26800)	MR Egger	21.47479987	20	0.369675213
Knee osteoarthritis || id:ebi-a-GCST007090	Left lingual volume (UKB data field 26800)	Inverse variance weighted	21.47526801	21	0.430267659
Knee osteoarthritis || id:ebi-a-GCST007090	Left rostral middle frontal volume (UKB data field 26814)	MR Egger	38.12412785	18	0.00372948
Knee osteoarthritis || id:ebi-a-GCST007090	Left rostral middle frontal volume (UKB data field 26814)	Inverse variance weighted	45.15873865	19	0.000651177
Knee osteoarthritis || id:ebi-a-GCST007090	Right posterior cingulate volume (UKB data field 26911)	MR Egger	3.632925429	3	0.303926922
Knee osteoarthritis || id:ebi-a-GCST007090	Right posterior cingulate volume (UKB data field 26911)	Inverse variance weighted	4.159011358	4	0.384913522
Knee osteoarthritis || id:ebi-a-GCST007090	Right amygdala volume (UKB data field 26594)	MR Egger	20.15435557	12	0.064221835
Knee osteoarthritis || id:ebi-a-GCST007090	Right amygdala volume (UKB data field 26594)	Inverse variance weighted	23.06767457	13	0.040871283
Knee osteoarthritis || id:ebi-a-GCST007090	Left thalamus proper volume (UKB data field 26558)	MR Egger	33.66952531	22	0.053056299
Knee osteoarthritis || id:ebi-a-GCST007090	Left thalamus proper volume (UKB data field 26558)	Inverse variance weighted	33.77121741	23	0.068539402
Knee osteoarthritis || id:ebi-a-GCST007090	Right superior frontal volume (UKB data field 26916)	MR Egger	52.57426014	24	0.00065811
Knee osteoarthritis || id:ebi-a-GCST007090	Right superior frontal volume (UKB data field 26916)	Inverse variance weighted	55.29624003	25	0.000449962
Knee osteoarthritis || id:ebi-a-GCST007090	Right ventral diencephalon (DC) volume (UKB data field 26596)	MR Egger	69.96168568	32	0.000119752
Knee osteoarthritis || id:ebi-a-GCST007090	Right ventral diencephalon (DC) volume (UKB data field 26596)	Inverse variance weighted	71.35011335	33	0.000122031
Knee osteoarthritis || id:ebi-a-GCST007090	Left amygdala volume (UKB data field 26563)	MR Egger	34.59024578	14	0.001689673
Knee osteoarthritis || id:ebi-a-GCST007090	Left amygdala volume (UKB data field 26563)	Inverse variance weighted	35.20854511	15	0.002296306
Knee osteoarthritis || id:ebi-a-GCST007090	Right superior parietal volume (UKB data field 26917)	MR Egger	17.01046692	12	0.149204003
Knee osteoarthritis || id:ebi-a-GCST007090	Right superior parietal volume (UKB data field 26917)	Inverse variance weighted	17.32872788	13	0.184708984
Knee osteoarthritis || id:ebi-a-GCST007090	Right pars triangularis volume (UKB data field 26908)	MR Egger	7.264716239	5	0.201685638
Knee osteoarthritis || id:ebi-a-GCST007090	Right pars triangularis volume (UKB data field 26908)	Inverse variance weighted	7.43052047	6	0.282859632
Knee osteoarthritis || id:ebi-a-GCST007090	Right putamen volume (UKB data field 26591)	MR Egger	61.91220558	27	0.000147306
Knee osteoarthritis || id:ebi-a-GCST007090	Right putamen volume (UKB data field 26591)	Inverse variance weighted	62.22473983	28	0.000210075
Knee osteoarthritis || id:ebi-a-GCST007090	Right parahippocampal volume (UKB data field 26904)	MR Egger	14.02478463	4	0.007216372
Knee osteoarthritis || id:ebi-a-GCST007090	Right parahippocampal volume (UKB data field 26904)	Inverse variance weighted	14.50970483	5	0.012676177
Knee osteoarthritis || id:ebi-a-GCST007090	Left putamen volume (UKB data field 26560)	MR Egger	70.97004358	27	8.15E-06
Knee osteoarthritis || id:ebi-a-GCST007090	Left putamen volume (UKB data field 26560)	Inverse variance weighted	70.98337068	28	1.35E-05
Knee osteoarthritis || id:ebi-a-GCST007090	Left precentral volume (UKB data field 26811)	MR Egger	27.35009293	8	0.000614991
Knee osteoarthritis || id:ebi-a-GCST007090	Left precentral volume (UKB data field 26811)	Inverse variance weighted	27.35699307	9	0.001220636
Knee osteoarthritis || id:ebi-a-GCST007090	Left precuneus volume (UKB data field 26812)	MR Egger	30.52575216	19	0.045477056
Knee osteoarthritis || id:ebi-a-GCST007090	Left precuneus volume (UKB data field 26812)	Inverse variance weighted	30.86590516	20	0.056985799
Knee osteoarthritis || id:ebi-a-GCST007090	Brain stem volume (UKB data field 26526)	MR Egger	187.8635278	62	1.35E-14
Knee osteoarthritis || id:ebi-a-GCST007090	Brain stem volume (UKB data field 26526)	Inverse variance weighted	196.9494957	63	1.05E-15
Knee osteoarthritis || id:ebi-a-GCST007090	Left medial orbitofrontal volume (UKB data field 26801)	MR Egger	16.01535766	4	0.002998625
Knee osteoarthritis || id:ebi-a-GCST007090	Left medial orbitofrontal volume (UKB data field 26801)	Inverse variance weighted	19.17543472	5	0.001782729
Knee osteoarthritis || id:ebi-a-GCST007090	Left transverse temporal volume (UKB data field 26820)	MR Egger	4.367977125	6	0.627010165
Knee osteoarthritis || id:ebi-a-GCST007090	Left transverse temporal volume (UKB data field 26820)	Inverse variance weighted	5.726383344	7	0.572036483
Knee osteoarthritis || id:ebi-a-GCST007090	Right precuneus volume (UKB data field 26913)	MR Egger	32.90185467	18	0.017152584
Knee osteoarthritis || id:ebi-a-GCST007090	Right precuneus volume (UKB data field 26913)	Inverse variance weighted	33.81955268	19	0.019298796
Knee osteoarthritis || id:ebi-a-GCST007090	Right middle temporal volume (UKB data field 26903)	MR Egger	3.216938616	4	0.522199421
Knee osteoarthritis || id:ebi-a-GCST007090	Right middle temporal volume (UKB data field 26903)	Inverse variance weighted	13.49622487	5	0.019146867
Knee osteoarthritis || id:ebi-a-GCST007090	Left pars orbitalis volume (UKB data field 26806)	MR Egger	27.26865846	5	5.06E-05
Knee osteoarthritis || id:ebi-a-GCST007090	Left pars orbitalis volume (UKB data field 26806)	Inverse variance weighted	27.38677683	6	0.000122534
Knee osteoarthritis || id:ebi-a-GCST007090	Left superior parietal volume (UKB data field 26816)	MR Egger	32.91363327	18	0.017096497
Knee osteoarthritis || id:ebi-a-GCST007090	Left superior parietal volume (UKB data field 26816)	Inverse variance weighted	33.07499959	19	0.023565675
Knee osteoarthritis || id:ebi-a-GCST007090	Left pars triangularis volume (UKB data field 26807)	MR Egger	7.393545229	10	0.687842382
Knee osteoarthritis || id:ebi-a-GCST007090	Left pars triangularis volume (UKB data field 26807)	Inverse variance weighted	7.498462729	11	0.757400897
Knee osteoarthritis || id:ebi-a-GCST007090	Left superior frontal volume (UKB data field 26815)	MR Egger	38.29972955	20	0.008137675
Knee osteoarthritis || id:ebi-a-GCST007090	Left superior frontal volume (UKB data field 26815)	Inverse variance weighted	44.60622411	21	0.001950183
Knee osteoarthritis || id:ebi-a-GCST007090	Right lingual volume (UKB data field 26901)	MR Egger	30.20234737	16	0.01698085
Knee osteoarthritis || id:ebi-a-GCST007090	Right lingual volume (UKB data field 26901)	Inverse variance weighted	30.32859406	17	0.024070383
Knee osteoarthritis || id:ebi-a-GCST007090	Left caudate volume (UKB data field 26559)	MR Egger	26.57970753	25	0.377201909
Knee osteoarthritis || id:ebi-a-GCST007090	Left caudate volume (UKB data field 26559)	Inverse variance weighted	32.44298995	26	0.17887783
Knee osteoarthritis || id:ebi-a-GCST007090	Right cuneus volume (UKB data field 26893)	MR Egger	67.74772522	29	6.13E-05
Knee osteoarthritis || id:ebi-a-GCST007090	Right cuneus volume (UKB data field 26893)	Inverse variance weighted	74.60633281	30	1.14E-05
Knee osteoarthritis || id:ebi-a-GCST007090	Right postcentral volume (UKB data field 26910)	MR Egger	25.69088335	11	0.007213077
Knee osteoarthritis || id:ebi-a-GCST007090	Right postcentral volume (UKB data field 26910)	Inverse variance weighted	26.14841818	12	0.010226579
Knee osteoarthritis || id:ebi-a-GCST007090	Left lateral orbitofrontal volume (UKB data field 26799)	MR Egger	54.55692678	23	0.000224465
Knee osteoarthritis || id:ebi-a-GCST007090	Left lateral orbitofrontal volume (UKB data field 26799)	Inverse variance weighted	56.53732651	24	0.00019356
Knee osteoarthritis || id:ebi-a-GCST007090	Right banks of superior temporal sulcus volume (UKB data field 26890)	MR Egger	0.70213629	1	0.4020668
Knee osteoarthritis || id:ebi-a-GCST007090	Right banks of superior temporal sulcus volume (UKB data field 26890)	Inverse variance weighted	2.415945531	2	0.298802408
Knee osteoarthritis || id:ebi-a-GCST007090	Left insula volume (UKB data field 26821)	MR Egger	30.77411881	17	0.021273254
Knee osteoarthritis || id:ebi-a-GCST007090	Left insula volume (UKB data field 26821)	Inverse variance weighted	30.77467038	18	0.03056226
Knee osteoarthritis || id:ebi-a-GCST007090	Right paracentral volume (UKB data field 26905)	MR Egger	1.529182446	1	0.216235187
Knee osteoarthritis || id:ebi-a-GCST007090	Right paracentral volume (UKB data field 26905)	Inverse variance weighted	1.920325902	2	0.382830498
Knee osteoarthritis || id:ebi-a-GCST007090	Left supramarginal volume (UKB data field 26818)	MR Egger	21.54370098	10	0.017605622
Knee osteoarthritis || id:ebi-a-GCST007090	Left supramarginal volume (UKB data field 26818)	Inverse variance weighted	22.95929665	11	0.017910196
Knee osteoarthritis || id:ebi-a-GCST007090	Right fusiform volume (UKB data field 26895)	MR Egger	9.000026619	3	0.029290533
Knee osteoarthritis || id:ebi-a-GCST007090	Right fusiform volume (UKB data field 26895)	Inverse variance weighted	9.818462072	4	0.04359925
Knee osteoarthritis || id:ebi-a-GCST007090	Right hippocampus volume (UKB data field 26593)	MR Egger	37.78501203	19	0.006319766
Knee osteoarthritis || id:ebi-a-GCST007090	Right hippocampus volume (UKB data field 26593)	Inverse variance weighted	48.11878532	20	0.000409373
Knee osteoarthritis || id:ebi-a-GCST007090	Left paracentral volume (UKB data field 26804)	MR Egger	3.017853321	3	0.388880728
Knee osteoarthritis || id:ebi-a-GCST007090	Left paracentral volume (UKB data field 26804)	Inverse variance weighted	4.586529196	4	0.332410295
Knee osteoarthritis || id:ebi-a-GCST007090	Left cuneus volume (UKB data field 26792)	MR Egger	49.32007981	27	0.005447084
Knee osteoarthritis || id:ebi-a-GCST007090	Left cuneus volume (UKB data field 26792)	Inverse variance weighted	51.86181658	28	0.003979069
Knee osteoarthritis || id:ebi-a-GCST007090	Left postcentral volume (UKB data field 26809)	MR Egger	30.75922192	9	0.000325539
Knee osteoarthritis || id:ebi-a-GCST007090	Left postcentral volume (UKB data field 26809)	Inverse variance weighted	35.06929784	10	0.00012151
Knee osteoarthritis || id:ebi-a-GCST007090	Left inferior temporal volume (UKB data field 26796)	MR Egger	60.67825787	9	9.92E-10
Knee osteoarthritis || id:ebi-a-GCST007090	Left inferior temporal volume (UKB data field 26796)	Inverse variance weighted	60.98669979	10	2.36E-09
Knee osteoarthritis || id:ebi-a-GCST007090	Left posterior cingulate volume (UKB data field 26810)	MR Egger	0.335962181	1	0.562169252
Knee osteoarthritis || id:ebi-a-GCST007090	Left posterior cingulate volume (UKB data field 26810)	Inverse variance weighted	4.014493342	2	0.134358098
Knee osteoarthritis || id:ebi-a-GCST007090	Right lateral orbitofrontal volume (UKB data field 26900)	MR Egger	31.15230922	21	0.071160224
Knee osteoarthritis || id:ebi-a-GCST007090	Right lateral orbitofrontal volume (UKB data field 26900)	Inverse variance weighted	39.5573869	22	0.012172535
Knee osteoarthritis || id:ebi-a-GCST007090	Right inferior parietal volume (UKB data field 26896)	MR Egger	15.58633	10	0.11210239
Knee osteoarthritis || id:ebi-a-GCST007090	Right inferior parietal volume (UKB data field 26896)	Inverse variance weighted	15.64889637	11	0.154674056
Knee osteoarthritis || id:ebi-a-GCST007090	Right pars orbitalis volume (UKB data field 26907)	MR Egger	10.46745249	4	0.033248289
Knee osteoarthritis || id:ebi-a-GCST007090	Right pars orbitalis volume (UKB data field 26907)	Inverse variance weighted	10.78901825	5	0.055727369
Knee osteoarthritis || id:ebi-a-GCST007090	Left isthmus cingulate volume (UKB data field 26797)	MR Egger	4.099114281	1	0.042905685
Knee osteoarthritis || id:ebi-a-GCST007090	Left isthmus cingulate volume (UKB data field 26797)	Inverse variance weighted	6.236717027	2	0.044229711
Knee osteoarthritis || id:ebi-a-GCST007090	Left accumbens area volume (UKB data field 26564)	MR Egger	45.21301341	15	7.09E-05
Knee osteoarthritis || id:ebi-a-GCST007090	Left accumbens area volume (UKB data field 26564)	Inverse variance weighted	54.26350745	16	4.70E-06
Knee osteoarthritis || id:ebi-a-GCST007090	Left entorhinal volume (UKB data field 26793)	MR Egger	1.185732343	2	0.552740762
Knee osteoarthritis || id:ebi-a-GCST007090	Left entorhinal volume (UKB data field 26793)	Inverse variance weighted	1.732005734	3	0.629841787
Knee osteoarthritis || id:ebi-a-GCST007090	Left superior temporal volume (UKB data field 26817)	MR Egger	9.34020176	10	0.500150066
Knee osteoarthritis || id:ebi-a-GCST007090	Left superior temporal volume (UKB data field 26817)	Inverse variance weighted	10.2250292	11	0.51026654
Knee osteoarthritis || id:ebi-a-GCST007090	Left rostral anterior cingulate volume (UKB data field 26813)	MR Egger	43.88454294	8	5.98E-07
Knee osteoarthritis || id:ebi-a-GCST007090	Left rostral anterior cingulate volume (UKB data field 26813)	Inverse variance weighted	45.86205767	9	6.39E-07
Knee osteoarthritis || id:ebi-a-GCST007090	Right accumbens area volume (UKB data field 26595)	MR Egger	56.0103854	16	2.42E-06
Knee osteoarthritis || id:ebi-a-GCST007090	Right accumbens area volume (UKB data field 26595)	Inverse variance weighted	58.676165	17	1.74E-06
Knee osteoarthritis || id:ebi-a-GCST007090	Left banks of superior temporal sulcus volume (UKB data field 26789)	MR Egger	11.77816231	6	0.0671047
Knee osteoarthritis || id:ebi-a-GCST007090	Left banks of superior temporal sulcus volume (UKB data field 26789)	Inverse variance weighted	12.42902773	7	0.087303823
Knee osteoarthritis || id:ebi-a-GCST007090	Right lateral occipital volume (UKB data field 26899)	MR Egger	6.758727319	11	0.818272117
Knee osteoarthritis || id:ebi-a-GCST007090	Right lateral occipital volume (UKB data field 26899)	Inverse variance weighted	6.886888557	12	0.864996972
Knee osteoarthritis || id:ebi-a-GCST007090	Right transverse temporal volume (UKB data field 26921)	MR Egger	7.023200337	6	0.318706976
Knee osteoarthritis || id:ebi-a-GCST007090	Right transverse temporal volume (UKB data field 26921)	Inverse variance weighted	7.596816791	7	0.369483763
Knee osteoarthritis || id:ebi-a-GCST007090	Right rostral anterior cingulate volume (UKB data field 26914)	MR Egger	2.044936461	2	0.359706006
Knee osteoarthritis || id:ebi-a-GCST007090	Right rostral anterior cingulate volume (UKB data field 26914)	Inverse variance weighted	9.258388532	3	0.026045605
Knee osteoarthritis || id:ebi-a-GCST007090	Right caudal middle frontal volume (UKB data field 26892)	MR Egger	34.87311414	9	6.27E-05
Knee osteoarthritis || id:ebi-a-GCST007090	Right caudal middle frontal volume (UKB data field 26892)	Inverse variance weighted	35.68324006	10	9.54E-05
Knee osteoarthritis || id:ebi-a-GCST007090	Right inferior temporal volume (UKB data field 26897)	MR Egger	38.0787437	8	7.28E-06
Knee osteoarthritis || id:ebi-a-GCST007090	Right inferior temporal volume (UKB data field 26897)	Inverse variance weighted	38.91747169	9	1.19E-05
Knee osteoarthritis || id:ebi-a-GCST007090	Right insula volume (UKB data field 26922)	MR Egger	44.1623686	17	0.000324122
Knee osteoarthritis || id:ebi-a-GCST007090	Right insula volume (UKB data field 26922)	Inverse variance weighted	45.97804728	18	0.000298868
Knee osteoarthritis || id:ebi-a-GCST007090	Right thalamus proper volume (UKB data field 26589)	MR Egger	32.3019711	19	0.028892584
Knee osteoarthritis || id:ebi-a-GCST007090	Right thalamus proper volume (UKB data field 26589)	Inverse variance weighted	33.79617193	20	0.02753702

Table S5				
outcome	exposure	egger_intercept	se	pval
Knee osteoarthritis || id:ebi-a-GCST007090	Left pars opercularis volume (UKB data field 26805)	0.005417486	0.017680422	0.768204316
Knee osteoarthritis || id:ebi-a-GCST007090	Right pallidum volume (UKB data field 26592)	-0.005523621	0.011502647	0.634812532
Knee osteoarthritis || id:ebi-a-GCST007090	Left hippocampus volume (UKB data field 26562)	-0.028219023	0.008094568	0.002636457
Knee osteoarthritis || id:ebi-a-GCST007090	Right pars opercularis volume (UKB data field 26906)	-0.017370007	0.022984581	0.48387518
Knee osteoarthritis || id:ebi-a-GCST007090	Right isthmus cingulate volume (UKB data field 26898)	0.008617556	0.027495231	0.764571118
Knee osteoarthritis || id:ebi-a-GCST007090	Left inferior parietal volume (UKB data field 26795)	0.002139194	0.029386981	0.944006416
Knee osteoarthritis || id:ebi-a-GCST007090	Right superior temporal volume (UKB data field 26918)	0.019976395	0.016515928	0.254279528
Knee osteoarthritis || id:ebi-a-GCST007090	Right pericalcarine volume (UKB data field 26909)	0.002834383	0.008578407	0.742815314
Knee osteoarthritis || id:ebi-a-GCST007090	Right rostral middle frontal volume (UKB data field 26915)	0.025778935	0.021750613	0.263337112
Knee osteoarthritis || id:ebi-a-GCST007090	Left parahippocampal volume (UKB data field 26803)	-0.035760331	0.03705467	0.378833564
Knee osteoarthritis || id:ebi-a-GCST007090	Right supramarginal volume (UKB data field 26919)	0.000936089	0.011724917	0.937496418
Knee osteoarthritis || id:ebi-a-GCST007090	Left middle temporal volume (UKB data field 26802)	0.015747891	0.023710195	0.519132243
Knee osteoarthritis || id:ebi-a-GCST007090	Left fusiform volume (UKB data field 26794)	-0.002090676	0.013400501	0.881138204
Knee osteoarthritis || id:ebi-a-GCST007090	Left pericalcarine volume (UKB data field 26808)	0.004866093	0.007618842	0.527181064
Knee osteoarthritis || id:ebi-a-GCST007090	Right precentral volume (UKB data field 26912)	0.001702032	0.009812784	0.866135826
Knee osteoarthritis || id:ebi-a-GCST007090	Right caudate volume (UKB data field 26590)	-0.014399029	0.015392217	0.359262593
Knee osteoarthritis || id:ebi-a-GCST007090	Right entorhinal volume (UKB data field 26894)	-0.009543894	0.093577002	0.928069174
Knee osteoarthritis || id:ebi-a-GCST007090	Left lateral occipital volume (UKB data field 26798)	0.012913752	0.012395226	0.332128904
Knee osteoarthritis || id:ebi-a-GCST007090	Left caudal middle frontal volume (UKB data field 26791)	0.006467446	0.016719507	0.705661356
Knee osteoarthritis || id:ebi-a-GCST007090	Left ventral diencephalon (DC) volume (UKB data field 26565)	-0.009651789	0.009180635	0.300749482
Knee osteoarthritis || id:ebi-a-GCST007090	Left pallidum volume (UKB data field 26561)	-0.007217265	0.01296157	0.58207401
Knee osteoarthritis || id:ebi-a-GCST007090	Right medial orbitofrontal volume (UKB data field 26902)	0.068656524	0.039305802	0.124184483
Knee osteoarthritis || id:ebi-a-GCST007090	Left lingual volume (UKB data field 26800)	-0.000171125	0.008195486	0.983548007
Knee osteoarthritis || id:ebi-a-GCST007090	Left rostral middle frontal volume (UKB data field 26814)	0.022399049	0.012290605	0.085043048
Knee osteoarthritis || id:ebi-a-GCST007090	Right posterior cingulate volume (UKB data field 26911)	-0.018306598	0.027774542	0.556898119
Knee osteoarthritis || id:ebi-a-GCST007090	Right amygdala volume (UKB data field 26594)	-0.019997056	0.01518329	0.212420284
Knee osteoarthritis || id:ebi-a-GCST007090	Left thalamus proper volume (UKB data field 26558)	0.002353915	0.009131758	0.798978466
Knee osteoarthritis || id:ebi-a-GCST007090	Right superior frontal volume (UKB data field 26916)	0.012388649	0.011113797	0.276013713
Knee osteoarthritis || id:ebi-a-GCST007090	Right ventral diencephalon (DC) volume (UKB data field 26596)	-0.006803497	0.008537402	0.431379534
Knee osteoarthritis || id:ebi-a-GCST007090	Left amygdala volume (UKB data field 26563)	-0.007351012	0.014694694	0.624670751
Knee osteoarthritis || id:ebi-a-GCST007090	Right superior parietal volume (UKB data field 26917)	0.00644132	0.013594107	0.644124346
Knee osteoarthritis || id:ebi-a-GCST007090	Right pars triangularis volume (UKB data field 26908)	-0.004438412	0.013138751	0.749226962
Knee osteoarthritis || id:ebi-a-GCST007090	Right putamen volume (UKB data field 26591)	-0.004092914	0.011086387	0.714870315
Knee osteoarthritis || id:ebi-a-GCST007090	Right parahippocampal volume (UKB data field 26904)	-0.03581687	0.096309827	0.728836109
Knee osteoarthritis || id:ebi-a-GCST007090	Left putamen volume (UKB data field 26560)	-0.001006446	0.01413443	0.943759215
Knee osteoarthritis || id:ebi-a-GCST007090	Left precentral volume (UKB data field 26811)	0.000533234	0.011869253	0.965267706
Knee osteoarthritis || id:ebi-a-GCST007090	Left precuneus volume (UKB data field 26812)	0.005017659	0.010904868	0.650644921
Knee osteoarthritis || id:ebi-a-GCST007090	Brain stem volume (UKB data field 26526)	-0.014220855	0.008212313	0.088309143
Knee osteoarthritis || id:ebi-a-GCST007090	Left medial orbitofrontal volume (UKB data field 26801)	0.069926188	0.078709897	0.424520035
Knee osteoarthritis || id:ebi-a-GCST007090	Left transverse temporal volume (UKB data field 26820)	-0.019860212	0.017039979	0.288047066
Knee osteoarthritis || id:ebi-a-GCST007090	Right precuneus volume (UKB data field 26913)	-0.009865391	0.013923177	0.487674003
Knee osteoarthritis || id:ebi-a-GCST007090	Right middle temporal volume (UKB data field 26903)	0.118175969	0.036859352	0.032709127
Knee osteoarthritis || id:ebi-a-GCST007090	Left pars orbitalis volume (UKB data field 26806)	-0.010986308	0.074651741	0.888749964
Knee osteoarthritis || id:ebi-a-GCST007090	Left superior parietal volume (UKB data field 26816)	-0.003425977	0.011532664	0.769816069
Knee osteoarthritis || id:ebi-a-GCST007090	Left pars triangularis volume (UKB data field 26807)	0.003485231	0.010759885	0.752679236
Knee osteoarthritis || id:ebi-a-GCST007090	Left superior frontal volume (UKB data field 26815)	0.025226871	0.013901198	0.084594721
Knee osteoarthritis || id:ebi-a-GCST007090	Right lingual volume (UKB data field 26901)	0.002816451	0.010890619	0.799231583
Knee osteoarthritis || id:ebi-a-GCST007090	Left caudate volume (UKB data field 26559)	-0.023222763	0.009888914	0.027068923
Knee osteoarthritis || id:ebi-a-GCST007090	Right cuneus volume (UKB data field 26893)	0.015298273	0.008928383	0.097303505
Knee osteoarthritis || id:ebi-a-GCST007090	Right postcentral volume (UKB data field 26910)	-0.004156084	0.009389997	0.66662674
Knee osteoarthritis || id:ebi-a-GCST007090	Left lateral orbitofrontal volume (UKB data field 26799)	0.012745427	0.013948869	0.370341181
Knee osteoarthritis || id:ebi-a-GCST007090	Right banks of superior temporal sulcus volume (UKB data field 26890)	0.066390099	0.050713324	0.415278665
Knee osteoarthritis || id:ebi-a-GCST007090	Left insula volume (UKB data field 26821)	0.000218422	0.012513137	0.986276507
Knee osteoarthritis || id:ebi-a-GCST007090	Right paracentral volume (UKB data field 26905)	0.044888097	0.088754997	0.701909584
Knee osteoarthritis || id:ebi-a-GCST007090	Left supramarginal volume (UKB data field 26818)	0.013283753	0.016387449	0.436458772
Knee osteoarthritis || id:ebi-a-GCST007090	Right fusiform volume (UKB data field 26895)	0.013663858	0.02616028	0.637568574
Knee osteoarthritis || id:ebi-a-GCST007090	Right hippocampus volume (UKB data field 26593)	-0.019773662	0.008674423	0.034361563
Knee osteoarthritis || id:ebi-a-GCST007090	Left paracentral volume (UKB data field 26804)	0.0621907	0.049802056	0.300324575
Knee osteoarthritis || id:ebi-a-GCST007090	Left cuneus volume (UKB data field 26792)	0.008075986	0.006846368	0.248448476
Knee osteoarthritis || id:ebi-a-GCST007090	Left postcentral volume (UKB data field 26809)	-0.011284789	0.010048874	0.29050422
Knee osteoarthritis || id:ebi-a-GCST007090	Left inferior temporal volume (UKB data field 26796)	0.007694336	0.035973255	0.835399626
Knee osteoarthritis || id:ebi-a-GCST007090	Left posterior cingulate volume (UKB data field 26810)	-0.058051292	0.030267369	0.305967669
Knee osteoarthritis || id:ebi-a-GCST007090	Right lateral orbitofrontal volume (UKB data field 26900)	0.026998991	0.011342587	0.026848295
Knee osteoarthritis || id:ebi-a-GCST007090	Right inferior parietal volume (UKB data field 26896)	0.004321271	0.021568152	0.845219509
Knee osteoarthritis || id:ebi-a-GCST007090	Right pars orbitalis volume (UKB data field 26907)	0.026002395	0.074176946	0.743611279
Knee osteoarthritis || id:ebi-a-GCST007090	Left isthmus cingulate volume (UKB data field 26797)	0.298396139	0.413213701	0.601840287
Knee osteoarthritis || id:ebi-a-GCST007090	Left accumbens area volume (UKB data field 26564)	-0.021410161	0.012355772	0.1036331
Knee osteoarthritis || id:ebi-a-GCST007090	Left entorhinal volume (UKB data field 26793)	0.069316235	0.093784256	0.536817111
Knee osteoarthritis || id:ebi-a-GCST007090	Left superior temporal volume (UKB data field 26817)	0.01112225	0.011823971	0.369049677
Knee osteoarthritis || id:ebi-a-GCST007090	Left rostral anterior cingulate volume (UKB data field 26813)	-0.019361852	0.032247632	0.564848969
Knee osteoarthritis || id:ebi-a-GCST007090	Right accumbens area volume (UKB data field 26595)	-0.018932281	0.021695273	0.395763907
Knee osteoarthritis || id:ebi-a-GCST007090	Left banks of superior temporal sulcus volume (UKB data field 26789)	0.021276909	0.03695099	0.585673822
Knee osteoarthritis || id:ebi-a-GCST007090	Right lateral occipital volume (UKB data field 26899)	0.003743548	0.010456951	0.727121228
Knee osteoarthritis || id:ebi-a-GCST007090	Right transverse temporal volume (UKB data field 26921)	-0.012698249	0.018139484	0.51012383
Knee osteoarthritis || id:ebi-a-GCST007090	Right rostral anterior cingulate volume (UKB data field 26914)	-0.077962128	0.029351954	0.117318805
Knee osteoarthritis || id:ebi-a-GCST007090	Right caudal middle frontal volume (UKB data field 26892)	-0.008086663	0.017685494	0.658329572
Knee osteoarthritis || id:ebi-a-GCST007090	Right inferior temporal volume (UKB data field 26897)	-0.016885932	0.040226364	0.685697846
Knee osteoarthritis || id:ebi-a-GCST007090	Right insula volume (UKB data field 26922)	0.011688885	0.013981549	0.414739305
Knee osteoarthritis || id:ebi-a-GCST007090	Right thalamus proper volume (UKB data field 26589)	0.010056541	0.010727088	0.360271418
